# Mimicking a Light‐Harvesting Complex to Accelerate Photooxidation in Asymmetric Lipid Membrane Nanoreactors

**DOI:** 10.1002/anie.1785862

**Published:** 2026-05-02

**Authors:** Julian Bösking, Roland E. P. Nau, Nico Alleva, Richard Jacobi, Tobias Meyer‐Zedler, Hannah Voßhenrich, Francesca Mazotta, Ingo Lieberwirth, David Ng, Michael Schmitt, Jürgen Popp, Leticia González, Tanja Weil, Andrea Pannwitz

**Affiliations:** ^1^ Institute of Inorganic Chemistry I University of Ulm Ulm Germany; ^2^ Synthesis of Macromolecules Max Planck Institute For Polymer Research Mainz Germany; ^3^ Institute of Theoretical Chemistry Faculty of Chemistry University of Vienna Vienna Austria; ^4^ Doctoral School in Chemistry (DoSChem) University of Vienna Vienna Austria; ^5^ Leibniz Institute of Photonic Technology Jena Germany; ^6^ Institute of Physical Chemistry Friedrich Schiller University Jena Jena Germany; ^7^ Abbe Center of Photonics (ACP) Friedrich Schiller University Jena Jena Germany; ^8^ Department of Physical Chemistry of Polymers Max Planck Institute For Polymer Research Mainz Germany; ^9^ Institute of Inorganic and Analytical Chemistry Friedrich‐Schiller‐University Jena Jena Germany; ^10^ Helmholtz Institute for Polymers in Energy Applications Jena (HIPOLE Jena) Jena Germany

**Keywords:** compartmentalization, energy transfer, liposomes, photosensitization, singlet oxygen

## Abstract

In nature, photosynthesis is driven by solar light and a large proportion of the visible spectrum is absorbed by the light harvesting complexes (LHCs), which then transfer the energy to the reaction center. Inspired by nature, we implemented a light harvesting energy transfer cascade within biomimetic lipid bilayers of liposomes built with DPPC (1,2‐dipalmitoyl‐sn‐glycero‐3‐phosphocholine), using membrane‐anchored fluorescein, 2‐(3,6‐dihydroxy‐9H‐xanthen‐9‐yl)‐5‐dodecanamidobenzoic acid (FlC_12_) as primary absorber and membrane anchored eosin Y, hexadecyl 2‐(2,4,5,7‐tetrabromo‐3,6‐dihydroxy‐9H‐xanthen‐9‐yl)benzoate (EYC_16_), as energy acceptor to sensitize oxygen and generate the reactive oxygen species ^1^O_2_. Finally, the model substrate nicotinamide adenine dinucleotide (NADH) is oxidized by ^1^O_2_ within the compartmentalizing liposome nanoreactors. It was observed that our metal‐free LHC system has only a minor effect on the photooxidation rate of NADH when the nanoreactor membrane is functionalized symmetrically. By contrast, asymmetric membrane functionalization of the liposome nanoreactor membranes leads to acceleration by 16% to 27% when using multi‐colored light emitting diodes (LED) or simulated solar light, respectively.

## Graphical abstract

Biomimetic liposome nanoreactors mimicking natural light‐harvesting complexes enable efficient photooxidation of NADH. Membrane‐anchored fluorescein transfers energy via FRET to membrane anchored eosin Y, oxidizing NADH. While symmetric membranes show little improvement, asymmetric chromophore distribution enhances photooxidation rates by up to 27% under simulated sunlight, highlighting spatial organization as a key design principle.

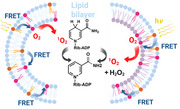

## 1 Introduction

In natural photosynthesis the reaction center of the photosystems I and II (PSI and PSII) are excited mainly by energy transfer from the LHCs which consist of a multitude of carotenes, chlorophylls and other chromophores embedded within the thylakoid lipid bilayer membrane of chloroplasts [1]. The embedding enables a controlled spatial environment for light harvesting. The LHC enables directed energy transfer from the remote light harvesting chromophores to the reaction centers within PS I and PSII [2, 3]. Such long‐range energy transfer is in large parts realized by Förster resonance energy transfer (FRET) processes between various chromophores [3, 4].

In previous work, we already showed that compartmentalization of light‐driven reactions can be significantly accelerated within liposome nanoreactors in comparison to classical homogeneous conditions [5, 6]. These previous studies focused on single chromophore principles and did not include an additional energy transfer cascade. While there are some examples for light harvesting and energy transfer in nanoparticles, and compartmentalization of chemical reactions in drops, micelles, or vesicles, to the best of our knowledge, we are the first to report on a vesicle nanoreactor system, which combines light harvesting energy transfer with a light‐driven photooxidation of water soluble substrate under compartmentalized conditions [7, 8, 9, 10, 11, 12, 13, 14, 15]. Additionally, no rare metals are used in this work, setting us up for sustainability.

## 2 Results and Discussion

**FIGURE 1:**
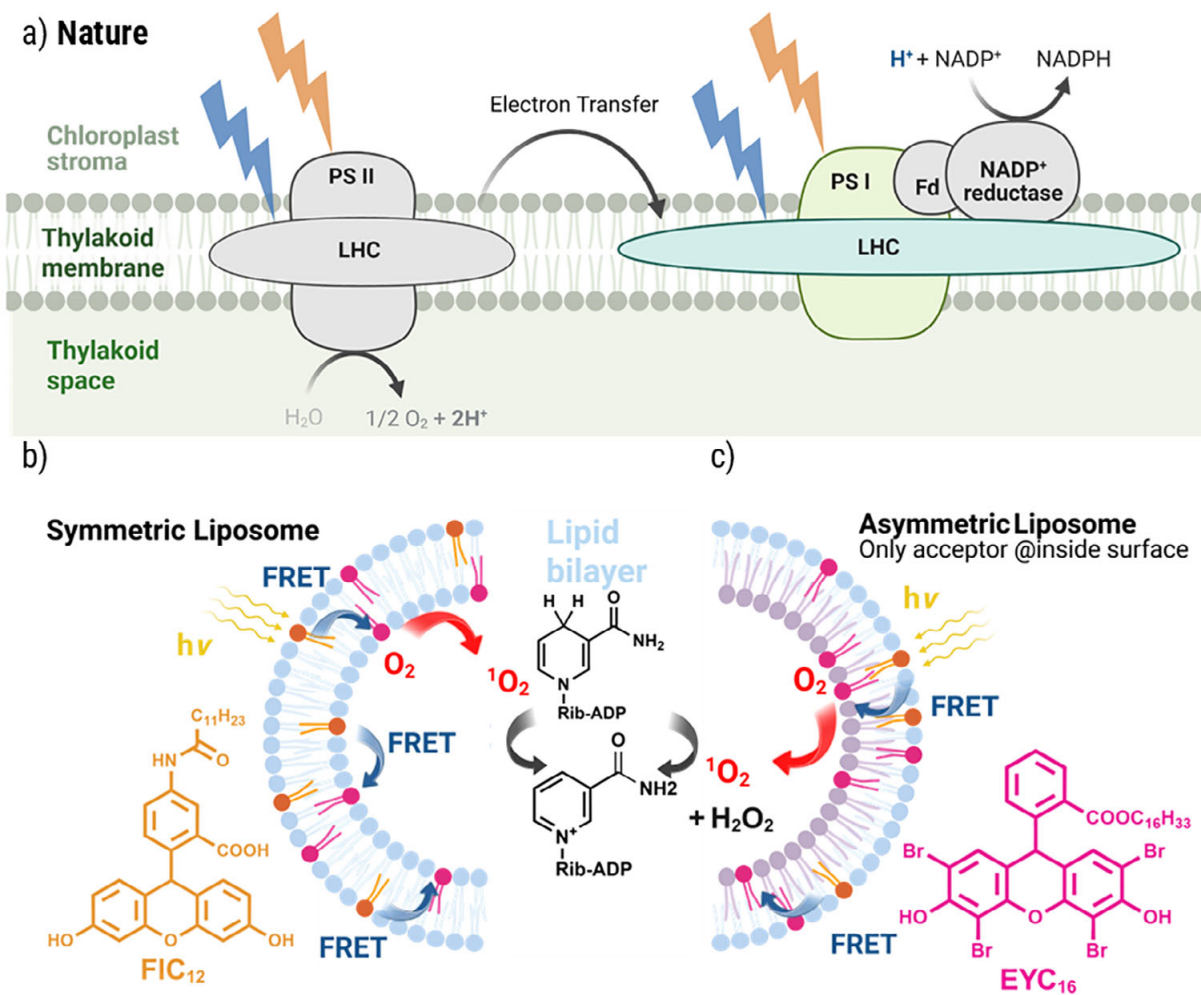
(a) In the thylakoid membrane of chloroplasts, light‐driven reactions yield nicotinamide adenine dinucleotide phosphate (NADPH). After excitation of the LHC, the energy is funneled toward the reaction centers, to subsequently transport electrons for the reduction of NAD^+^. Illustration of (b) symmetric and (c) asymmetric membrane nanoreactors with the primary light absorber and energy donor FlC_12_, the energy acceptor EYC_16_ and the possible directions of energy transfer including FRET and ^1^O_2_ sensitization. Created in BioRender [21].

**FIGURE 2:**
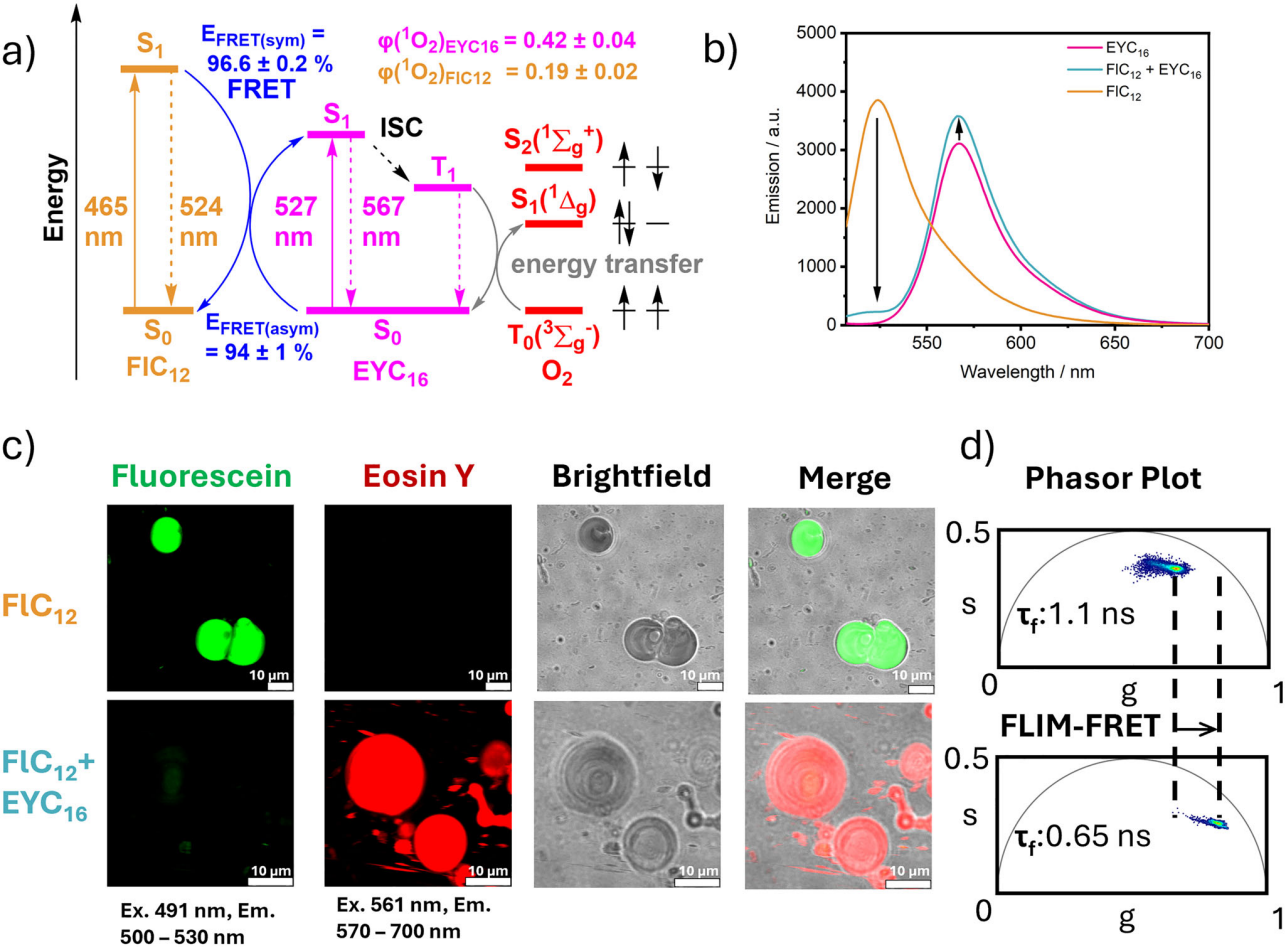
(a) Schematic Jablonski‐type diagram of FlC_12_, EYC_16,_ and oxygen. FlC_12_ can transfer energy (FRET) to EYC_16_ which in turn can sensitize ^3^O_2_ to ^1^O_2_ with Φ (^1^O_2_)_EYC16_ = 0.42 ± 0.04 in methanol‐d4. The ^1^O_2_ sensitization by FlC_12_ with Φ(^1^O_2_)_FlC12_ = 0.19 ± 0.02 in methanol‐d_4_ is omitted for clarity. (b) Quenching of the FlC_12_ emission (yellow) in an asymmetric liposome where EYC_16_ and FlC_12_ are present (teal). (c) Phasor‐fluorescence lifetime imaging of symmetric liposomes loaded with FlC_12_ (2%) (top) and FlC_12_ (2%) + EYC_16_ (1%) (bottom). Donor (FlC_12_) photons are acquired by fast lifetime contrast (FALCON) time correlated single photon counting (TCSPC). FlC_12_ (Fluorescein, Ex. 491 nm, Em. 500–530 nm), EYC_16_ (Eosin Y, Ex. 561 nm, Em. 570–700 nm). Scale bar = 10 µm. (d) Phasor analysis of fluorescence lifetime of FlC_12_. Liposomes loaded with FlC_12_ (2%) (top) and FlC_12_ (2%) + EYC_16_ (1%) (bottom) [22].

Our artificial light harvesting system consists of the hydrophobically functionalized dyes FlC_12_ and EYC_16_, (synthesis see experimental part in the Supporting Information) embedded within the amphiphilic lipid bilayer of DPPC liposome vesicles as shown in Figure 1. Thereby, FlC_12_ absorbs more in the higher energy region of the spectrum of light, is a typical fluorescent singlet emitter, and acts as supporting light absorber and FRET energy donor. EYC_16_ absorbs at lower energy and due to the bromine substituents heavy atoms and hence enhanced spin orbit coupling, the excited triplet state is easily populated, which leads to long‐lived excited states [16]. The latter is very beneficial for the sensitization of ground state oxygen [17] as it allows for high quantum yields of singlet oxygen sensitization of EYC_16_ with Φ(^1^O_2_)_EYC16_ = 0.42 ± 0.04, compared to FlC_12_ with Φ (^1^O_2_)_FlC12_ = 0.19 ± 0.02 (see section S2 for experimental details). The liposome nanoreactors were prepared with the model substrate NADH at 56 mM (section S3) in the inner compartment and irradiated under aerated conditions, in presence of oxygen. The sensitization of oxygen yielded highly reactive singlet oxygen, which is the oxidant in this study, oxidizing NADH to NAD^+^. DPPC was chosen as membrane material because it provides a stable, almost leakage free barrier at room temperature (k_flipflop_ = <0.01 h^−1^, k_flipflop_ is the rate of a molecule wandering from one side of the membrane to the other) between the inner nanoreactor compartment and the bulk aqueous phase [18, 19, 20]. This is supported by a calcein leakage test as shown in the section S4. Additionally, 1% of the sterically stabilizing lipid 1,2‐dimyristoyl‐sn‐glycero‐3‐phosphoethanolamine‐N‐[methoxy(polyethylene glycol)‐2000] ammonium salt (14:0 PEG 2000 PE) was added to the lipid mixture. We investigated symmetric lipid bilayer membranes with FlC_12_ and EYC_16_ being evenly distributed within the lipid bilayer. We also investigated asymmetric lipid bilayer membrane nanoreactors where the inner surface was covered by only the energy acceptor EYC_16_ to investigate the effect or transmembrane energy transfer (asymmetric) vs. statistical energy transfer (symmetric). All synthesis and liposome preparation procedures including full characterization are reported in the experimental part (section S5 and S6). Electron microscopy at cryogenic temperature (cryoEM) confirmed the formation of mostly only unilamellar liposomes (see section S7), while the giant vesicles, shown in the optical microscopy images in Figure 2c are multilamellar due to the respective sample preparations (section S8).

We investigated the energy transfer efficiency via FRET (E_FRET_) from FlC_12_ to EYC_16_. E_FRET_ (Equation S6) was quantified in symmetric (Section S9) and asymmetric liposomes (Figure 2b) via emission intensity quenching yielding E_FRET(sym)_ = 96.6 ± 0.2% and E_FRET(asym)_ 93 ± 1% respectively, demonstrating that almost quantitative energy transfer to the more efficient oxygen sensitizer takes place in both cases. Computation of FRET rates using classical molecular dynamics (MD) simulations of both chromophores in DPPC lipid bilayers (Section S10) revealed that the energy transfer rate for FRET is larger than the fluorescence rate of FlC_12_, confirming that the luminescence quenching is indeed mediated by FRET. MD simulations also predict that the energy transfer rate between FlC_12_ and EYC_16_ in the same leaflet (ca. 15 ps^−1^) is much faster than when the chromophores are in opposite membrane leaflets (ca. 90 ns^−1^). This is in agreement with the experimentally determined energy transfer efficiencies E_FRET_ in symmetric vs. asymmetric vesicles.

The confocal microscopy data on respective large multilamellar vesicles in Figure 2c reveals that the emission of FlC_12_ is almost completely quenched in the presence of the energy acceptor EYC_16_ within the membrane, which is in line with the quantification from liposome samples. In addition to fluorescence intensity quenching, FRET can shorten the excited state lifetime (τ_f_) of the energy donor due to an increased number of excited state relaxation pathways by energy transfer [23] visualized by the phasor‐fluorescence lifetime imaging microscopy (phasor‐FLIM) method. In phasor‐FLIM, the ensemble of measured τ_f_ is plotted according to their real and imaginary components of their Fourier transform in a semicircle phasor plot where the locus of donor photons shifts to lower lifetimes (for details see Section S8) [24]. Vesicles loaded with FlC_12_ exhibited a fluorescence lifetime (τ_f_) of around 1.1 ns (Figure 2d). A slight tailing of the phasor locus and hence a spread of τ_f_ suggests varying hydrophobicity of the local environment around FlC_12_ probably due to different locations of FlC_12_ within multilamellar giant vesicles membranes. Comparatively, symmetric vesicles loaded with both donor and acceptor fluorophores exhibit near quantitative quenching of FlC_12_, along with a significant decrease in τ_f_ to around 0.65 ns. The efficient quenching and FLIM‐FRET observed suggest that the donor and acceptor fluorophores are within close proximity of one another, enabling high light harvesting propensity. Efficient quenching was also observed in homogeneously dispersed liposomes via a Stern–Volmer quenching assay (Section S11), revealing a quenching rate constant of *k*_q_ = 1.7·10^13 ^± 0.2 M^−1^ s^−1^, which is indicative of a preformed arrangement within the membrane, as a diffusion‐based process would be on the order of 10^11^ M^−1^ s^−1^ or slower [25, 26, 27, 28].

**FIGURE 3:**
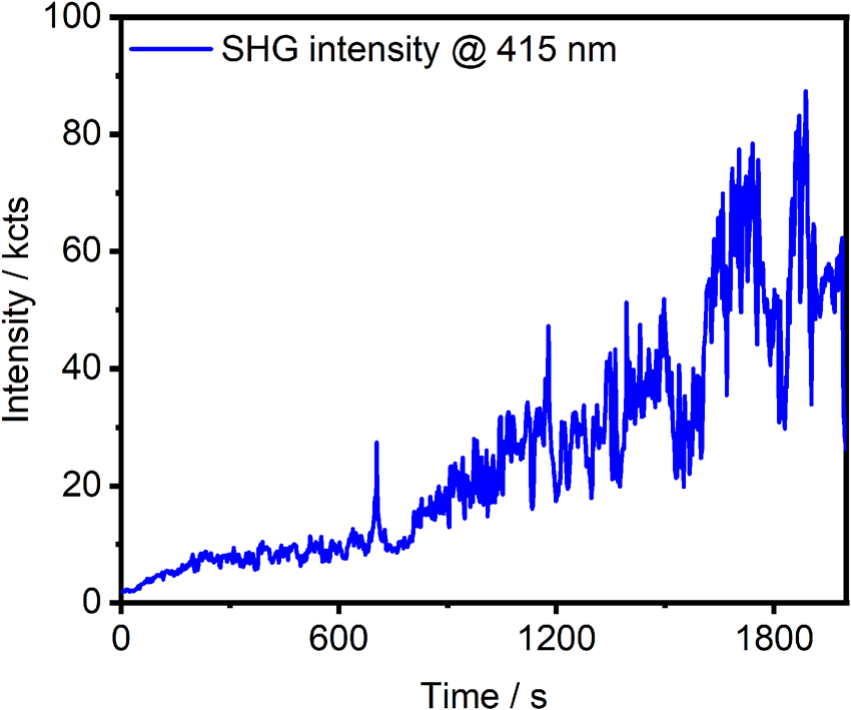
SHG intensity when adding 1% FlC_12_ to DPPC liposome samples at room temperature over time.

**FIGURE 4:**
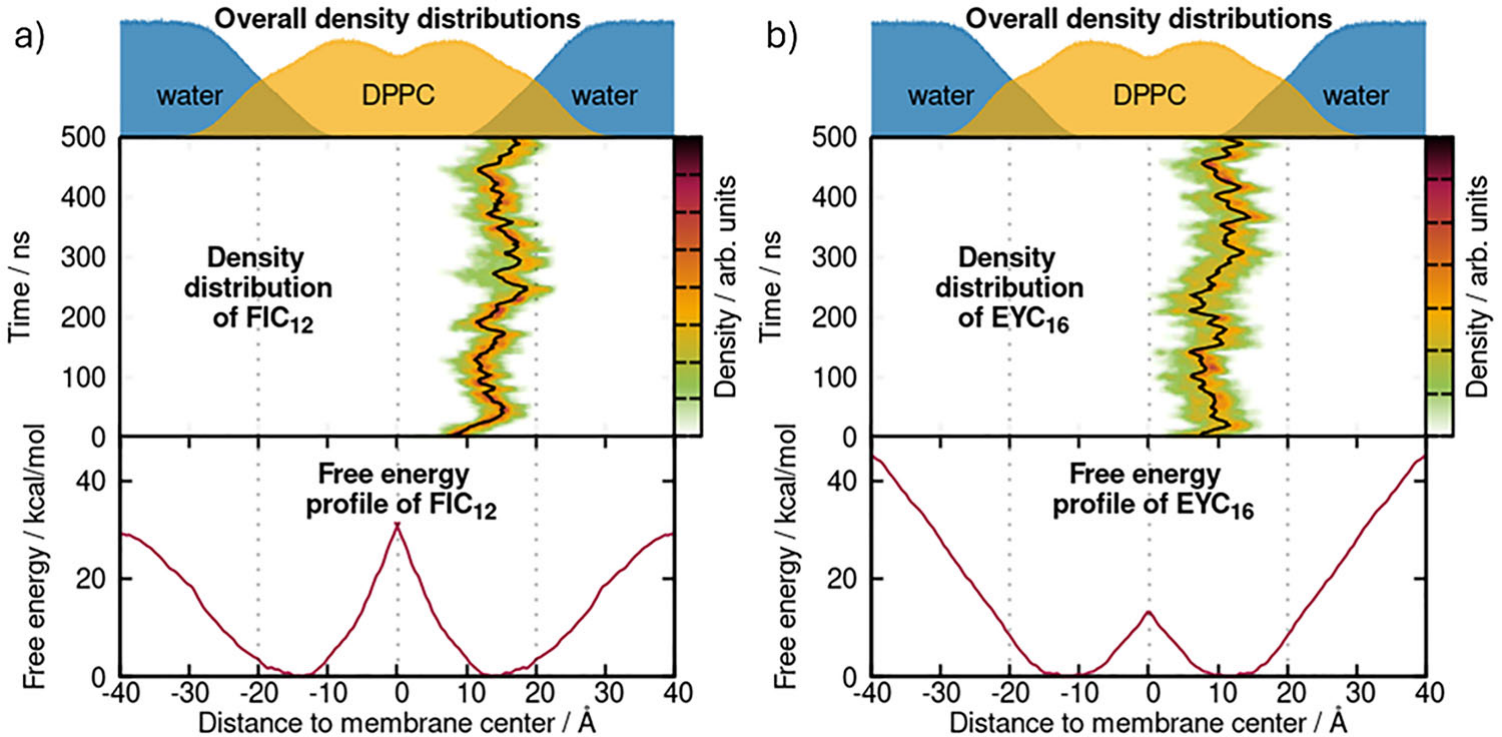
Computed density distributions from 500 ns unconstrained simulation (middle) and free energy profiles (bottom) of (a) FlC_12_ and (b) EYC_16_ in the DPPC membrane, indicated by the overall density distributions (top).

To confirm asymmetry of the here prepared vesicles, we tracked the change of the second harmonic generation (SHG) signal when preparing asymmetric giant vesicles. SHG is generated at interfaces and at asymmetric functionalized interfaces the intensity of SHG is increased in comparison to symmetrically functionalized interfaces [29, 30]. In our SHG experiment we added FlC_12_ in buffer to our vesicle sample and tracked the incorporation via SHG over time (Figure 3, more details in Section S12). The rising SHG signal indicate that FlC_12_ is indeed in the outer membrane leaflet. Unfortunately, prolonged laser irradiation in the SHG‐experiments lead to bleaching of the chromophores, which is why long‐term experiments are not suitable in this case. Apart from SHG we do also see evidence for asymmetry in FLIM data since the fluorescein microenvironment differs significantly for asymmetric and symmetric vesicles. We have investigated the fluorescence lifetime change during preparation of asymmetric vesicles at room temperature for 10 h and observe full functionalization after few hours as well as stability until the end of the imaging experiment. Additionally, the decay traces for symmetric and asymmetric differ, which is expected, when the chromophore is exposed to two different chemical microenvironments. The asymmetric samples are best described by a three‐exponential decay model, whereas symmetrically functionalized nanoreactors were adequately fitted using a bi‐exponential model (see section S12 for more details). Additional proof of asymmetry via this preparation method was generated from classical MD simulations of both chromophores in DPPC lipid bilayers (section S10). The resulting density distributions during unconstrained simulations in Figure 4 show that both chromophores remain embedded in the membrane during these 500 ns. EYC_16_ is embedded closer to the membrane center than FlC_12_, but the density distributions indicate that the chromophores can collide when both are in the same leaflet, which results in the very efficient FRET computed above. To judge whether the chromophores remain in their respective leaflet beyond the simulated 500 ns, we computed the free energy profiles with umbrella sampling simulations. They show a ca. 30 kcal mol^−1^ energy barrier for FlC_12_ crossing the membrane center. This energy barrier is on the same order as the flip flop activation barrier of the host lipid DPPC (29 ± 3 kcal mol^−1^) in its fluid phase [31] indicating a very slow membrane crossing as detailed above. In contrast, the barrier for EYC_16_ crossing the membrane center is merely ca. 13 kcal mol^−1^ indicating that EYC_16_ could cross the membrane faster, irrespective of its initial positioning. Further, for EYC_16_, the barrier for bulk leakage is even higher than that for FlC_12_, signifying that the membrane affinity of EYC_16_ is even higher than that of FlC_12_. Overall, the simulations indicate that the liposomes prepared asymmetrically will remain asymmetrically, because FlC_12_ will not cross the membrane barrier.

To compare our energy‐transfer system to reported benchmarks in compartmentalized photooxidation, an interior NADH concentration of 56 mM was chosen [5, 32]. The absolute amount of detected converted NADH is ∼40 mM, which is lower than the expected 56 mM concentration, assuming that all lipids form liposomes. However, it was reported that there is significant loss of lipids during sample preparation explaining the lower detected amount of encapsulated NADH (section S3) [33]. The ratio between EYC_16_ and FlC_12_ was kept at 1:1. At the same day and time samples of EYC_16_/ FlC_12_/ FlC_12_ + EYC_16_, 14:0 PEG 2000 PE and DPPC in a ratio of (1 or (1 + 1):1:100) were prepared. To ensure solubility and to have the maximum extinction coefficient possible for FlC_12_ we went to pH 9 as lower absorbance means less emission and therefore slower photooxidation (section S13) [34]. The 100 mM concentration stabilizes the liposome size before and after the reaction in dynamic light scattering (DLS) measurements (section S6).

The samples were irradiated within cuvettes that were placed within a self‐designed sample holder, which secured defined and reproducible distance between the light source, optical density filters, and the sample. Detailed construction can be found on github (sections S1.1 and S1.2) [35]. The optical density filters were necessary to reduce the light intensity and to slow down the oxidation speed and minimize dye bleaching during photosensitization, which would slow down photooxidation.

**FIGURE 5:**
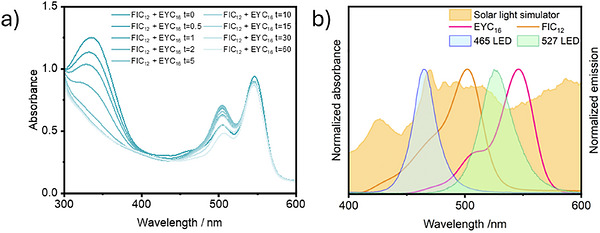
(a) Absorbance over time of a sample with symmetric liposome containing 1% EYC_16_ + 1% FlC_12_ in a DPPC membrane in a 100 mM phosphate buffer at pH 8.99. (b) Overview of emission spectra of LED 465, LED 527 and solar light simulator, with normalized absorbance spectrum of FlC_12_ and EYC_16_ while in a liposome.

The samples were illuminated for 60 min with either LED 527 and/or LED 465 or the solar light simulator (spectra see Figure 5b; section S1.1). The NADH oxidation was traced by UV–vis‐absorption spectroscopy of the characteristic band at 340 nm (Figure 5a; and sections S14 and S15. The reaction of ^1^O_2_ with NADH to H_2_O_2_ and NAD^+^ consumes one proton from the solution, which increases the absorbance of FlC_12_ [34].

**FIGURE 6:**
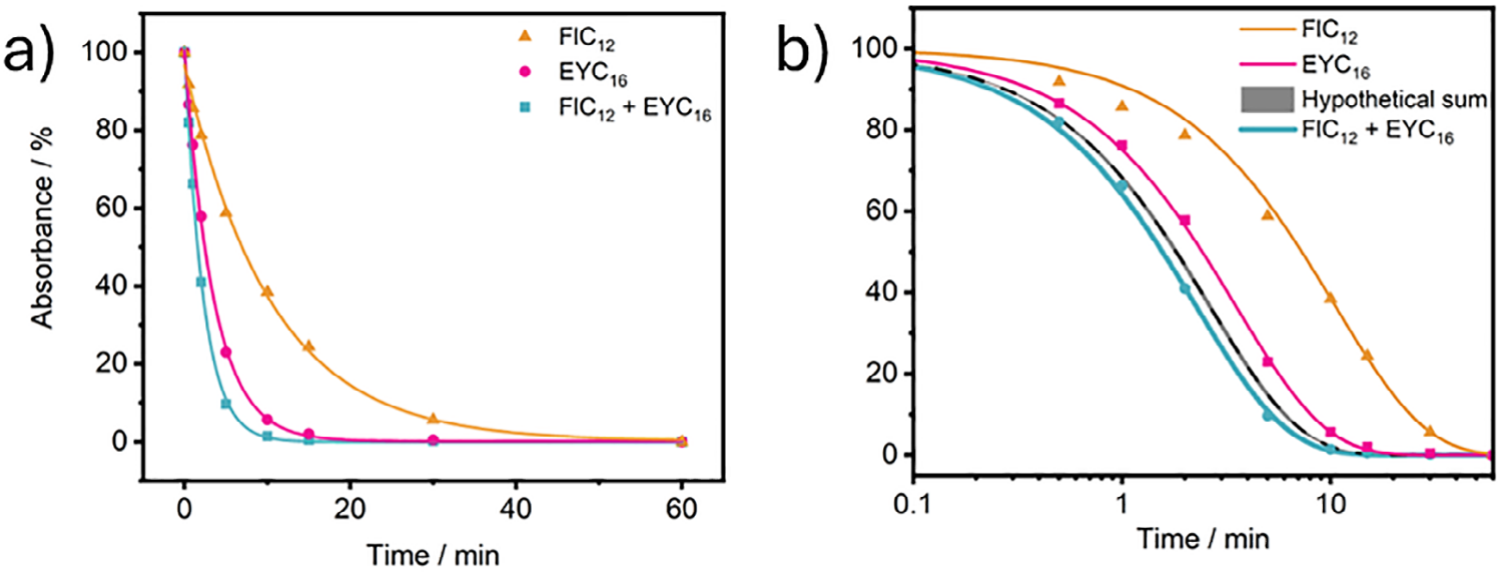
(a), Experiment III in Table 1 UV–vis based NADH decay. (b), Kinetic oxidation of NADH to NAD^+^, plotted with equation 1, with A set to 100% and y_0_ set to zero. Orange = k_FlC12_, purple = k_EYC16_, black = k_hyp_ representing the hypothetical liposome without interaction, grey = maximum error of k_hyp_, teal = k_syn_ (experimental results of the mixture).

At the irradiation time *t* = 0 min, the NADH band at 340 nm was set to 100%. All absorbance bands measured of the respective batch were referred to that value. Under these conditions, without dyes, the autooxidation of NADH is negligible (section S16). The resulting data are shown in Figure 6. The resulting NADH conversion traces were fitted monoexponentially with Equation 1, where *f(t)* represents the relative NADH concentration at irradiation time *t*, *A* representing the starting value of NADH, *k* the rate constant, and the y_0_ intercept as baseline correction.

(1) $$f\;\left(t\right)=A\cdot e^{-kt}+y_{0}$$

To compare the photooxidation performance of the light harvesting system with the sum of the individual chromophores, the performance of a hypothetical liposome containing FlC_12_ and EYC_16_ (k_hyp_) without any interaction was determined by summing up the reaction speed values of samples exclusively containing FlC_12_ (k_FlC12_) and those exclusively containing EYC_16_ (k_EYC16_) (as per Equation 2).

(2) $$k_{hyp}=k_{FlC12}+k_{EYC16}$$

Upper and lower limits for the hypothetical rates were calculated based on the individual standard deviations of k_FlC12_ and k_EYC16_ (Section S15).

Table 1 summarizes the average rate constants of all samples. More data sets can be found in Tables S4 and S5.

**TABLE 1:** Overview of the average rate constants of different systems. Each sample containing either 0.01 eq. FlC_12_ /EYC_16_ or both. Setting them in relation to their hypothetical liposomes created by simple addition of k_FlC12_ and k_EYC16_ or by including the photon distribution (corrected) and summarize the overall efficiency referred to the hypothetical liposomes either as product (column 7 divided by 6) or as difference (column 7 minus 6).

							Speed improvement	
Symmetry^a,b^	Experiment	Correction^c^	FlC_12_ *k_FlC12_* /min^−1^	EYC_16_ *k_EYC16_* /min^−1^	Hypothetical sum *k_hyp_* (k_max_ k_min_) /min^−1^	FlC_12_‐EYC_16_‐liposome *k_syn_* /min^−1^	Factor $\frac{k_{syn}}{k_{HYP}}$($\frac{k_{synmax}}{k_{HYPmax}}$; $\frac{k_{synmin}}{k_{HYPmin}}$)	*Δk (Δk_max;_ Δk_min_)* /min^−1^
520 and 465 nm LED								
sy	Ic	✓			0.72 (0.76; 0.68)		1.05 (1.15; 0.98)	0.04 (0.10; ‐0.03)
	I	✗	0.106 ± 0.006	0.79 ± 0.04	0.89 (0.94; 0.84)	0.75 ± 0.02	0.84 (0.92; 0.77)	−0.14 (‐0.07; ‐0.21)
ay	IIc	✓			0.67 (0.70; 0.65)		1.16 (1.26; 1.08)	0.11 (0.17; 0.05)
	II	✗	0.053 ± 0.004	0.71 ± 0.03	0.76 (0.79; 0.73)	0.78 ± 0.03	1.03 (1.11; 0.95)	0.02 (0.08; ‐0.04)
465 nm LED								
sy	III	✗	0.092 ± 0.007	0.286 ± 0.007	0.38 (0.39; 0.36)	0.44 ± 0.01	1.16 (1.24; 1.08)	0.06 (0.09; 0.03)
Solar light simulator								
ay	IV	✗	0.133 ± 0.010	1.7 ± 0.1	1.87 (1.98; 1.77)	2.4 ± 0.2	1.27 (1.43; 1.12)	0.50 (0.76; 0.23)

^a,b^ = (sy = symmetric; ay = asymmetric)

^c^ = photon competition corrected

In the symmetric experiment I with illumination 465 nm and 520 nm LEDs, an apparent worsening of the NADH oxidation speed by 14% (entry I in Table 1) is observed. However, when correcting the data for the actually absorbed photons of the relatively sharp LED‐emission profiles and the shadowing effect in the absorption profiles of EYC_16_ and FlC_12_, the following correction is necessary: FlC_12_ has access to 64% of photons while EYC_16_ has access to 83% of the incoming photons compared to single dye experiments. Those factors were applied to the individual *k* to calculate the corrected *k_HYP_* (Equation S22–S24, section S17). If the correction due to photon competition is considered, the light symmetrical harvesting nanoreactors improve in photooxidation by 5% (entry Ic in Table 1).

**FIGURE 7:**
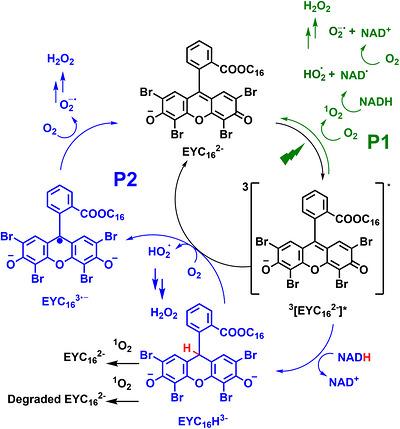
Proposed mechanism of light driven NADH oxidation. EYC_16_^2−^ is excited by light to ^3^[EYC_16_^2−^]*. P1 is the production of ^1^O_2_ and regeneration of EYC_16_^2−^. Then the ^1^O_2_ reacts with NADH to NAD˙, producing HO_2_˙. NAD˙ reacts with ^3^O_2_ to NAD^+^ and O_2_^−^. All oxygen radicals (O_2_^−^˙ and HO_2˙_) will turn into H_2_O_2_ through subsequent reactions. P2 is the reaction of ^3^[EYC_16_^2−^]* with NADH to EYC_16_H^3−^ and NAD^+^. Then, EYC_16_H^3−^ reacts with O_2_ and ^3^[EYC_16_^2−^]* to EYC_16_^3^˙^−^, HO_2_˙, and EYC_16_^2−^ or with ^1^O_2_ to either EYC_16_^2−^ or degraded EYC_16_^2−^. The radical EYC_16_^3^˙^−^ is regenerated by ^3^O_2_ to EYC_16_^2−^ and O_2_^−^˙ [43, 44, 45]. Adapted from Ref. [36].

This improvement cannot be adapted to a liposome‐free homogeneous solution due to the depletion of the eosin Y absorbance band (without C_16_ chain) in presence of NADH (section S18) [6]. An explanation is the interaction of NADH as hydride donor with eosin Y leading to an obliterated absorbance which disables the dye as emitter [36, 37]. We could isolate the formed reduced eosin Y in an experiment where we used 1 mM eosin Y and 56 mM NADH in D_2_O under irradiation (section S19). In emission lifetime experiments, we could see that in the presence of NADH (regardless if O_2_ is present or not) the lifetime of EYC_16_ embedded in the membrane increases by 20% from 1.2 to 1.5 ns. This is suggesting reductive quenching of the excited state EYC_16_ by NADH, generating EYC_16_H^3−^ (sections S19 and S20 and Figure 7). Because we are in a self‐quenching regime of eosin Y (section S21), the average distance of not reduced EYC_16_ increases and therefore the self‐quenching decreases. According to Kim et al., the reduced eosin Y is then regenerated by O_2_ and not reduced eosin Y in an autocatalytic way [36, 37, 38]. Experiments where we deployed eosin Y in the bulk and 56 mM NADH inside the liposomes show that instead of a few minutes the NADH is oxidized in ∼60 min (section S22). If we deploy 1% EYC_16_ in the membrane and deploy 0.1 mM NADH in the bulk (same mol as we would have in the inner compartment), the oxidation of NADH has slightly lower speed (section S23). Therefore, both pathways of reducing eosin Y via NADH and afterward recovery of eosin Y with ^1^O_2_ (Pathway 2 (P2) Figure 7) and sensitized ^1^O_2_ which in turn oxidizes NADH should be possible (Pathway 1 (P1) Figure 7). Experiments where sodium azide as ^1^O_2_ quencher was added to liposomes support that singlet oxygen is needed for NADH oxidation (section S24) [39, 40, 41, 42]. Quantification of P1 vs. P2 was not performed as a multitude of effects are overlapping and affecting the necessary transient absorption experiments including self‐quenching within the membrane and stray light‐effects from the liposomes.

The photon correction method was supported by the following set of experiments (experiment III in Table 1). Samples with FlC_12_ liposomes, EYC_16_ liposomes and symmetric FlC_12_EYC_16_ liposomes were only irradiated with 465 nm LEDs where the energy donor absorbs to a major extend and EYC_16_ has a smaller absorption (spectra see Figure 5b). It was found that the symmetric light harvesting liposomes improve in NADH photooxidation by 16% compared to the sum of the individual contributions by FlC_12_ and EYC_16_. This experiment demonstrated that FlC_12_ indeed acts as a light harvester and transfers energy to the energy acceptors EYC_16_ and which in turn generates the oxidant ^1^O_2_ (P1) or photooxidizes NADH directly (P2).

A major influence on variation of samples was probably the uptake of FlC_12_, which showed a significant variation over all experiments. On top of that, bleaching of the dyes during illumination as well as small local pH variations, can affect the spectral properties and hence the overall performance. By working in a dark and climatized environment, we ensured comparability for all samples. In addition, the used dyes are prone to self‐quenching processes within the membrane which can lead to “dead” photosensitization cycles (section S21) [46].

As described in a previous study, the reaction speed of the photoinduced NADH conversion is faster if the photosensitizer is in close proximity to the NADH substrate [5]. The energy donor is necessary to increase the absorption window, but its close proximity to the NADH is not. Therefore, addressing the outer surface is of special interest. Due to a membrane thickness of <5 nm FRET occurs. This emulation of the spatial separation inspired by nature leads to a directed net energy transfer from LHC modified outer surface to the inner liposome compartment where NADH is located.

Sample II is illuminated with two LEDs (465 and 527 nm). Here, without taking into consideration the competition of the chromophores for photons, a minor improvement of 3% is seen compared to an apparent worsening in sample I. In these kinds of samples, EYC_16_ can use 91% and FlC_12_ can use 55% of the incoming photons. After taking this into account, an acceleration of 16% is seen in sample IIc. As the uptake of FlC_12_ is worse than in the symmetric liposomes, either lower local concentration or the asymmetricity is beneficial for the reaction speed. Lower local concentration lowers the self‐quenching of FlC_12_.

In a more application related approach, we switched the illumination source to a solar light simulator where the samples are illuminated with the simulated spectrum and power of one sun (Air Mass 1.5 Global filter (AM 1.5G), 100 mW cm^−2^). The reaction speed of each individual sample increases in the sample IV. This is related to the higher illumination intensity compared to the LEDs which deliver about 60% less energy (Table S6). In sample IV a synergistic speed improvement of 27% compared to the hypothetical *k_HYP_* was seen, which is faster than the LEDs.

## 3 Conclusion

In summary, we showed that light‐driven oxidation of the NADH model substrate with two metal free dyes, FlC_12,_ and EYC_16_ is fast in biomimetic nanoreactor compartments, when asymmetric, while in homogenous solution the photosensitizer is reduced. This reaction is only possible in liposomes. Two pathways are possible for the oxidation of NADH. We showed that an energy transfer cascade leads to more efficient oxygen sensitization by EYC_16_. While the symmetric liposomes exhibit only small improvements, asymmetric liposomes with FlC_12_ positioned only on the outer membrane surface, exhibit significant gains and synergistic effects. We could prove that the membrane indeed is asymmetric via experimental and theoretical evidence. The here reported artificial light harvesting system proves that the efficiency of photooxidation reactions can be significantly enhanced and that the positioning and local concentration of the energy donor is important. This study provides design principles for efficient use of solar light in sustainable artificial photosynthesis systems, which will lead to more efficient production of solar fuels and other synthetic products.

## Author Contributions

J.B., R.E.P.N., and H.V. synthesized functionalized liposomes and performed and analyzed spectroscopic measurements for characterization and photooxidation. J.B. performed time resolved and steady state spectroscopy for mechanistic insights. R.E.P.N. synthesized the EYC_16_. A.P. supervised synthesis, photooxidation and spectroscopic measurements. N.A. and D.G. performed and analyzed the Phasor‐FLIM experiments. R.J. performed and analyzed the computations and simulations. L.G. supervised the computations and simulations. T.M. performed and analyzed the SHG measurements. M.S. and J.P. supervised the SHG measurements. F.M. performed and analyzed the cryoEM experiments. I.L. supervised the cryoEM experiments. J.P., L.G., T.W., and A.P. acquired funding. T.W. and A.P. envisioned the project. All authors contributed to the final version of the manuscript.

## Conflicts of Interest

The authors declare no conflicts of interest.

## Supporting information




**Supporting File 1**: The authors have cited additional references within the Supporting Information [27, 33, 35, 36, 47, 48, 49, 50, 51, 52, 53, 54, 55, 56, 57, 58, 59, 60, 61, 62, 63, 64, 65, 66, 67, 68, 69, 70, 71, 72, 73, 74, 75, 76, 77, 78, 79, 80, 81, 82, 83, 84, 85, 86]. The technical details of the photoreactor for two‐color irradiation are reported on github via [https://github.com/Pannwitz‐group/Simic‐box‐2023. [35]

## Data Availability

The data that supports the findings of this study are available in the Supporting Information of this article.
