# Supplementary material for: Mimicking a Light‐Harvesting Complex to Accelerate Photooxidation in Asymmetric Lipid Membrane Nanoreactors
Source: Angew Chem Int Ed Engl. 2026 May 2;65(25):e1785862. doi: 10.1002/anie.1785862 (PMC13266925; doi:10.1002/anie.1785862)
Supplement: Supplementary file 1 — Supporting File 1: The authors have cited additional references within the Supporting Information [27, 33, 35, 36, 47, 48, 49, 50, 51, 52, 53, 54, 55, 56, 57, 58, 59, 60, 61, 62, 63, 64, 65, 66, 67, 68, 69, 70, 71, 72, 73, 74, 75, 76, 77, 78, 79, 80, 81, 82, 83, 84, 85, 86]. The technical details of the photoreactor for two‐color irradiation are reported on github via [https://github.com/Pannwitz‐group/Simic‐box‐2023. [35] [file ANIE-65-e1785862-s001.pdf]

Table of Contents

S1. Chemicals and equipment 4
1.1 Light sources 6
1.2 Setup/ Simic-Box 8
1.3 Synthesis EYC_{16} 11
1.4 NMR of FlC_{12} 12
S2. Singlet oxygen emission quantum yields 13
S3. NADH concentration experiment 15
S4. Calcein leakage test. 16
S5. Sample preparation 19
5.1 Preparation of the Phosphate Buffer 19
5.2 Liposome preparation 19
S6. Exemplary dynamic light scattering (DLS) 21
S7. Cryo-electron microscopy (Cryo-EM) sample preparation and imaging 22
S8. Phasor-fluorescence lifetime imaging microscopy 23
S9. Energy transfer efficiency E_{FRET} 25
S10. MD simulations and computation of the FRET rate 26
S11. Stern Volmer Quenching 30
S12. Second harmonic generation and fluorescence lifetime imaging 33
S13. Absorbance and emission at different pH 38
S14. Exemplary spectra for NADH conversion 39
14.1 Symmetric example Nr. 14 39
14.2 Asymmetric example Nr. 30 41
14.3 Asymmetric example Nr.42 42
S15. Rate constants 44
15.1 Symmetric Liposomes 45
15.2 Asymmetric Liposomes 50
S16. Measurements without fluorophores (autooxidation). 55
S17. Photon competition 56
S18. Protection of eosin Y from NADH due to membrane 62
S19. Hydride transfer from NADH to eosin Y - NMR experiments. 63
S20. EYC_{16} lifetimes in liposomes 65

S21. Self Quenching of EosinY 67
S22. 1% eosin Y in the bulk 56 mM NADH inside 68
S23. 0.1 mM NADH in bulk, 1% EYC_{16} in membrane 70
S24. Sodium azide tests 72
S25. References 72

S1. Chemicals and equipment

Reduced nicotinamide adenine dinucleotide (NADH), disodium salt was purchased from abcr GmbH. $Ru(bpy)_3Cl_2$ (RuC_0), EosinY disodium salt, 1-bromo-n-hexadecane, chloroform (GC grade), methanol (GC grade) as well as deuterated methanol and water (99.9 atom %) were purchased from Merck. 5-dodecanoylaminofluorescein was purchased from Invitrogen by Thermo Fisher Scientific. Di-potassium hydrogen phosphate trihydrate was purchased from Supelco. Potasium dihydrogen phosphate was bought from Application Chemicals LTD. The lipids DPPC (1,2-dipalmitoyl-sn-glycero-3-phosphocholine) and 14:0 PEG 2000 PE (1,2-dimyristoyl-sn-glycero-3-phosphoethanolamine-N-[methoxy(polyethylene glycol)-2000] (ammonium salt) were purchased form Avanti Polar Lipids through Merck.

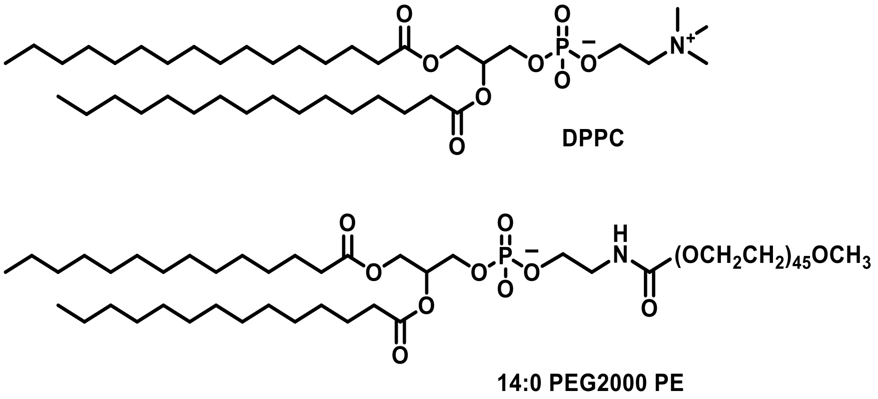

Figure S 1: Lipids used in this study.

Extrusion occurred with a mini extruder from Avanti Polar Lipids purchased through Merck. If not noted differently, all spectroscopic measurements were performed in quartz glass cuvettes (d = 10.0 mm). The DLS measurements for determination of the hydrodynamic radius (ZAvg) and the polydispersity index (PDI) were performed with a Zetasizer Pro-Red from Malvern in a quartz glass cuvette (d = 10.0 mm). UV-vis absorption spectroscopy was performed either on a V-670 JASCO UV-VIS-NIR Spectrometer, on a V-760 JASCO UV-VIS-NIR or on an Avantes AvaSpec-ULS2048CL detector unit coupled with an AVA AvaLight-DH-S-BAL light source. Light source, cuvette holder and detector were connected with fibreoptic cables. Afterwards Microsoft Excel was used to correct the lamp switch of the V-670 Jasco UV-Vis-Nir spectrometer which occurs by 340 nm, by subtracting from the data points between 250 nm and 340 nm the difference between 340 nm and 340.5 nm or Spectrophotometer without any further corrections.

Emission spectroscopy in the UV-vis region was performed on either a JASCO FP-8500 Spectrofluorometer or on Horiba Jobin-Yvon FluoroMax Plus-C automated benchtop Spectrofluorometer equipped with a 150 W Xe arc excitation lamp, a R13456 photomultiplier tube detector (190-930 nm). For the phosphorescence of $_1O^2$ at around 1270 nm it was reequipped with a liquid-nitrogen cooled DSS-IGA020L InGaAs photodiode detector (800-1550 nm) and Czerny-Turner monochromator with NIR grating blazed at 1000 nm as well as a DeltaDiode laser 481 nm.

Fluorescence lifetimes were recorded with a DeltaPro from Horiba Scientific using a 451 nm pulsed Laser source (Class 3B Laser Product, <0.5 W peak in pulsed and CW mode). The Delta Pro consists of: DeltaDiode (Picosecond diode controller), DeltaHub (High throughput TCSPC controller), DPS-1 (Detector Power supply) and a PPD (Picosecond photon detection module). The IRF (Instrument response function) was measured with LUDOX silica nanoparticles. Fits were done with the software EzTime with 2 exponentials.

Phasor-fluorescence lifetime imaging microscopy is performed on inverted Leica Stellaris® 8 microscope (40x glycerol immersion objective) with fast lifetime contrast (FALCON) module (Leica Microsystems GmbH). Pulse excitation is performed using a 80 MHz white light laser tuned to 491 nm for fluorescein and 561 nm for eosin Y. Emitted photons were detected using HyD® X (GaAsP hybrid photocathode) detector with a filter window of 500 – 530 nm (fluorescein), 570 – 700 nm (eosin Y). Acquisition was performed at 512 x 512 pixels at 100 Hz. Photons were counted based on FALCON-modified time correlated single photon counting (TCSPC) method.

CryoEM micrographs Quantifoil 2/1 Cu 400 mesh grid were glow discharged in a 6:1 oxygen: hydrogen plasma (Diener Nano®, Diener electronic, Germany). 4 µL of the solution was then applied on the treated grid and blotted for 4 sec with a force parameter of 0 and then plunged into liquid ethane using the automated plunging system (Vitrobot Thermo-Fisher Scientific). The grid is then transferred in a Titan Krios G4 (Thermo-Fisher Scientific). The images were acquired at an electron voltage of 300 kV using a direct electron detection camera (Gatan K3) with a 20eV energy filter.

Size exclusion chromatography was performed with following equipment: A superfine G25 Sephadex column (6 cm length, 2 cm diameter) with phosphate buffer (see chapter 5.1) as mobile phase was used to exchange the surrounding solvent of the liposomes by the eluent.

For irradiation a solar light simulator Type LSE140 from LOT Quantum Design with a xenon lamp was used.

The emission spectrum of the solar light simulator was measured using an Avantes AvaSpec-ULS2048CL detector unit connected with a fibreoptic cable. To reduce the intensity a metallic ND Filter, UV Fused Silica, 50.8x50.8 mm, 1.0 OD at 632.8 nm from mks Newport optical density filter was used in front of the fiber cable.

If not otherwise mentioned data were fitted with Origin2021b (64-bit) 9.8.5.201 (Lehre)

1.1 Light sources

For irradiation two LED sources were used. Circuit diagram can be found in the attachment. For blue light irradiation LED of type NSPB500AS from Nichia Corporation were used. For the green light irradiation LEDs from Winger Electronics type WEEG40-CS were used.

The LED sticks were measured with a Hioki 3664 optical power meter set to the respective maximum emission wavelength in combination with the Hioki 9742 optical sensor the position of the optical density filter, see technical drawing in the attachment.

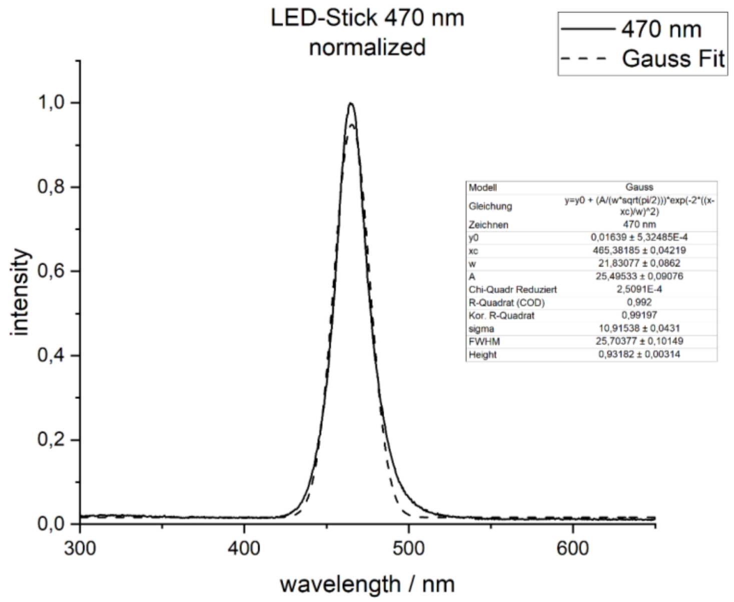

Figure S2: Normalized emissions spectrum of the applied LED-light source with gauss fit: λ_{max} = 465.4 nm, full width half maximum (FWHM) = 26 nm, maximum power = 49.4 mWcm^{-2} and an average power of 39.1 mWcm^{-2}.

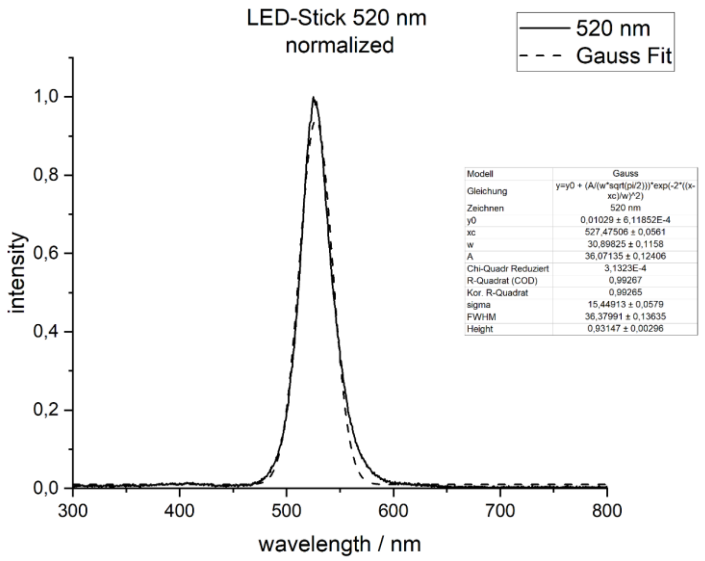

Figure S3: Normalized emissions spectrum of the applied LED-light source with gauss fit: λ_{max} = 527.5 nm, full width half maximum (FWHM) = 36 nm, maximum power = 20.6 mWcm^{-2} and an average power of 19.5 mWcm^{-2}.

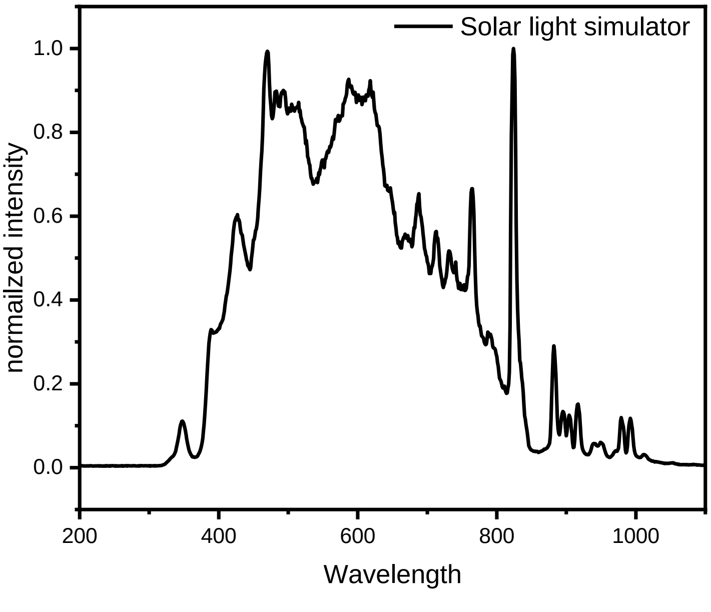

Figure S4: Normalized emission spectrum of the applied xenon lamp of the solar light simulator.

1.2 Setup/ Simic-Box

Two Metallic ND Filter, UV Fused Silica, 50.8x50.8 mm, 1.0 OD at 632.8 nm from mks Newport were used to reduce the illumination intensity and prevent fast photobleaching of the chromophores which was observed in absence of any filter.

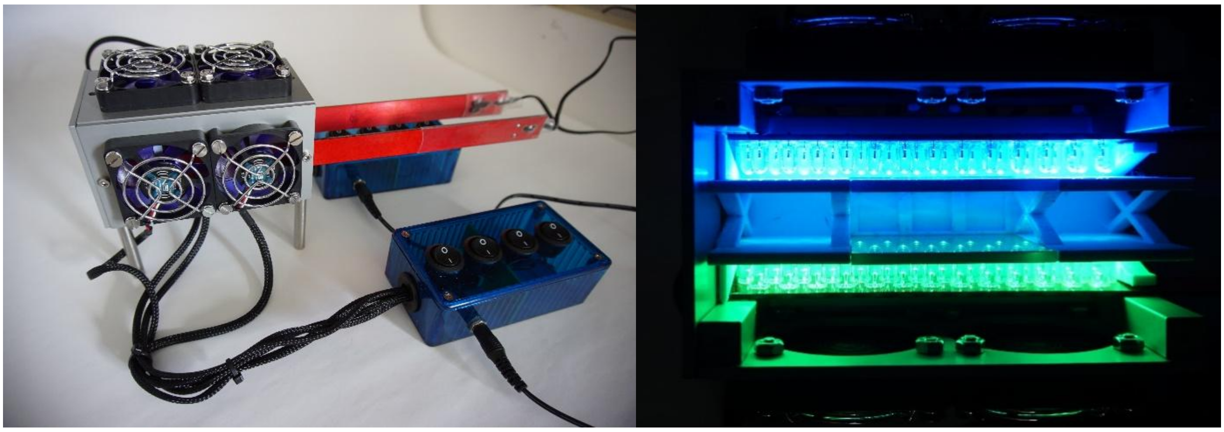

Pictures taken by Marius Müßler.

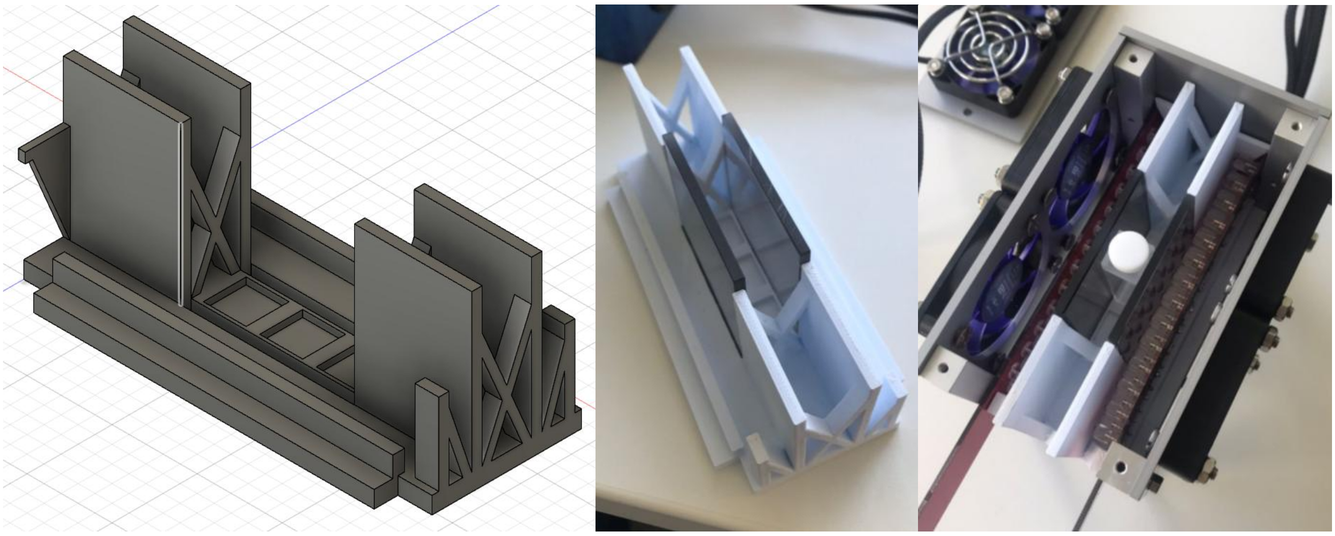

Figure S5: Top left: ready to use setup, containing fan control. Top right: reaction chamber, with cuvette holder and optical density filter in use. Bottom left: Three dimensional corpus of the measuring insert. Bottom centre: 3D-printed cuvette and LED holder. Bottom right: total setup arrangement.

Technical drawings of the metal constructed box as well as the 3D model of the cuvette holder can be found on github.[1]

Measuring spot comparison

The synthetic approach reported in chapter 2 was doubled down in one flask with **Y** and **X** equal to 50. Sample set Q in Table S4. In contrast to the reported procedure the first fraction was diluted with 10 mM phosphorous buffer pH 9.01 to 9 ml and well mixed. The solution was than equally spitted among three samples, each corresponding to one illumination spot.

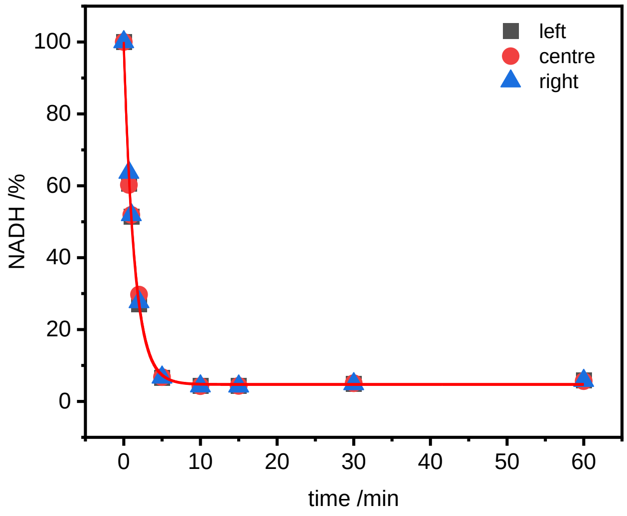

Figure S6: Temporal evolution of the NADH emission intensities at 465 nm and mono exponential decay fitting three Simic-Box cuvettes spots.

Position	t /min^{-1}
left	1.37 ± 0.04
centre	1.41 ± 0.06
right	1.41 ± 0.02

Measuring Spot comparison

The synthetic approach reported in chapter 2 was doubled down in one flask with **Y** and **X** equal to 50. In contrast to the reported procedure the first fraction was diluted with 10 mM phosphorous buffer pH 9.01 to 6 ml and well mixed. The solution was than equally spitted among two samples, each corresponding to one illumination spot. The illumination power was adjusted 100 mWcm^{-2} at both spots.

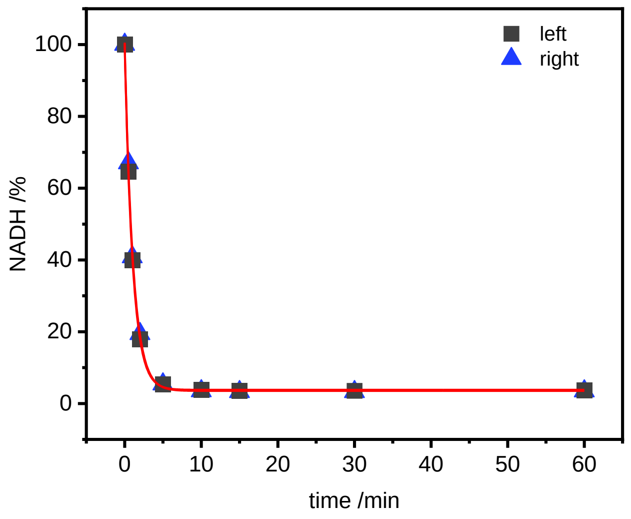

Figure S7: Temporal evolution of the NADH emission intensities with the solar light simulator and mono exponential decay fitting two solar light cuvettes spots. Sample Q

Position	t /min^{-1}
left	0.96 ± 0.02
right	0.91 ± 0.03

1.3 Synthesis EYC_{16}

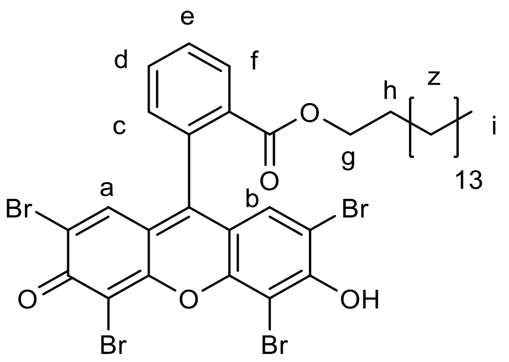

According to literature, 2.08 g of eosinY disodium salt (3 mmol, 1.0 eq.) were solved in 50 ml DMF.[2] To the solution 0.92 mL of 1-bromo-n-hexadecane (3 mmol, 1.0 eq.) was added. The reaction mixture was stirred for 3 h at 100°C. After cooling down the reaction mixture to room temperature 50 ml of dichloromethane were added. The reaction mixture was transferred in a separating funnel and intensively mixed. Afterwards 10 ml of 1M hydrochloric acid was added. The organic layer was washed with 50 ml of water four times. The organic layer was dried in vacuum. The red wax like product was purified via column chromatography. SiO2 as stationary phase, ethyl acetate/acetone (1:1, v:v). The product containing fraction was dried in the rotor vaporizer followed by resolving in 80 ml of dichloromethane. To the solution hexane was added, resulting in precipitating of the product.

^{1}H NMR (400 MHz, MeOH-*d4*, TMS) δ [ppm] = 8.21 (dd, J = 7.7, 1.5 Hz, 1H, H_f), 7.79 (td, J = 7.5, 1.5 Hz, 1H, H_d), 7.73 (td, J = 7.6, 1.4 Hz, 1H, H_e), 7.41 (dd, J = 7.5, 1.4 Hz, 1H, H_c), 7.18 (s, 2H, $H_{a/b}$), 3.91 (t, J = 6.2 Hz, 2H, H_g), 1.43 – 0.96 (m, 24H, H_z), 0.94 – 0.82 (m, 5H, $H_{h/i}$).

1.4 NMR of FlC12

FLC12 was bought and used as received.

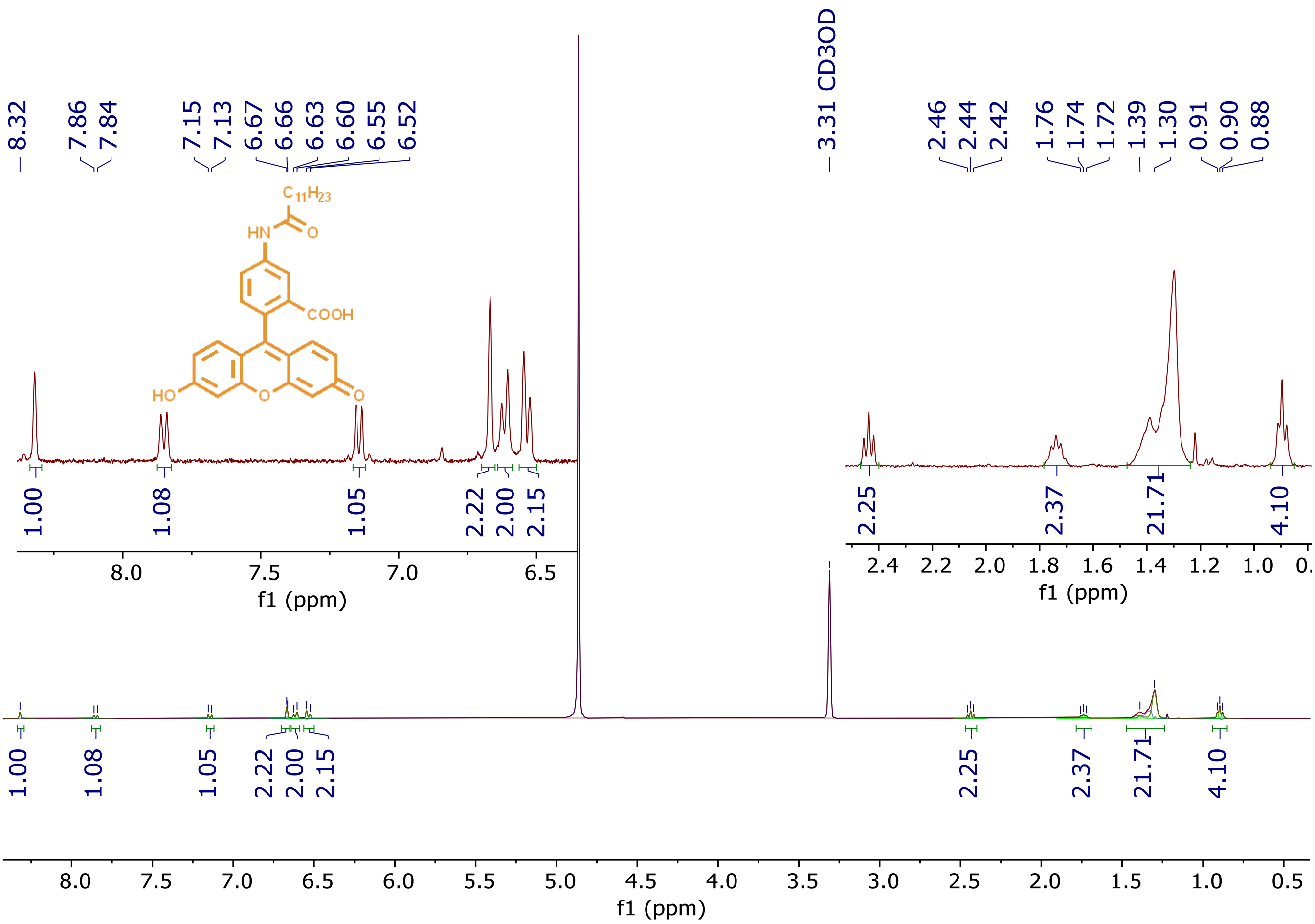

Figure S 8: ^{1}H-NMR of FlC12 in MeOH-*D4.*

^{1}H NMR (400 MHz, MeOH-*d4*, TMS) δ [ppm] = δ 8.32 (s, 1H), 7.85 (d, J = 8.4 Hz, 1H), 7.14 (d, J = 8.4 Hz, 1H), 6.73 – 6.41 (m, 6H), 2.44 (t, J = 7.4 Hz, 2H), 1.74 (t, J = 7.4 Hz, 3H), 1.30 (s, 14H), 0.90 (t, J = 6.5 Hz, 4H).

S2. Singlet oxygen emission quantum yields

The singlet oxygen detection measurements were performed in quartz glass cuvettes (d = 10.0 mm or d = 2 mm). The phosphorescence of $^1O_2$ at around 1270 nm was detected with a Horiba Jobin-Yvon FluoroMax Plus-C automated benchtop spectrofluorometer as described in the Chemicals and Equipment section above. Absorption spectroscopy was measured with a JASCO Spectrometer V-760 and was done before and after each singlet oxygen measurement. Thus, stability of the respective complex on the timescale of the measurements was ensured. For each sample, emission spectra were recorded upon excitation at 480 nm. As a reference the whole procedure was repeated for RuC_0. After baseline correction for each measurement, the area below the signal was integrated. The respective singlet oxygen quantum yield $\phi(^1O_2)$ was calculated and referenced against literature reported value for RuC_0 of 0.73±0.06. [3]

The following equation was used:

$$\Phi_c = \Phi_R \left(\frac{A_R}{I_R}\right)\left(\frac{I_C}{A_C}\right) \qquad \textbf{Eq. S 1}$$

In this equation, Φ_x is the quantum yield and A_x the absorbance of the respective substance (reference (R) RuC_0 or compound (C)) and I_x the integral of the singlet oxygen emission.

Due to short lifetime of $^1O_2$ in water and also in D_2O and deuterated buffer,[4] the $\phi(^1O_2)$ was only measured in the fully deuterated organic solvent MeOH-d_4.

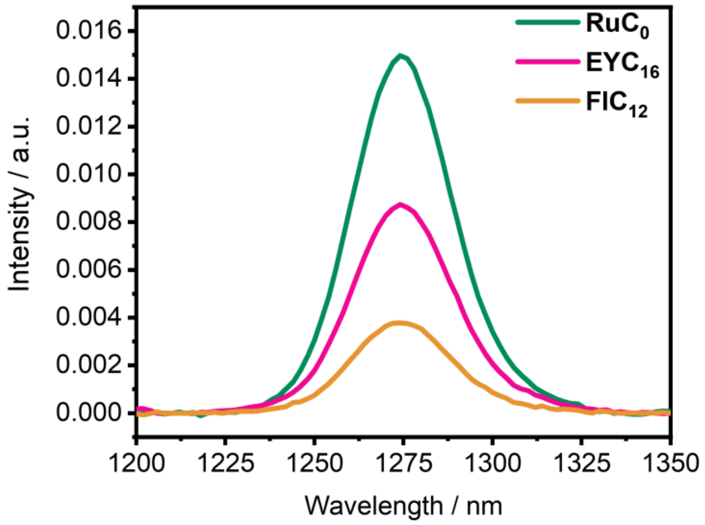

Figure S9: Singlet oxygen emission band of RuC_0, EYC_{16} and FlC_{12} in MeOH-*d₄* in absence of NADH and upon excitation at 480 nm after baseline correction. The absorbance of the chromophores was adjusted to 0.1 at 480 nm in all cases and excited at λ_{ex}=480 nm.

Compound	$\phi(^1O_2)$
EYC_{16}	0.42±0.04
FlC_{12}	0.19±0.02
RuC_0	0.73±0.06

S3. NADH concentration experiment

For this experiment liposome were prepared as described in 5.2 for symmetric liposomes with 100 µl EYC_{16}. This time as much liposomes as possible were collected at each possible step.

Then absorption before and after 60 min of irradiation with the usual setup was measured. The measurement at t=60 was subtracted from t=0. Then with the molar extinction coefficient of NADH of 6300 l mol^{-1} cm^{-1}[5] at 340 nm the concentration of the starting 0.5 ml was calculated.

Then with the area a DPPC lipid takes up (0.7 nm^2)[6] and the number of Lipids used the surface all lipids can make were calculated. With the inner and outer radius of a 4 nm thick membrane[6] the inner and outer surface is calculated of all liposomes. The measured DLS size for each sample was multiplied with 0.9 to accommodate for the Difference in actual size vs. hydrodynamic radius.[7,8] The calculated amount of inner and outer surface divided by total surface gives the total amount of liposomes. With that the total volume of all liposomes can be calculated. Therefore, the final concentration of NADH in the total liposome volume can be calculated.

Table S 1. Calculated concentrations of local concentration of NADH of samples with 1% EYC_{16}.

**Sample**	**Size by DLS / nm**	**Calculated NADH concentration / mM**
Sample 1	159.5	39.7
Sample 2	157.9	43.9
Sample 3	162.9	40.3

The concentration is not 56 mM; however, it was reported that there is significant loss of lipids (30-50 %) during sample preparation[9], explaining the lower detected amount of encapsulated NADH.

S4. Calcein leakage test.

For this experiment liposome were prepared as described in 5.2 without any NADH but with 1 % EYC_{16} 1 % FlC_{12}. Instead of putting 20 mg of NADH in the buffer 21.8 mg (70 mM) Calcein was added. This standard test is used for accessing the permeability of membranes.[10–13]

The DPPC liposomes were examined on Day 0 and Day 1 with and without irradiation.

Irradiation was done by the usual setup. Spectra were recorded from 495-700 nm while exciting a 485 nm. After SEC 10 µl of the solutions were transferred to a cuvette and diluted with 990 µl of our phosphate buffer. After the measurements at t=0, t=10, t=30 and t=60 min, 150 µl of a 1mM Triton X-100 solution was added.

The rest of the solutions was stored in the dark for the next day where the same measurements were done. Triton-X disturbs the membrane and releases the calcein which reduces the concentration so that the self quenching of calcein is not prominent anymore.

The spectra in Figure S 10 are the average of three samples each.

a) b)

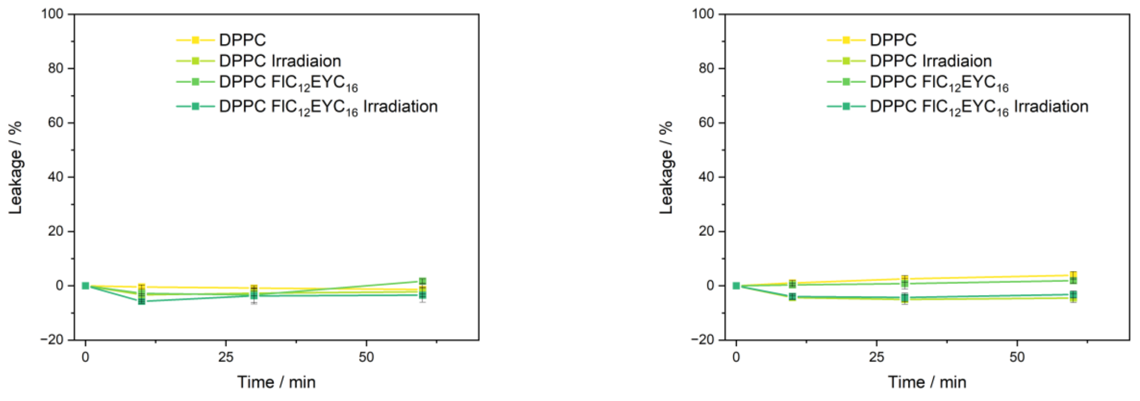

Figure S 10: The emission of calcein is compared before and after addition of Triton X-100.The change of this emission is the leakage. Emission maxima at 518 nm were tracked. The samples are DPPC samples with and without EYC_{16}, FlC_{12} and with or without irradiation. a) Day 0 b) Day 1

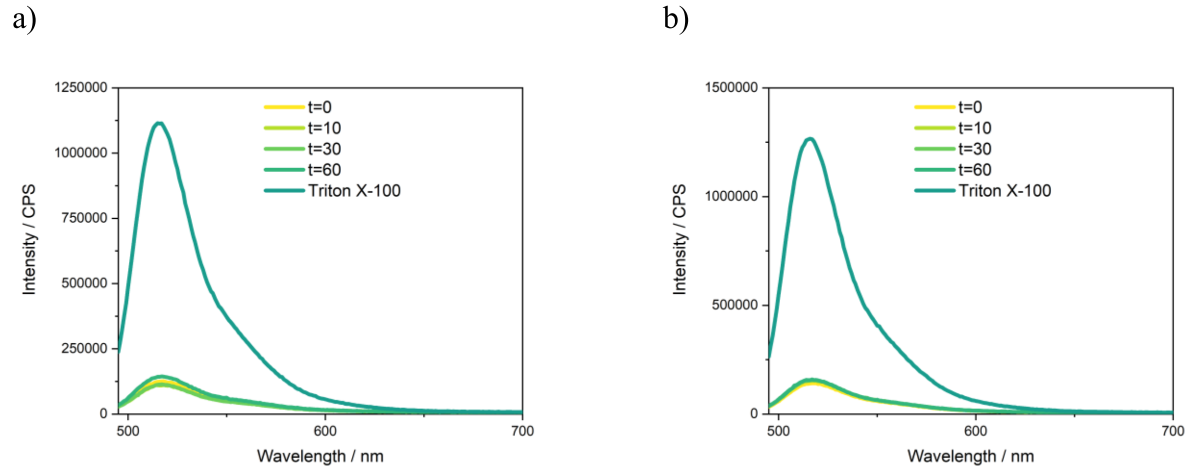

Figure S 11: Emission spectra of calcein in DPPC liposomes with 1% FlC$_{12}$ and 1% EYC$_{16}$ low intensity (self-quenching concentration) and high intensity upon addition of Triton X-100 to disrupt the lipid membrane. a) Day 0 b) Day 1

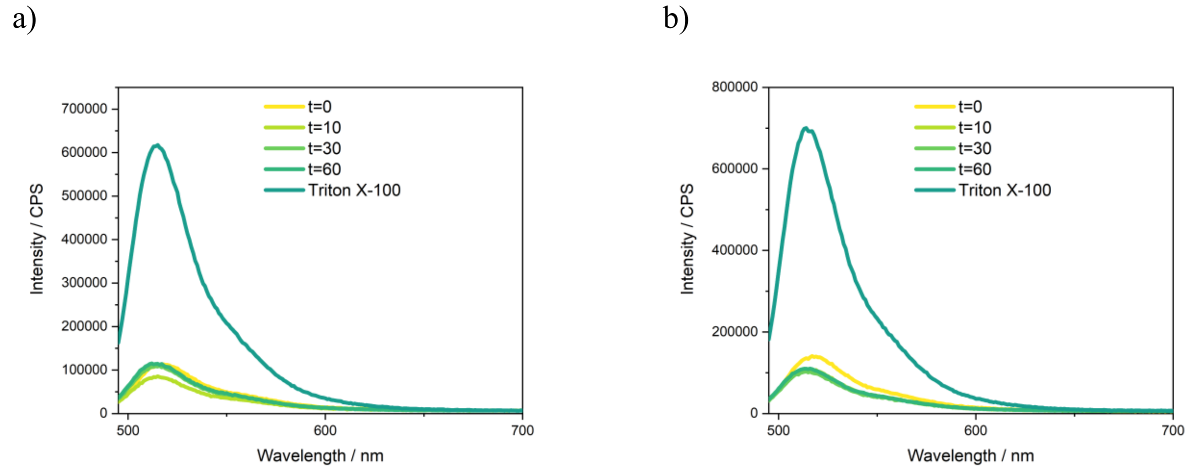

Figure S 12: Emission spectra of calcein in DPPC liposomes with 1% FlC$_{12}$ and 1% EYC$_{16}$ while irradiation with the usual setup at low intensity (self-quenching concentration) and high intensity upon addition of Triton X-100 to disrupt the lipid membrane. a) Day 0 b) Day 1

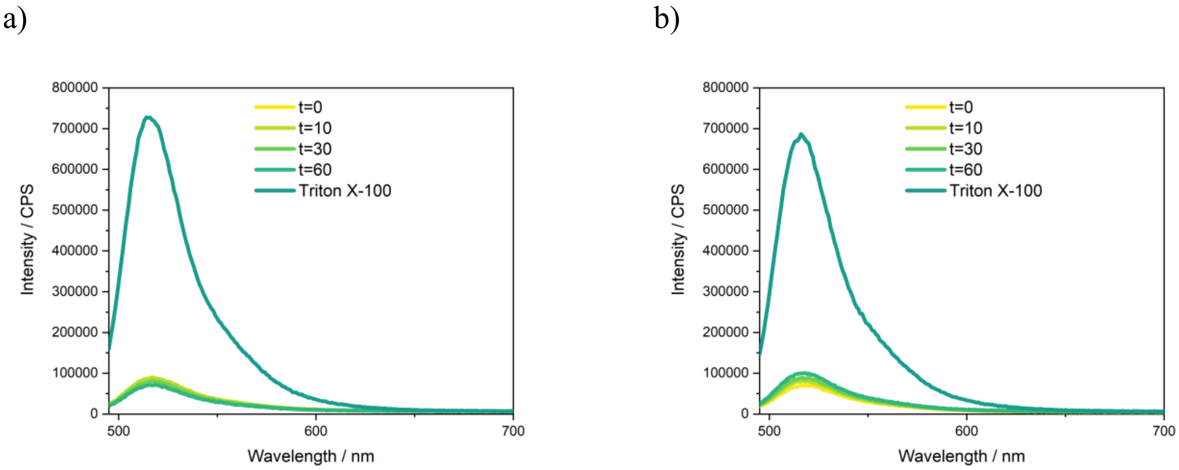

Figure S 13: Emission spectra of calcein in DPPC liposome at low intensity (self-quenching concentration) and high intensity upon addition of Triton X-100 to disrupt the lipid membrane. a) Day 0 b) Day 1

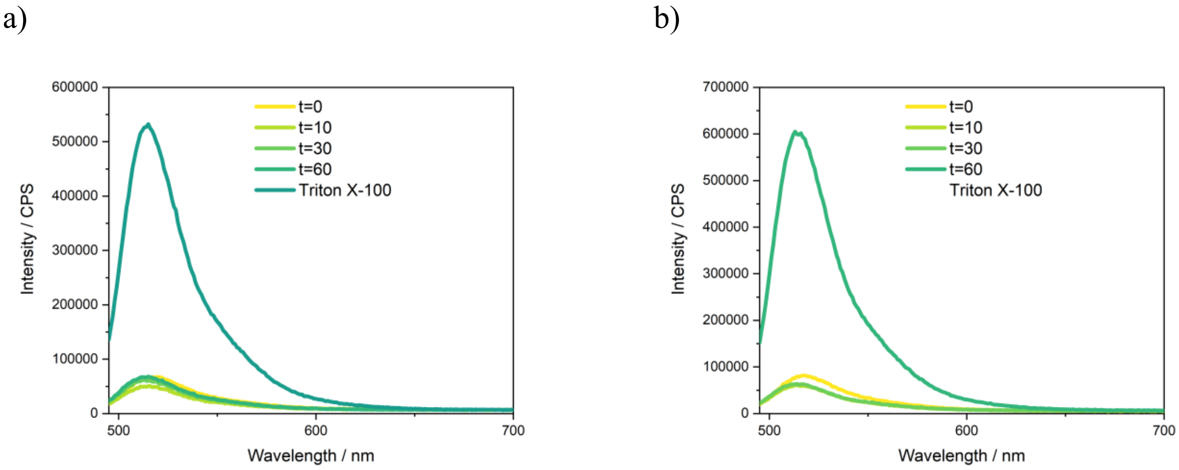

Figure S 14: Emission spectra of calcein in DPPC liposomes while irradiation with the usual setup at low intensity (self-quenching concentration) and high intensity upon addition of Triton X-100 to disrupt the lipid membrane. a) Day 0 b) Day 1

S5. Sample preparation

5.1 Preparation of the Phosphate Buffer

1000 mM phosphate buffer was prepared by adding 21.92 g potassium dihydrogen phosphate (12.6 mmol) 275 g dipotassium hydrogen phosphate (987 mmol) in 1 L Milli-Q water. Potassium hydroxide was added to adjust the pH to 9.01.

A 100 mM buffer (pH 8.99) was created by taking 100 mL of the 1M buffer and dilute it with 900 mL Milli-Q water.

5.2 Liposome preparation

Symmetric Liposomes

A mixture of 1 ml of a 5 mM DPPC stock solution in chloroform, 1 ml of a 0.05 mM 14:0 PEG-2000 PE stock solution in chloroform and the appropriate amount (**X** µl) of a 0.5 mM EYC_{16} in methanol and (**Y** µl) of FlC_{12} were combined in a round bottle flask and dried first by using the rotary evaporator followed by 0.05 mbar oil pump vacuum for at least eight hours. To the residue 0.5 ml of 56 mM NADH in phosphate buffer was added. Three freeze-thaw cycles were performed with the mixture by using liquid nitrogen and a 55°C water bath. Afterwards, the liposomes were extruded 11 times with a membrane filter with 0.2 µm pore size. A G25 Sephadex column (6 cm length, 2 cm diameter) with phosphate buffer as mobile phase was used to exchange the surrounding solvent of the liposomes. Now, 56 mM of NADH is encapsulated inside of liposomes. In all cases, the first fraction containing the liposomes was collected and diluted to 3 ml with phosphate buffer.

The values for X and Y are reported along with the measurement results in the Table S4.

Asymmetric Liposomes

A mixture of 1 ml of a 5 mM DPPC stock solution in chloroform, 1 ml of a 0.05 mM 14:0 PEG-2000 PE stock solution in chloroform and the appropriate amount (X µl) of a 0.5 mM EYC_{16} in methanol were combined in a round bottle flask and dried first by using the rotary evaporator followed by 0.05 mbar oil pump vacuum for at least three hours. In the dark, to the residue 0.5 ml phosphate buffer was added,

as well as 20 mg NADH. Three freeze-thaw cycles were performed with the mixture by using liquid nitrogen and a 55°C water bath. Afterwards, the liposomes were extruded 11 times with a membrane filter with 0.2 µm pore size. A G25 Sephadex column (4.5 cm length, 2 cm diameter) with phosphate buffer as mobile phase was used to exchange the surrounding solvent of the liposomes. Now, 56 mM of NADH is encapsulated inside of liposomes. In all cases, the first fraction containing the liposomes was collected. Then, the appropriate amount of (Y µl) of FlC_{12} was added and heated for 1 h at 35 °C. A second G25 Sephadex column (3.2 cm length, 2 cm diameter) with phosphate buffer as mobile phase was used to exchange the surrounding solvent again. In all cases, the first fraction containing the liposomes was collected.

The values for **X** and **Y** are reported along with the measurement results in the Table S5.

S6. Exemplary dynamic light scattering (DLS)

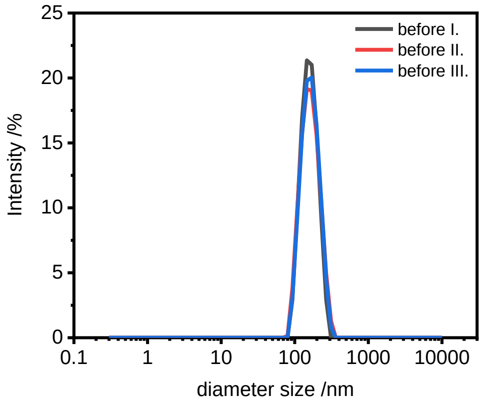

Figure S15: DLS measurement of Nr.40 before the photosensitized conversion

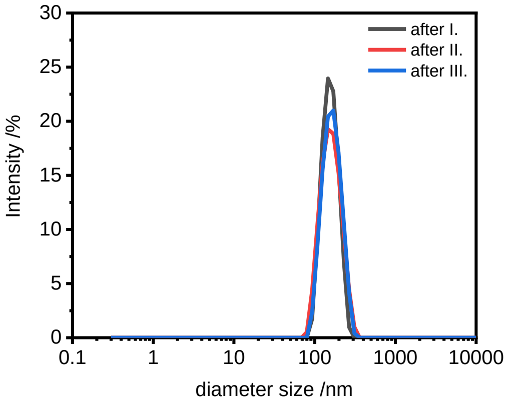

Figure S16: DLS measurement of Nr.40 after the photosensitized conversion

Table S2: DLS Data; average size, polydispersity index and peak one mean, before and after photosensitized conversion.

	before		after	
	Mean	Standard Deviation	Mean	Standard Deviation
Z-Average /nm	154.4	1.472	153.2	2.724
Polydispersity Index PI	0.0511	0.01601	0.04483	0.02949
Peak One Mean by Intensity /nm	165	2.673	163	4.238

S7. Cryo-electron microscopy (Cryo-EM) sample preparation and imaging

Liposomes were prepared as described in 5.2 for symmetric and asymmetric liposomes without NADH and 100 µl EYC_{16} and 100 µl FlC_{12}.

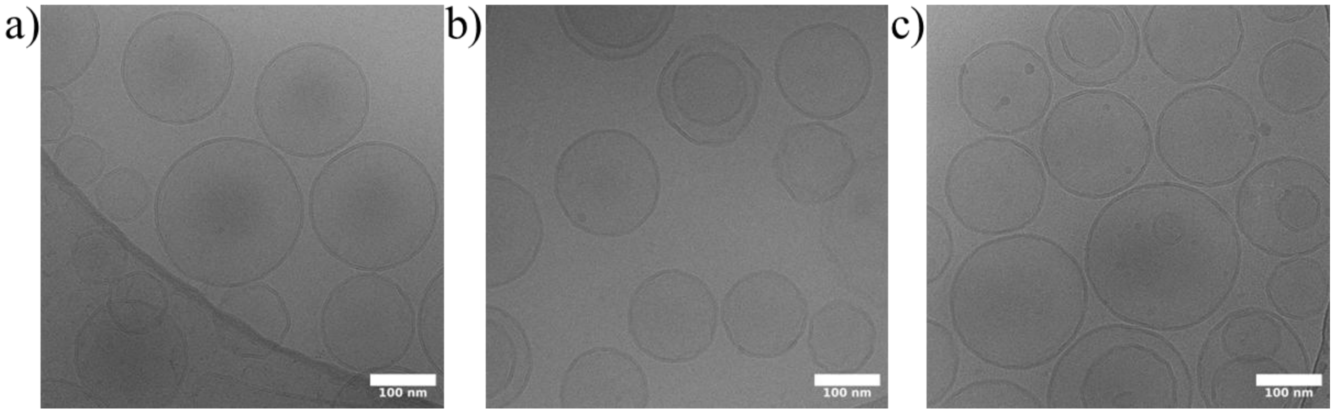

Figure S 17: Cryo EM pictures. a) symmetric DPPC liposomes without any dye. b) symmetric $EYC_{16}FlC_{12}$ DPPC liposomes c) asymmetric $EYC_{16}FlC_{12}$ DPPC liposomes.

S8. Phasor-fluorescence lifetime imaging microscopy

Vesicles are prepared by following:

A mixture of 1 ml of a 5 mM DPPC stock solution in chloroform, 1 ml of a 0.05 mM 14:0 PEG-2000 PE stock solution in chloroform and the appropriate amount (**X** µl) of a 0.5 mM EYC_{16} in methanol and (**Y** µl) of FlC_{12} were combined in a round bottle flask and dried first by using the rotary evaporator followed by 0.05 mbar oil pump vacuum for at least eight hours. In the dark, to the residue 0.5 ml phosphate buffer was added. Three freeze-thaw cycles were performed with the mixture by using liquid nitrogen and a 55°C water bath.

The values for **X** and **Y** are reported along with the measurement results in Table S4.

Measurement:

Each sample is dropped (5 µL) onto standard microscopy onto standard microscopy glass slides and imaged at 512 x 512 pixels at 100 Hz. Imaging for FlC_{12} (Ex. 491 nm, Em. 500 – 530 nm) and for EYC_{16} (Ex. 561 nm, Em. 570 – 700 nm) is accomplished using an 80 MHz pulse laser. Emitted photons are collected and counted using FALCON-time correlated single photon counting. Each pixel is transformed into a phasor plot according to Eq. S 2 and Eq. S 3.[14]

$$g_{i,j}(\omega) = \frac{\int_0^T I(t)\cos(n\omega t)dt}{\int_0^T I(t)dt} \qquad \textbf{Eq. S 2}$$

$$s(\omega) = \frac{\int_0^T I(t)\sin(n\omega t)dt}{\int_0^T I(t)dt} \qquad \textbf{Eq. S 3}$$

in which $g_{i,j}(\omega)$ and $s_{i,j}(\omega)$ are the *x* and *y* coordinates of the phasor plot, n and ω are the harmonic frequency and the angular frequency of excitation, respectively, and T is the repeat frequency of the

acquisition. Frequency domain data acquisition from each pixel can be converted to phasor points using the transformation Eq. S 4 and Eq. S 5.

$$g_{i,j}(\omega) = m_{i,j}\cos(\phi_{i,j})$$ **Eq. S 4**

$$s_{i,j}(\omega) = m_{i,j}\sin(\phi_{i,j})$$ **Eq. S 5**

in which $m_{i,j}$ and $\phi_{i,j}$ are the modulation and phase shift, respectively, of the frequency domain measurement at pixel *i,j*. The decay from each pixel can hence be translated to a point in the phasor plot. Phasor components were identified and separated using LAS X software.

S9. Energy transfer efficiency E_{FRET}

The energy transfer efficiency E_{FRET} was determined by measuring the emission intensity at 522 nm, when excited at 497 nm for samples A-K before illumination with LEDs or the simulated solar light. For that Eq. S 6 was used.[15] In this formula F_{DA} is the luminescence of the donor in presence of the acceptor while F_D is the luminescence of the donor. The symmetric energy transfer rate was averaged over the samples A-F and the asymmetric over the samples H-N.

$$E_{FRET} = 1 - \left(\frac{F_{DA}}{F_D}\right) \cdot 100 \quad \textbf{Eq. S 6}$$

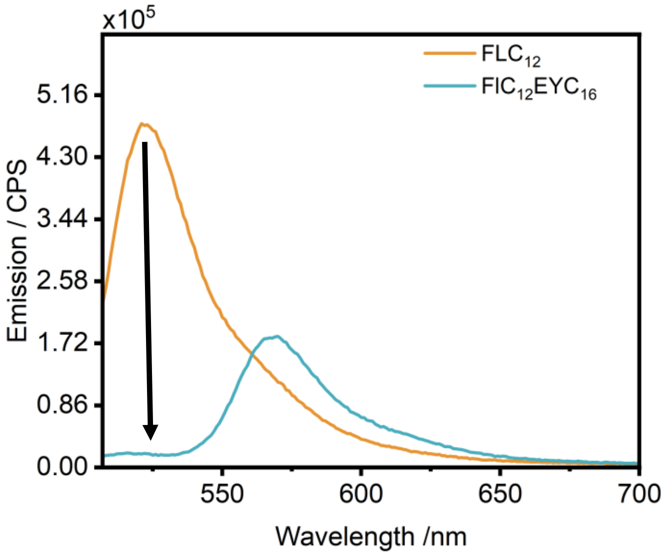

Figure S18: Exemplary quenching data of the FlC_{12} emission in a symmetric liposome (sample set B) where EYC_{16} and FlC_{12} are present.

S10. MD simulations and computation of the FRET rate

Molecular dynamics protocol

We performed two sets of molecular dynamics (MD) simulations, one for FlC_{12} and one for EYC_{16}, each embedded in a DPPC lipid bilayer membrane. The initial structure for the membrane was constructed using the web-based interface CHARMM-GUI.[16,17] 102 DPPC molecules were assembled in each leaflet, which corresponds to an 80 Å by 80 Å membrane slab. The chromophores were initially placed outside of the membrane. Both were modelled as the dianionic tautomers throughout. The full system was placed into an 80 Å by 80 Å by 98 Å rectangular box. The box was filled with water molecules, and the negative charges of the chromophores were neutralized by adding two (FlC_{12}) or one (EYC_{16}) potassium counter ions.

All simulations were performed with the program packages Amber22/Ambertools23.[18] The chromophores were described using the General Amber Force Field (GAFF2)[19,20] with restricted electrostatic potential charges computed with antechamber (part of Ambertools23) using an electrostatic potential computed with B3LYP[21–23]-D3BJ[24]/def2-SVP[25,26] in Gaussian 16, revision C.01.[27] The DPPC molecules were described using the LIPID21 force field[28], and ions and water were modelled using the “optimal point charge” (OPC) water model[29]. Prior to simulation, the systems were minimized in 10,000 minimization steps. For the first 5,000 steps, a steepest descent algorithm was employed, which was switched to a conjugate gradient algorithm for the second 5,000 steps. For the propagation in time, a time step of 2 fs was used. To enable this large time step, the SHAKE algorithm[30] was enabled to freeze hydrogen bonds at a relative geometric tolerance of $1*10^{-7}$. Periodic boundary conditions were superimposed under constant pressure conditions with anisotropic pressure scaling with a Berendsen barostat. The cutoff for non-bonded interaction terms was set to 10 Å. Following minimization, the systems were heated from 0 to 300 K in 50,000 steps (100 ps) and kept at 300 K for all subsequent simulations using a Langevin thermostat.

Both systems were equilibrated with the chromophores in the aqueous phase until they diffused close to the membrane surface, which was 226 ns (FlC_{12}) or 135 ns (EYC_{16}). Then, the chromophores were pulled into the membrane by applying a harmonic potential of 5 kcal/mol/Å^2 on the distance between the centre of mass of the central three six-membered ring of the chromophores and the centre of the DPPC membrane. By reducing the equilibrium distance of the harmonic potential by 0.5 Å every 200 ps to 0 Å, the chromophores were forced into the membrane. Once they were embedded in the

membrane, each system was propagated without constraints for 500 ns. The resulting density distributions are presented in the manuscript, Figure 4 a, b. Structural data from the last 200 ns of these simulations were used for computing the FRET rates (see below).

To estimate the free energy profile along the membrane cross section for both chromophores, we performed two sets of umbrella sampling simulations[31]. In umbrella sampling, a series of simulations is run with harmonic constraints applied along a collective variable, which in our case is the distance between the chromophore and the centre of the membrane. By recording the probability distribution within each constraint window along the collective variable, the underlying free energy profile can be constructed. Starting from the effective distance of the final frames in the unconstrained simulations, we pulled the chromophores in or pushed them out of the membrane by reducing or increasing the constraint variable in steps of 0.5 Å every 100 ps, generating 91 windows from 0 to 45 Å in steps of 0.5 Å for both chromophores. Again, a force constant of 5 kcal/mol/Å^2 was used. The systems were equilibrated for 1 ns in each window, and the probability distribution was recorded subsequently in each window for 10 ns, during which 100,000 frames (one every 100 fs) were stored for the analysis. The free energy profiles presented in the manuscript, Figure 4, were constructed using the weighted histogram analysis method[32] implemented in the WHAM code by Grossfield.[33]

Energy transfer computation

According to FRET theory[34–36], the energy transfer rate k_{FRET} can be directly computed from structural and photophysical data:

$$k_{\mathrm{FRET}} = \frac{1}{\hbar^2 c(4\pi\varepsilon_0\eta^2)^2}\frac{\kappa^2|\vec{\mu}_{\mathrm{D}}|^2|\vec{\mu}_{\mathrm{A}}|^2}{r^6}J. \quad \textbf{Eq. S 7}$$

Here, $\hbar$ is the reduced Panck constant, c is the speed of light, ε_0 is the vacuum permittivity, η is the medium refractive index (1.478 in DPPC[37]), $\vec{\mu}_{\mathrm{D}}$ and $\vec{\mu}_{\mathrm{A}}$ are donor and acceptor transition dipole moments (TDMs), respectively, r is the distance between the chromophores, J is the spectral overlap between the donor emission and acceptor absorption spectra, and κ^2 is the orientation factor. This orientation factor depends on the alignment between the two transition dipole moments:

$$\kappa^2 = (\cos\theta_{AD} - 3\cos\theta_{Ar}\cos\theta_{Dr})^2, \quad \textbf{Eq. S 8}$$

Where θ_{AD} is the angle included between the TDMs, and θ_{Ar} and θ_{Dr} are the angles included between the distance vector and the acceptor or donor TDM, respectively.

We computed the spectral overlap from the experimentally recorded spectra; each normalized to the unit area. The resulting overlap is $1.05*10^{-2}$ cm. We computed the TDM magnitudes for fluorescein and eosin Y in their native, unsubstituted dianionic forms using CAM-B3LYP[38]-D3BJ/cc-pVDZ[39,40] in Gaussian 16. Here, we included solvent effects for water implicitly using a conductor-like polarizable continuum model by placing the solute in a self-consistent reaction field. To compute the TDM between the lowest electronic excited state and the ground state, we optimized fluorescein in the excited state (which corresponds to the emission TDM), and eosin Y in the ground state (which corresponds to the absorption TDM). The TDM strengths are 18.24 atomic units for fluorescein, and 15.75 atomic units for eosin Y.

For computing the distance and the alignment factor κ^2, we exploit the fact that the TDMs are perfectly aligned with the phenoxy-phenoxy vector in the equilibrium structure of both chromophores (<0.5° deviation), such that we approximate the TDM orientation in each MD simulation frame with this phenoxy-phenoxy vector. We computed the FRET rates from the last 200 ns of the unconstrained trajectories, from which 200 equidistant frames (every 1 ns) were taken for each chromophore. Assuming that both chromophores evolve independently in the membrane, we combined the structural data of each FlC_{12} frame with every EYC_{16} frame, resulting in 40,000 unique combinations. The z-position of both chromophores, i.e., the distance to the membrane centre, is constrained by the topology of the membrane, and thus directly taken from the simulations. The distance in z-direction is then computed as the sum of the z-positions in the case of both chromophores in opposite membrane leaflets, of as the difference, when they are placed in the same leaflet. However, since the membrane is a two-dimensional construct, the chromophores can diffuse along the membrane plane, which significantly affects the relative distance and orientation of the chromophores. In other words, for each frame, the z-position of the chromophores is defined from the simulation, but the x- and y-coordinates are ambiguous. Thus, we compute these from a model which includes the surface loading of EYC_{16}. For each individual energy transfer, the relative position of EYC_{16} with respect to the position of FlC_{12} determines the energy transfer rate. Thus, we place FlC_{12} at the centre of a model hexagon. The area of this hexagon is equal to the mean molecular area each EYC_{16} has. In the experiment, the EYC_{16}:DPPC ratio is 100:1, and since every DPPC has an average membrane surface area of roughly 70 Å^2, every EYC_{16} occupies a hexagon of roughly 7,000 Å^2. With FlC_{12} in the centre of the hexagon, we scan all possible x- and y-coordinates for EYC_{16} within this hexagon on 10 circles or equidistant radii with an equal point-per-circumference ratio. With the so-computed distance between FlC_{12} and EYC_{16} in x- and y-direction and

the z-component as computed above, the distance vector between the chromophores can be constructed. To implicitly account for the collision radius of both chromophores, we neglected all pairs of frames with distances below 10 Å.
To compute the alignment of the two chromophores, we additionally need to include the free rotation around the z-axis, which is again a consequence of the two-dimensional nature of the DPPC bilayer. Thus, at every point of the hexagon, we rotated EYC_{16} around the z-axis in steps of 30° from 0° to 330°. In total, we combined each of the 200 snapshots of FlC_{12} with 200 snapshots of EYC_{16}, which we scanned over 111 symmetry-unique grid points of the hexagon, and EYC_{16} was rotated 12 times at each of these grid points. In the case of opposite leaflets for FlC_{12} and EYC_{16}, this results in the computation of roughly 53 million individual energy transfers, while in the case of the same leaflet roughly 3 million data points are rejected due to the 10 Å distance cutoff, resulting in about 50 million individual energy transfer rates. In both cases, the average orientation factor κ^2 is roughly 0.75, which hints at slightly more favorable alignments compared to completely random alignments (κ^2 of 2/3=0.6666…). However, the distance between the chromophores in the same leaflet is significantly smaller than that in opposite leaflets, which results in the large difference in the average energy transfer rates of 15 ps^{-1} in same leaflets, and 90 ns^{-1} in opposite leaflets.

S11. Stern-Volmer quenching

General principle

In a Stern-Volmer Experiment we prepared a series of liposomes with 1 % FlC_{12} and differing amounts of EYC_{16} (0, 0.3, 0.6, 1, 3 eq.).

Liposomes preparation

A mixture of 1 ml of a 5 mM DPPC stock solution in chloroform, 1 ml of a 0.05 mM 14:0 PEG-2000 PE stock solution in chloroform, 100 µl of a 0.5 mM FlC_{12} solution in methanol and (**0, 30, 60, 100 or 300** µl) of a 0.5 mM EYC_{16} solution in methanol were combined in a round bottle flask and dried first by using the rotary evaporator followed by 0.05 mbar oil pump vacuum for at least eight hours. To the residue 0.5 ml phosphate buffer was added. Three freeze-thaw cycles were performed with the mixture by using liquid nitrogen and a 55°C water bath. Afterwards, the liposomes were extruded 11 times with a membrane filter with 0.2 µm pore size. A G25 Sephadex column (6 cm length, 2 cm diameter) with phosphate buffer as mobile phase was used to exchange the surrounding solvent of the liposomes and then diluted to 3 ml.

Lifetimes were measured by exciting with 451 nm and measuring with a long pass filter of 495 nm until 930 nm. In this measuring window EYC_{16} and FLC_{12} emission are present and overlap. Therefore, we cannot separate the lifetime signal from FLC_{12} and EYC_{16} and only a weighted lifetime of a biexponential fit can be gained. As the self-quenching of EYC_{16} leads to shorter lifetimes EYC_{16} lifetime is not the same value for all different experiments and therefore the lifetime of FLC_{12} cannot be separated.

Emission data

Table S 3: Obtained Data from fitting of the kinetic traces of 1% FlC_{12} in DPPC Liposomes and varying amounts of EYC_{16}.The lifetimes τ_1/τ_2 obtained from the biexponential fit were calculated $\boldsymbol{\tau = \tau_1 a_1 + \tau_2 a_2}$ for the average weighted lifetime where a_1 and a_2 are the relative amplitudes.

**Eq. EYC_{16}**	**τ_1 (a_1)/ ns**	**τ_2 (a_2) /ns**	**τ /ns**
0	3.36 (100)*	-	3.36
0.3	0.997 (16.64)	3.18 (83.36)	2.81
0.6	0.87 (24.66)	2.70 (75.34)	2.25
1	0.61 (39.11)	2.16 (60.89)	1.55

3	0.079 (95.08)	0.48 (4.92)	0.099

* τ_0 of 1% FlC_{12} in DPPC liposomes.

In Figure 2d in the main manuscript, the lifetime τ_0 of 2% FlC_{12} in DPPC vesicles is only 1.1 ns, which is likely due to self-quenching of FlC_{12} at higher local concentration in the membrane.

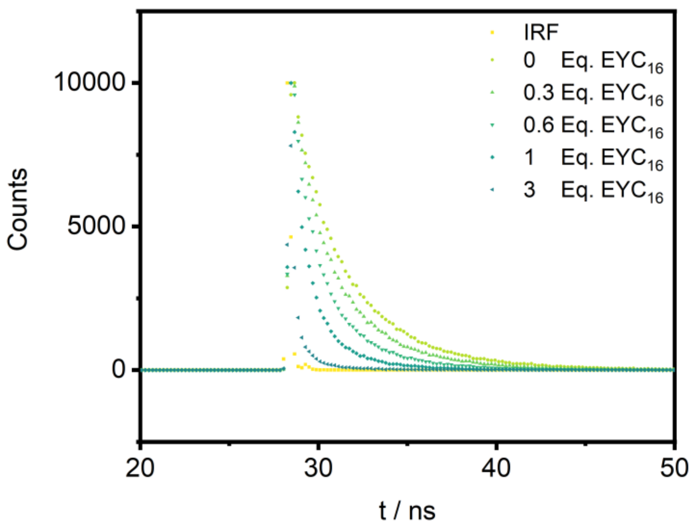

Figure S 19: Kinetic traces of luminescence decay of DPPC liposomes with 1% FLC_{12} and 0, 0.3, 0.6, 1 and 3 eq. of EYC_{16} in the membrane. λ_{exc}= 451 nm.

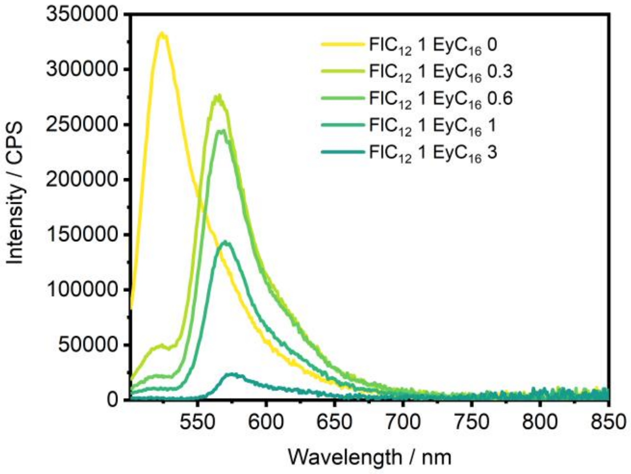

Figure S 20: Emission intensity of DPPC liposomes with 1% FlC_{12} and 0, 0.3, 0.6, 1 and 3 eq. of EYC_{16} in the membrane. λ_{exc}= 451 nm.

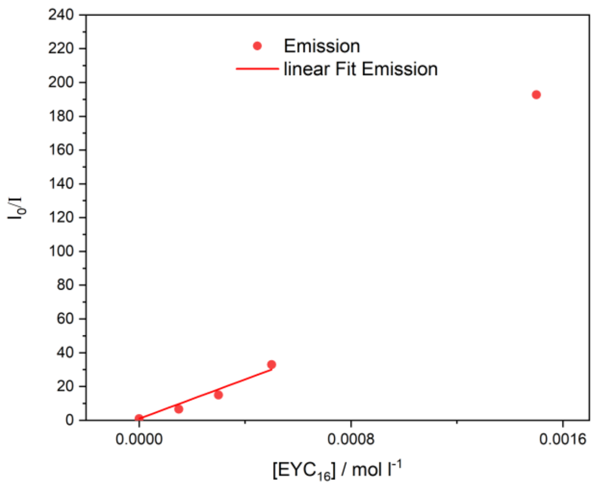

Figure S 21: Stern-Volmer plot based on emission intensities.

$$\frac{I_0}{I} = (1 + K_{sv} \cdot [EYC_{16}])$$ **Eq. S 9**

When staying below 3 eq. EYC_{16} the Stern-Volmer quenching constant K_{SV}= $5.8 \cdot 10^4 \pm 0.5$ M^{-1}, according to Eq. S 9 where I_0 is the emission intensity of FlC_{12} in the absence and I is the emission intensity in the presence of EYC_{16} at various concentrations. After 1 eq. of EYC_{16} in the membrane the linearity of the Stern-Volmer plot is lost, likely due to additional self-quenching effects at high local concentrations by EYC_{16} within the membrane.

$$k_q = \frac{K_{sv}}{\tau_0}$$ **Eq. S 10**

The quenching rate constant k_q was calculated according to Eq. S 10Eq. S 8 with $\tau_0 = 3.4 \pm 0.1$ ns (Table S 3), yielding $k_q = 1.7 \cdot 10^{13} \pm 0.2$ M^{-1} s^{-1}.

S12. Second harmonic generation and fluorescence lifetime imaging

DPPC liposome samples were prepared as usual. To 50 µl DPPC sample 10 µl (1 mol%) FlC_{12} in the same buffer was added and then the SHG signal measured over time.

The Leica-Stellaris setup was used:

Temporally resolved SHG and fluorescence lifetime imaging on FLC12 samples were performed using an inverse microscope (DMi8, Leica Microsystems GmbH, Germany) equipped with a confocal laser scanning microscope (Leica Stellaris 8 CRS Falcon, Leica Microsystems GmbH, Germany). SHG imaging was performed using a tunable ps laser system (picoEmerald, APE GmbH, Germany) based on an optical parametric oscillator (OPO), which is pumped by a Yb fiber laser 1031.1 nm (2 ps pulse width). The OPO signal output can be tuned from 700 to 990 nm. For SHG imaging, the OPO wavelength was set to 830 nm and the laser power was set to 300 mW at the laser output. The samples were placed in 35 mm diameter glass bottom petri dish (ibidi, Germany) using imaging spacers (SecureSeal, Grace Bio Labs, USA) to reduce the sample volume and investigated using a 40x water immersion objective (HC PL IRAPO 40x/1.10 WATER, Leica Microsystems GmbH, Germany) (Figure S 22). The SHG and fluorescence signals were collected in epi-direction via the objective, separated from the excitation laser light by a 850 nm long pass dichroic mirror and a 680 nm short pass filter. SHG and fluorescence signals were split using a dichroic mirror at 560 nm and detected by hybrid detectors. The fluorescence signal was detected using a bandpass filter 650 nm, 40 nm bandwidth (Thorlabs GmbH, Germany). The SHG signal was detected using a 415 nm bandpass filter of 10 nm bandwidth (Semrock, USA) in front of the by hybrid detectors. For imaging the reaction kinetics, the following parameters have been used (5.5.2025, DPPC_40x_300mW_830nm):

- Image size 512x512 pixel, 387.5 µm length
- 800 frames, 5.168s/frame
- Scan speed 100 lines/s
- Pixel dwell time 15.225 µs
- Time resolved detection: 100 ps time bin size, 12.5 ns time window

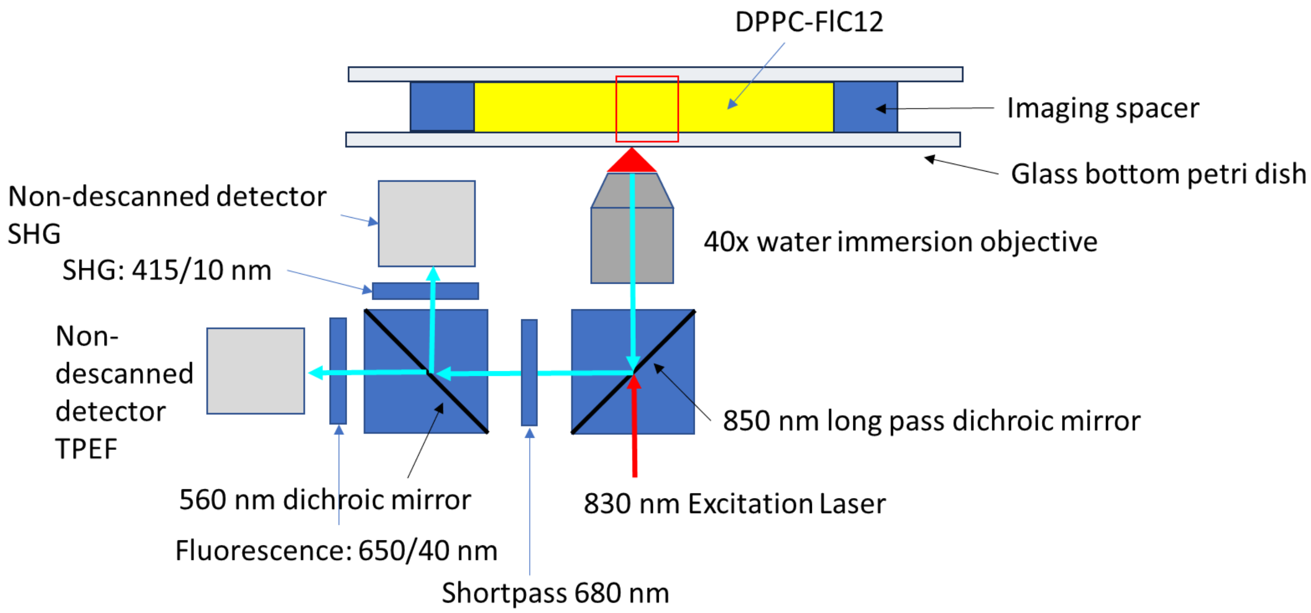

Figure S 22: SHG measuring setup.

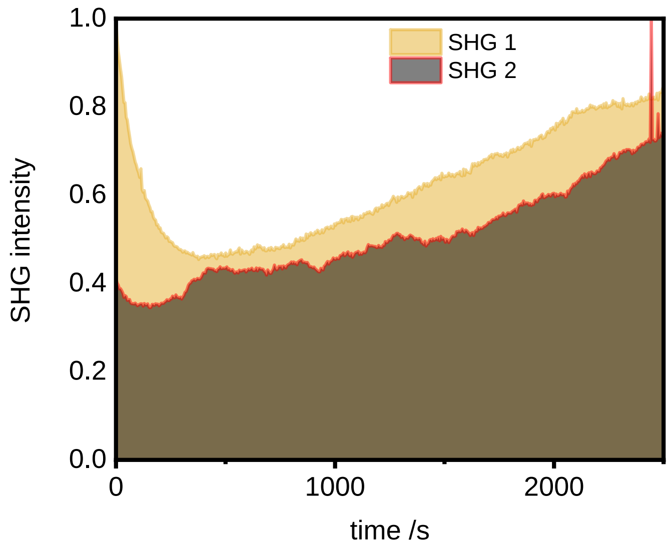

Figure S 23: SHG intensity over time of two extra samples compared to the main manuscript.

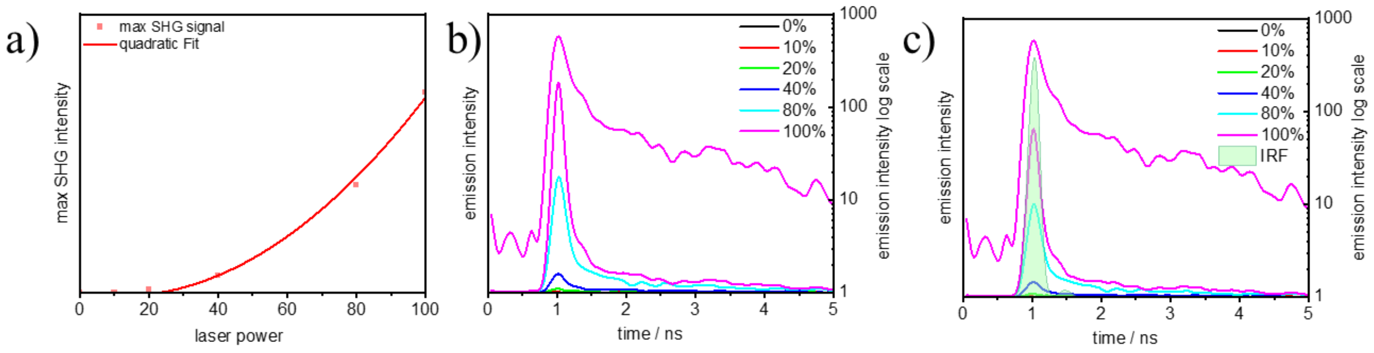

Figure S 24: Proof of SHG. a) Quadratic scaling of the SHG signal to the laser power. b) emission at different laser powers. c) emission at different laser powers with IRF.

Proof of SHG signal by 1) spectral position at half the excitation wavelength, 2) nonlinear signal scaling quadratically with the laser power and 3) short signal of the duration of the excitation laser pulse.
The signal is at half excitation wavelength: here 830 nm excitation, emission is at 415/10 nm.
The quadratic dependence of signal intensity from laser power is clear proof for SHG (Figure S 24a)).
The very short time duration of the signal which is within IRF (instrument response function (temporal)) of the microscope Figure S 24 b,c). In the logarithmic plot there is also a fluorescence contribution, but in linear scale the SHG contribution is evident and can be easily discriminated. By applying two component exponential fitting, the short SHG component can be separated from the fluorescence.
Combining these three facts it is evident that this is indeed SHG.[41,42]

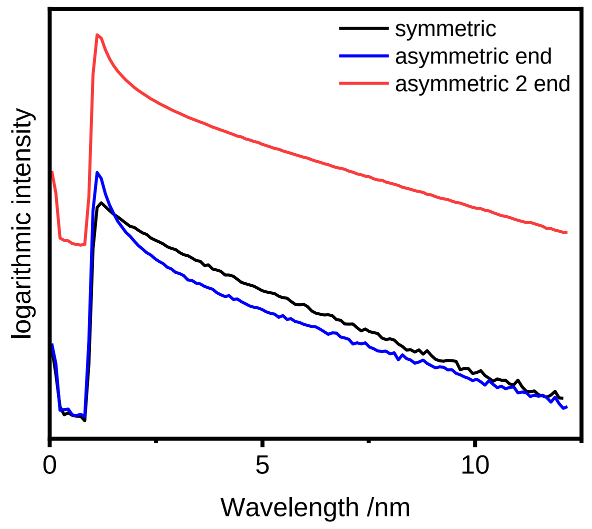

Figure S 25: Fluorescence lifetime imaging: Decay trace of symmetric and asymmetric functionalized nanoreactors. Two photon excited fluorescence excited by 30 mW 797 nm and 100 mW 1031 nm, 10x NA 0.4 objective.

Analysis of the time-resolved fluorescence decay curves revealed distinct decay behaviors depending on the nanoreactor functionalization. Asymmetrically functionalized nanoreactors were best described by a three-exponential decay model, whereas symmetrically functionalized nanoreactors were adequately fitted using a bi-exponential model. In both cases, the shortest lifetime component corresponds to the instrument response function (IRF). This fast component is not perfectly reproduced by the fit due to the limited temporal resolution of the system (100 ps) and the convolution with the IRF and corresponds to the IRF.

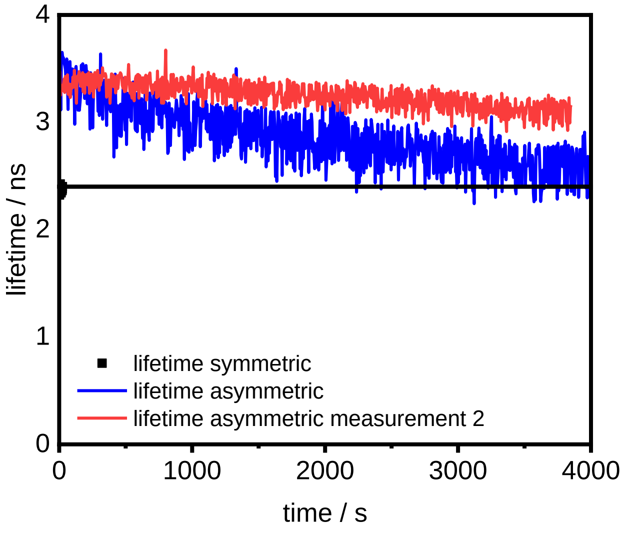

Figure S 26: Fluorescence lifetime imaging: Decay trace of symmetric and asymmetric functionalized nanoreactors. Two photon excited fluorescence excited by 30 mW 797 nm and 100 mW 1031 nm, 10x NA 0.4 objective.

Comparison of the intensity-weighted fluorescence lifetimes shows that symmetrically functionalized nanoreactors exhibit overall shorter lifetimes than asymmetrically functionalized nanoreactors. This trend is consistent with the different dye environments expected for the two architectures. In symmetrically functionalized nanoreactors, fluorophores are present on both sides of the membrane, which can increase the probability of non-radiative decay pathways due to closer proximity between dyes, enhanced self-quenching, or interactions with the membrane interface. In contrast, asymmetric functionalization restricts the fluorophores to a single leaflet, reducing these interactions and resulting in slightly longer intensity-weighted lifetimes. The observed lifetime differences therefore likely reflect variations in the local microenvironment and quenching efficiency of the fluorophores in the two nanoreactor configurations.

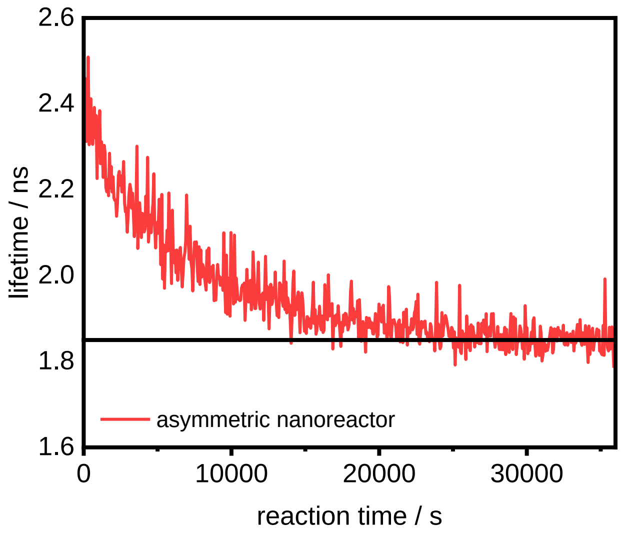

Figure S 27: Time trace of functionalization reaction of asymmetric functionalized nanoreactors measured for 10 hours. Two photon excited fluorescence excited by 30 mW 850 nm and 100 mW 1031 nm, 10x NA 0.4 objective.

The reaction kinetics were monitored by recording the intensity-weighted fluorescence lifetime using FLIM over a period of 10 h. The lifetime trace exhibits a clear exponential decrease during the initial phase of the experiment and reaches a stable plateau after approximately 10 h, indicating completion of the reaction. Fitting the time trace with a single-exponential function yielded a characteristic time constant of $\tau = 7140$ s. Beyond this time scale no further changes in the intensity-weighted lifetime were observed, confirming that the system had reached a steady state and that the system is long term stable.

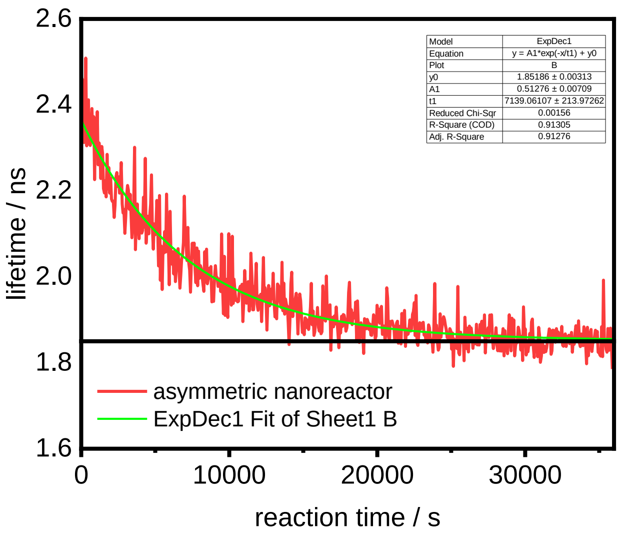

Figure S 28: Time trace of functionalization reaction of asymmetric functionalized nanoreactors measured for 10 hours and single-exponential fitting. Two photon excited fluorescence excited by 30 mW 850 nm and 100 mW 1031 nm, 10x NA 0.4 objective.

After completion of the functionalization reaction, the particles remain stable. This was confirmed at room temperature.

S13. Absorbance and emission at different pH

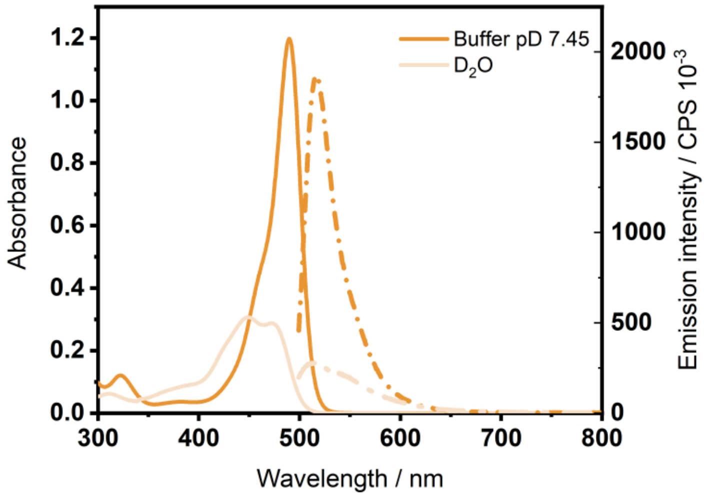

Figure S 29: Absorbance and emission of Fluorescein in D_2O (acidic form of FlC_{12}) and a 47 mM phosphate buffer which was made with K_3PO_4 and DCl and a resulting pD of 7.45 (pH 7.0).[43]

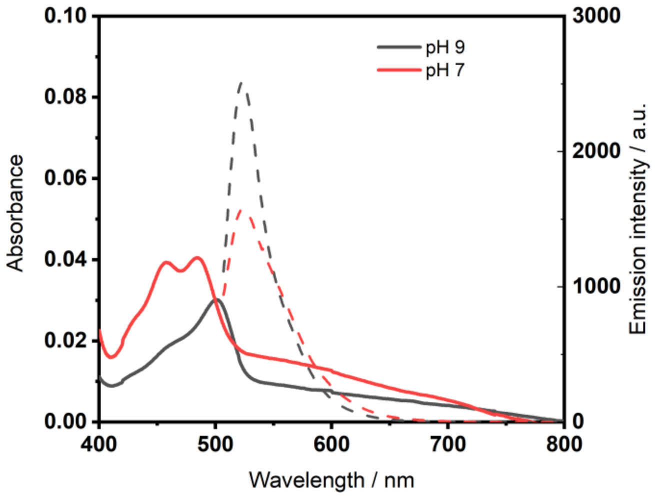

Figure S 30: Baseline corrected absorbance and emission of 0.25 % FlC12 in DPPC liposomes in a 50 mM pH 7.0 Phosphate buffer and a pH 9.0 100 mM Tris(hydroxymethyl)aminomethan Buffer.

S14. Exemplary spectra for NADH conversion

14.1 Symmetric example Nr. 14

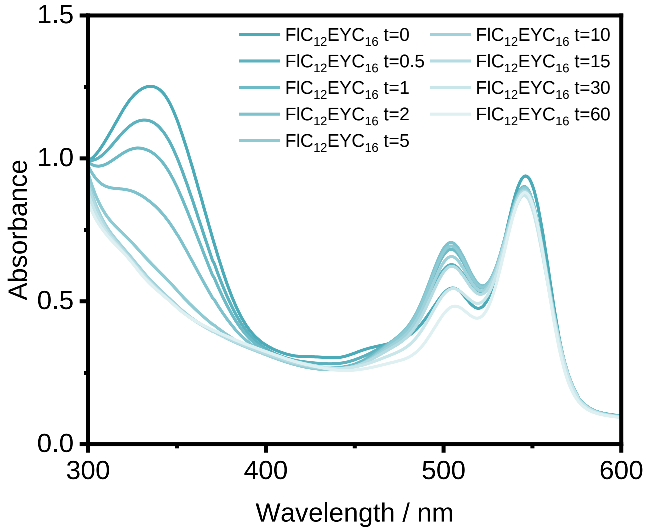

Figure S31: Temporal change in the UV-vis absorption spectra of experiment Nr. 14 without baseline correction.

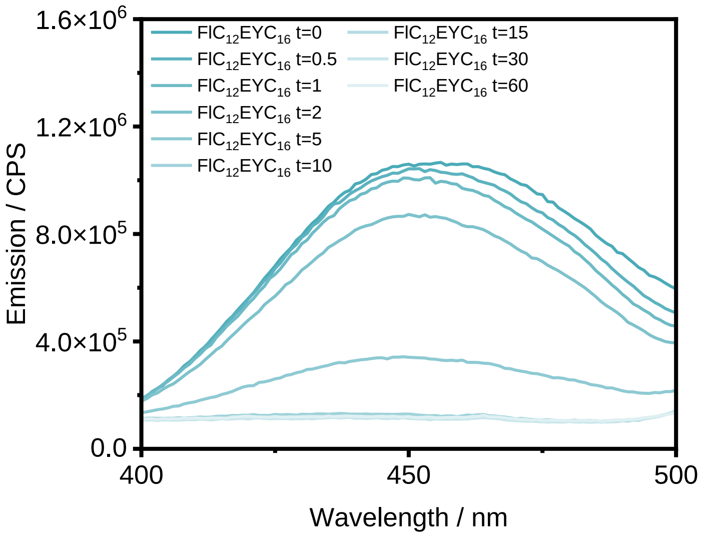

Figure S32: Temporal change in the NADH fluorescence spectra of experiment Nr. 14. λ_{exc} = 340 nm

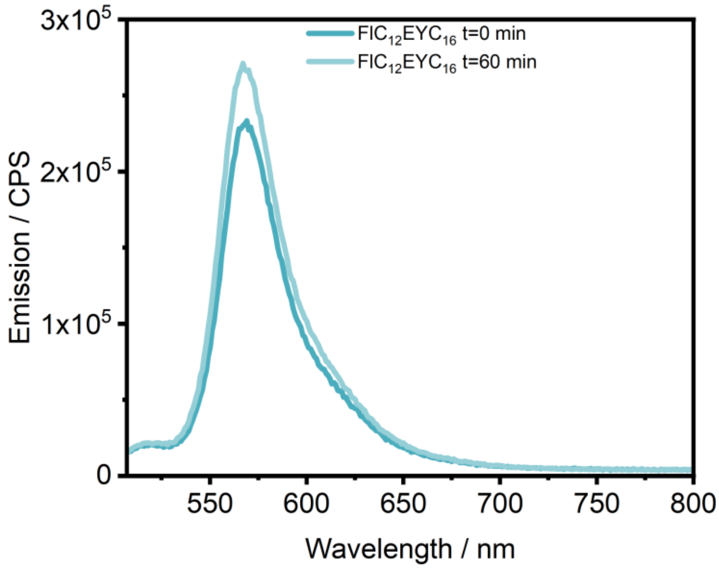

Figure S33: Temporal change in the FLC_{12} and EYC_{16} fluorescence spectra of experiment Nr. 14. λ_{exc} = 497 nm

14.2 Asymmetric example Nr. 30

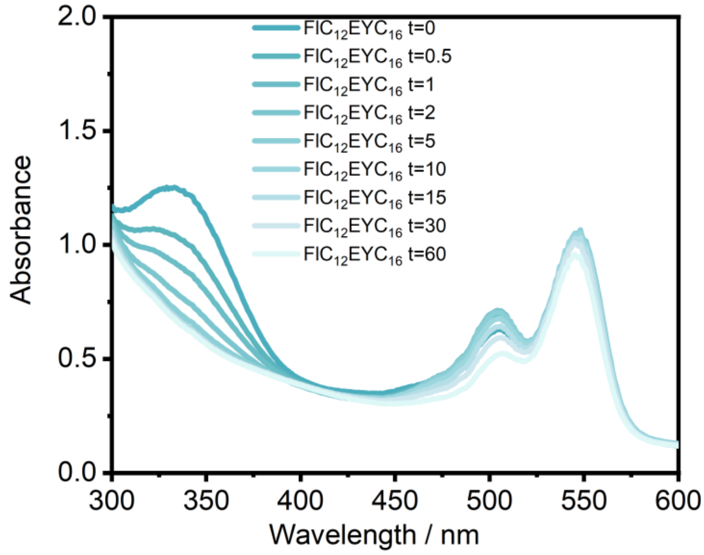

Figure S34: Temporal change in the UV-vis absorption spectra of experiment Nr. 30 without baseline correction.

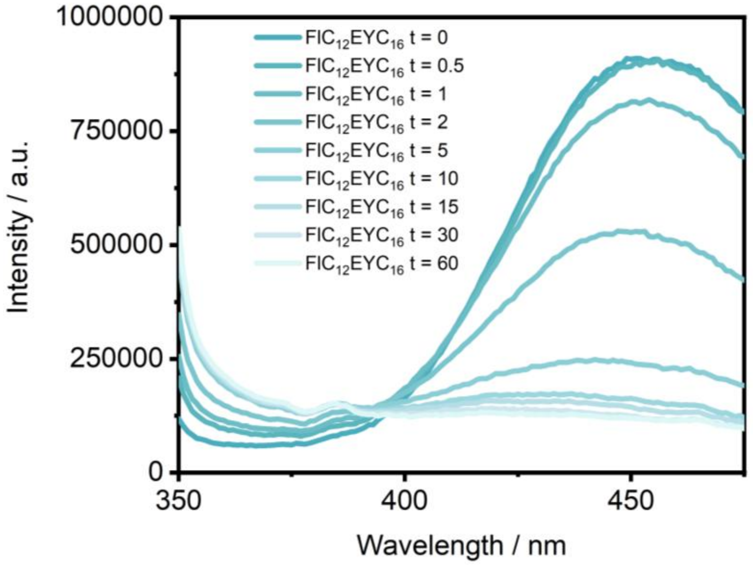

Figure S35: Temporal change in the NADH fluorescence spectra of experiment Nr. 30. λ_{exc} = 340 nm

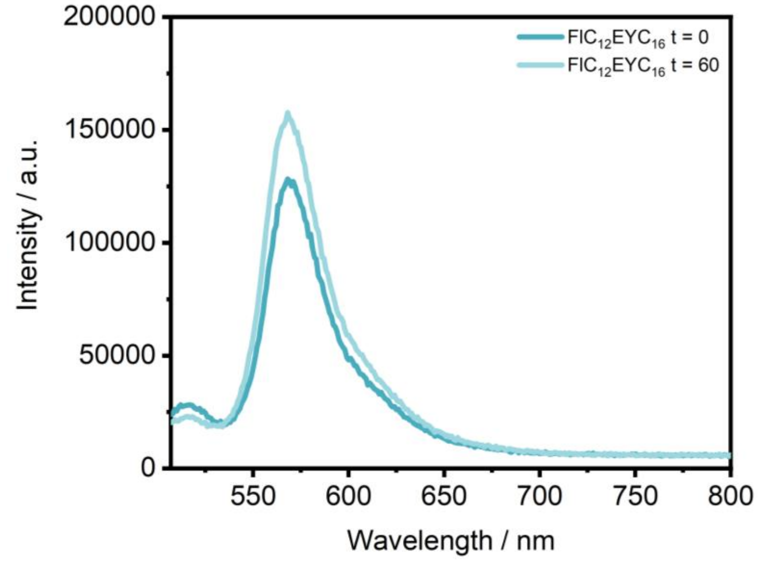

Figure S36: Temporal change in the FLC_{12} and EYC_{16} fluorescence spectra of experiment Nr. 30. λ_{exc} = 497 nm

14.3 Asymmetric example Nr.42

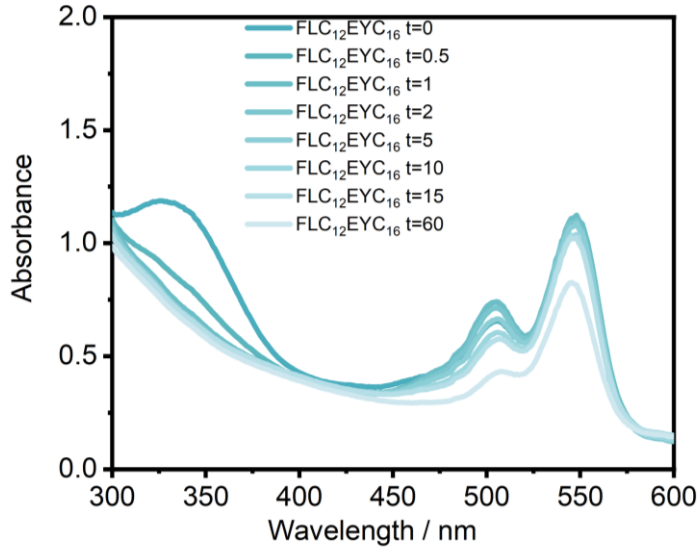

Figure S 37: Temporal change in the UV-vis absorption spectra of experiment Nr. 42 without baseline correction.

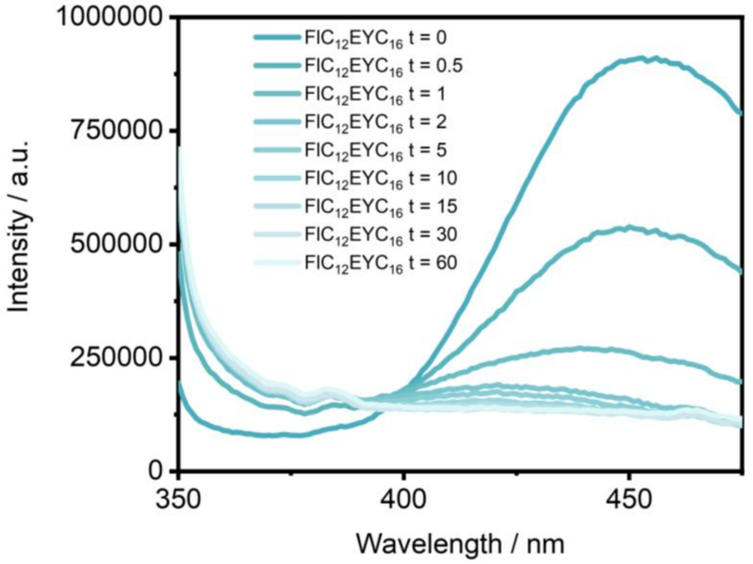

Figure S 38: Temporal change in the NADH fluorescence spectra of experiment Nr. 42. λ_{exc} = 340 nm

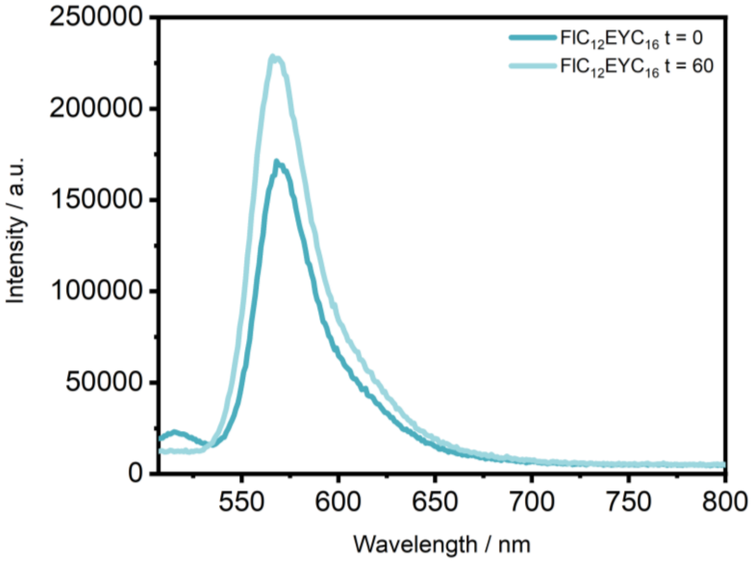

Figure S 39: Temporal change in the FLC$_{12}$ and EYC$_{16}$ fluorescence spectra of experiment Nr. 42. λ_{exc} = 497 nm

S15. Rate constants

If not otherwise noted, all values of the rate constant k were determined by fitting the of the emission intensities over time at 450 nm, or the absorbance at 340 nm with a mono exponential decay fit function (Equation 3).

To obtain the absorbance spectra of FlC_{12} and EYC_{16}, Tyndall scattering needed to be addressed. Due to liposome stability during the experiment, we assumed an unchanged Tyndall scattering and opted to fit the scattering with a simple exponential decay using the respective measurement at t=60 min between the absorbance of NAD+ Fluorescein or Eosin. This new baseline was then subtracted from the t=0 min measurement, leading to a qualitative absorbance spectrum of FlC_{12} / EYC_{16} within the liposomes. The NADH absorbance band at 340 nm was chosen as a parameter, considering variations in quantity and dye loading within each liposome batch, which is related to the true quantity of liposomes.

Tyndall scattering cleaned measurements were standardized to their NADH absorbance and transformed into respective transmission values. This was calculated separately for both dyes.

The Addition of the rate constants was done according Eq. S 11, Eq. S 12 and Eq. S 13, where k_Δ represents the error of the fit.

$$k_{Hyp} = k_{FlC12} + k_{EYC16}$$ **Eq. S 11**

$$k_{Hyp,min} = k_{Fl} - k_{\Delta Fl} + k_{Eo} - k_{\Delta Eo}$$ **Eq. S 12**

$$k_{Hyp,max} = k_{Fl} + k_{\Delta Fl} + k_{Eo} + k_{\Delta Eo}$$ **Eq. S 13**

15.1 Symmetric Liposomes

Table S4: Symmetric liposome data. Amount of EYC_{16} und FlC_{12} as well as k values.

Sample set	Entrance	Fit k /min^{-1}	**X** µL (0.5 mM) EYC_{16}	**Y** µL (0.5 mM) FlC_{12}	Light source
A	1	0.109 ± 0.005	0	100	520 and 465 nm LED
	2	0.80 ± 0.02	100	100	
	3	0.92 ± 0.03	100	0	
B	4	0.108 ± 0.007	0	100	520 and 465 nm LED
	5	0.74 ± 0.04	100	100	
	6	0.71 ± 0.06	100	0	
C	7	0.100 ± 0.007	0	100	520 and 465 nm LED
	8	0.72 ± 0.01	100	100	
	9	0.73 ± 0.04	100		
D	10	0.094 ± 0.005	0	100	465 nm LED
	11	0.43 ± 0.02	100	100	
	12	0.30 ± 0.01	100	0	
E	13	0.09 ± 0.01	0	100	465 nm LED
	14	0.45 ± 0.01	100	100	
	15	0.28 ± 0.01	100	0	
F	16	0.102 ± 0.005	0	100	465 nm LED
	17	0.43 ± 0.01	100	100	
	18	0.28 ± 0.01	100	0	
Q	19	0.051 ± 0.004	0	100	Solar Light Simulator
	20	0.96 ± 0.02	100*	100*	
	21	0.91 ± 0.03	100*	100*	
	22	0.32 ± 0.02	100	0	
P	44	/	100	0	phasor-FLIM
	45	/	0	200	

46	/	100	200

*See chapter 1.2 spot comparison.

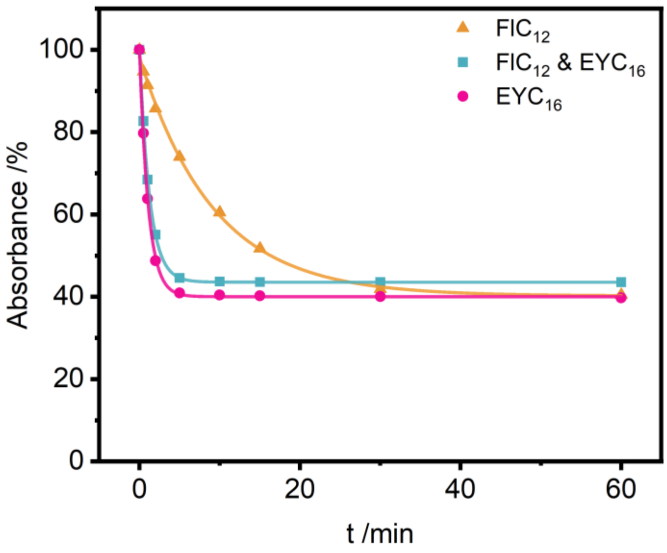

Figure S40: Sample set A; Temporal evolution of the NADH absorbance at 340 nm and mono exponential decay fitting.

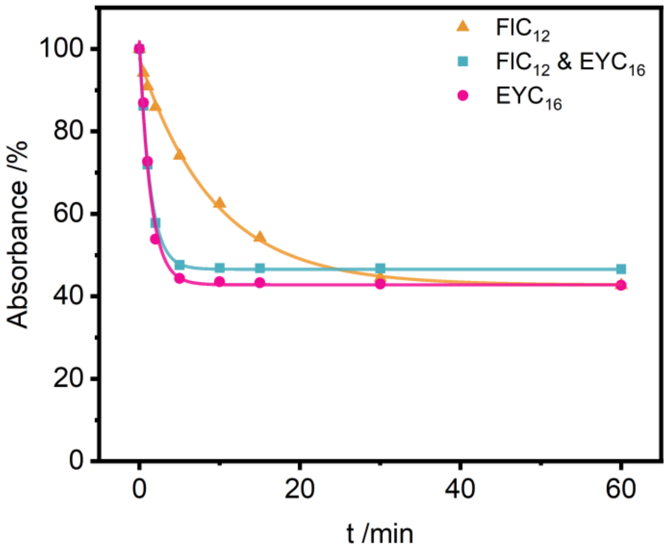

Figure S41: Sample set B; Temporal evolution of the NADH absorbance at 340 nm and mono exponential decay fitting.

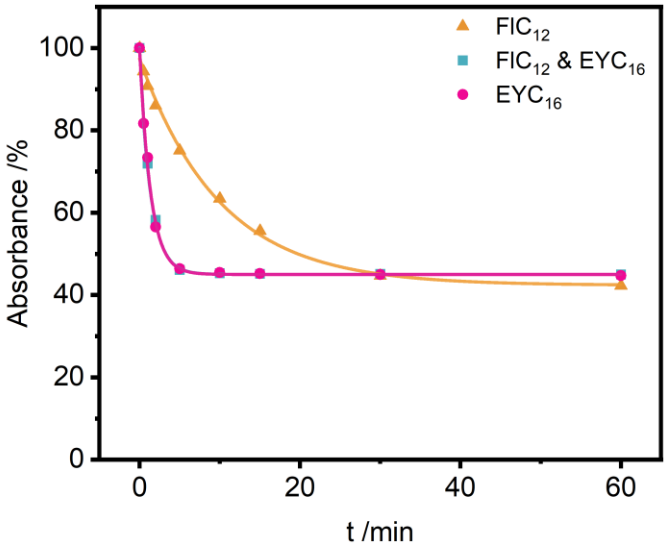

Figure S42: Sample set C; Temporal evolution of the NADH absorbance at 340 nm and mono exponential decay fitting.

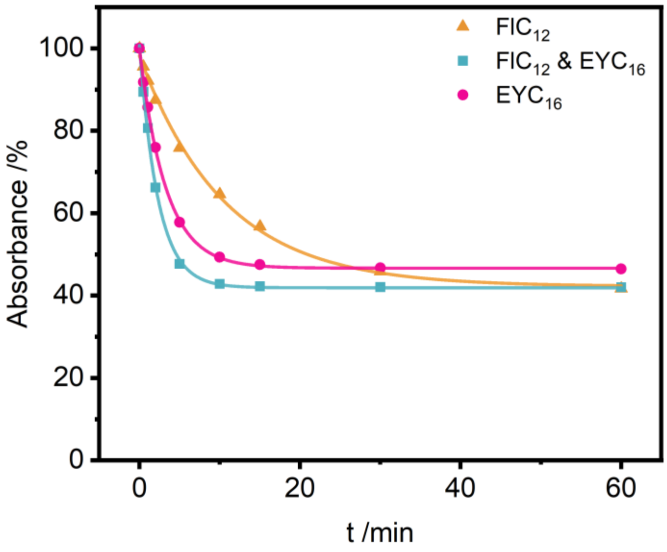

Figure S43: Sample set D; Temporal evolution of the NADH absorbance at 340 nm and mono exponential decay fitting.

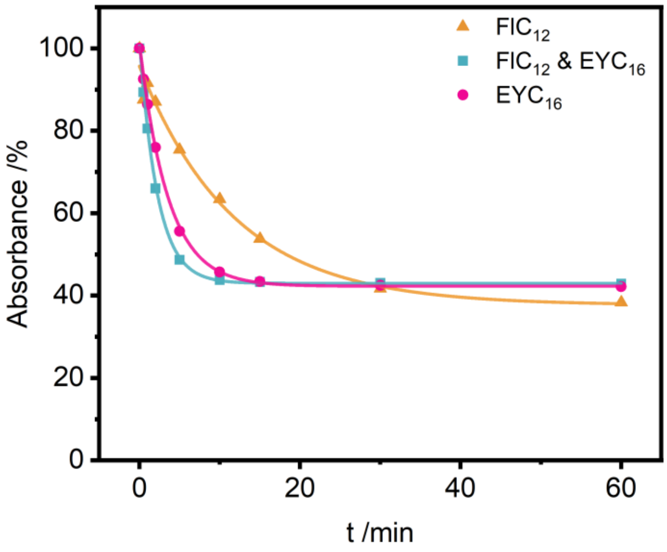

Figure S44: Sample set E; Temporal evolution of the NADH absorbance at 340 nm and mono exponential decay fitting.

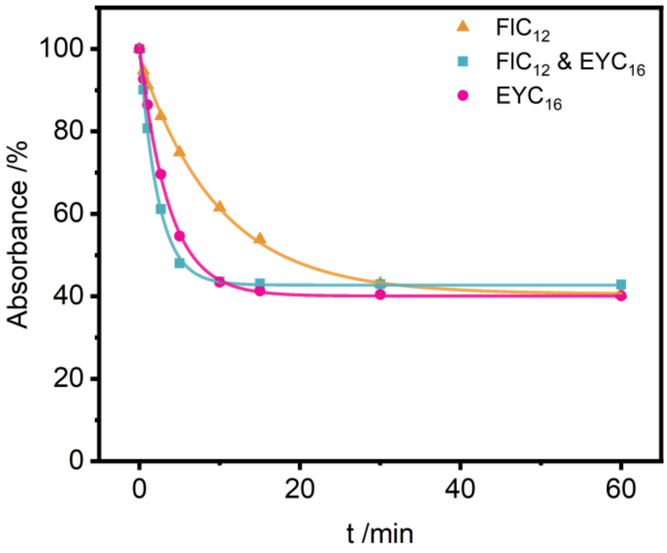

Figure S45: Sample set F; Temporal evolution of the NADH absorbance at 340 nm and mono exponential decay fitting.

15.2 Asymmetric Liposomes

Table S5: Rate constant and experimental data of asymmetric liposomes.

Sample set	Entrance	Fit k /min^{-1}	**X** µL (0.5 mM) EYC_{16}	**Y** µL (0.5 mM) FlC_{12}	Light source
H	23	0.69±0.03	100	0	520 and 465 nm LED
	24	0.76±0.01	100	100	
	25	0.077±0.008	0	100	
I	26	0.76±0.04	100	0	520 and 465 nm LED
	27	0.60±0.02	100	100	
	28	0.049±0.002	0	100	
J	29	0.68±0.02	100	0	520 and 465 nm LED
	30	0.87±0.05	100	100	
	31	0.045±0.003	0	100	
K	32	0.70±0.02	100	0	520 and 465 nm LED
	33	0.90±0.04	100	100	
	34	0.041±0.003	0	100	
L	35	1.7±0.1	100		Solar light Simulator
	36	2.4±0.2	100	100	
	37	0.52 ± 0.05	0	100	
M	38	1.7±0.1*	100		Solar light Simulator
	39	2.31±0.08*	100	100	
	40	0.139±0.009	0	100	
N	41	1.82±0.09	100		Solar light Simulator
	42	2.4±0.2	100	100	
	43	0.17±0.01	0	100	

*Emission data of NADH at 450 nm

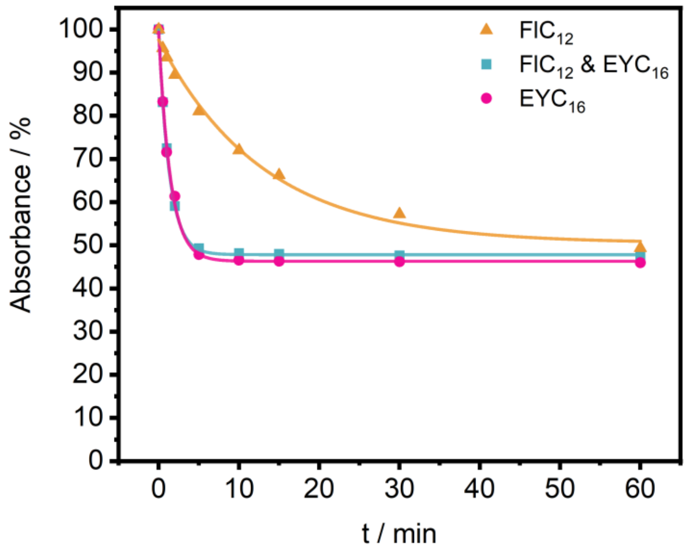

Figure S46: Temporal evolution of the NADH absorbance at 340 nm and mono exponential decay fitting, sample set H.

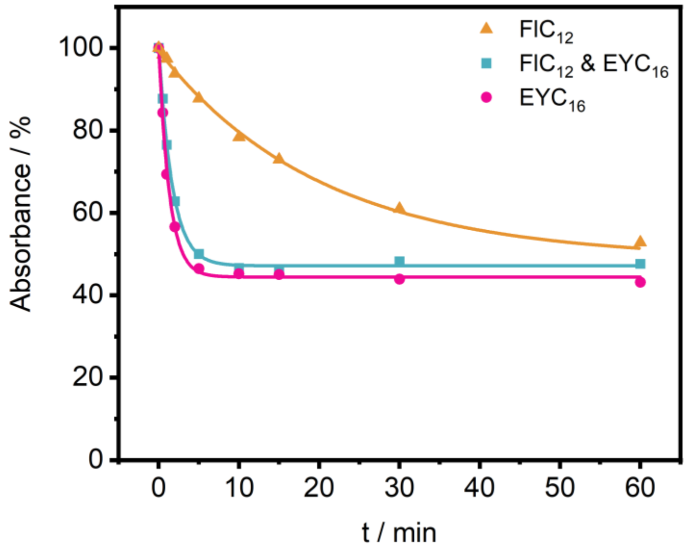

Figure S47: Temporal evolution of the NADH absorbance at 340 nm and mono exponential decay fitting, sample set I.

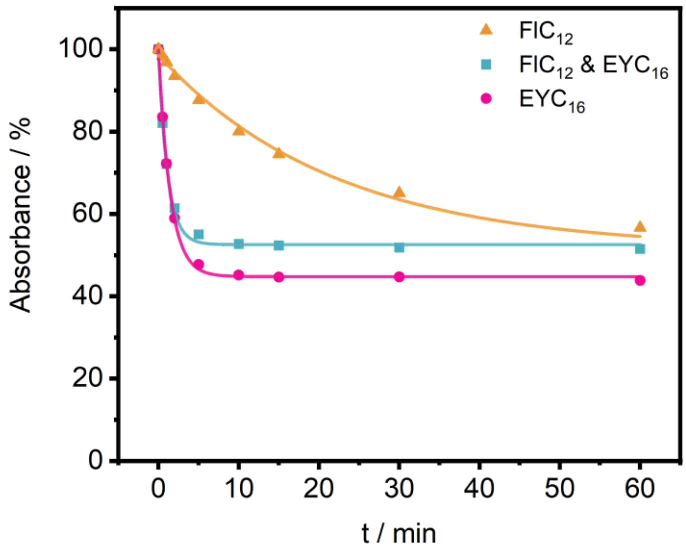

Figure S48: Temporal evolution of the NADH absorbance at 340 nm and mono exponential decay fitting, sample set J.

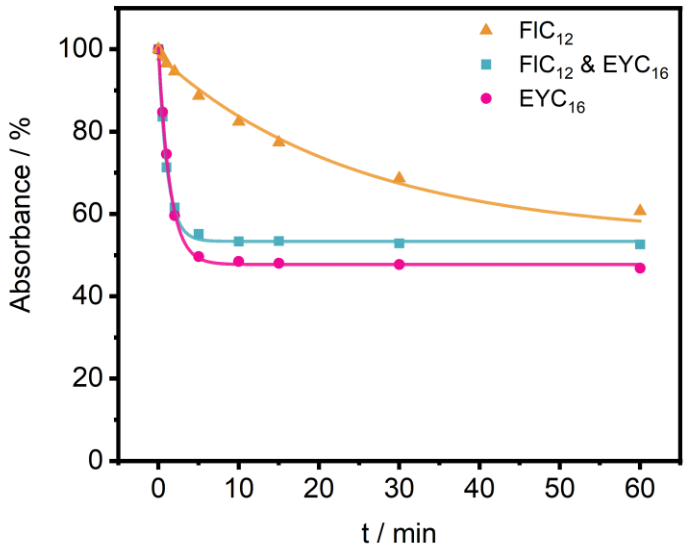

Figure S49: Temporal evolution of the NADH absorbance at 340 nm and mono exponential decay fitting, sample set K.

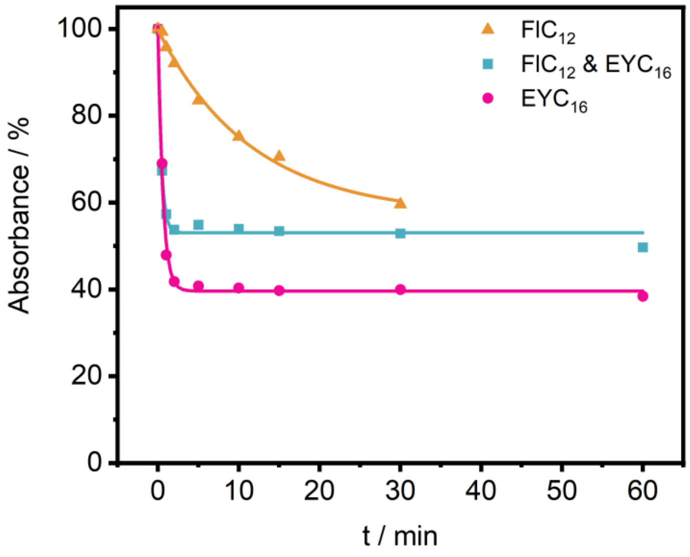

Figure S50: Temporal evolution of the NADH absorbance at 340 nm and mono exponential decay fitting, sample set L.

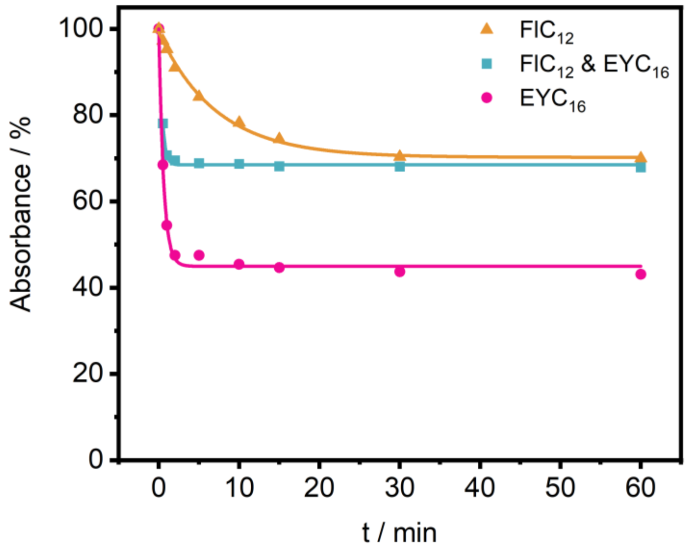

Figure S 51: Temporal evolution of the NADH absorbance at 340 nm of FlC$_{12}$ and temporal evolution of the NADH emission intensity at 450 nm and mono exponential decay fitting, sample set M.

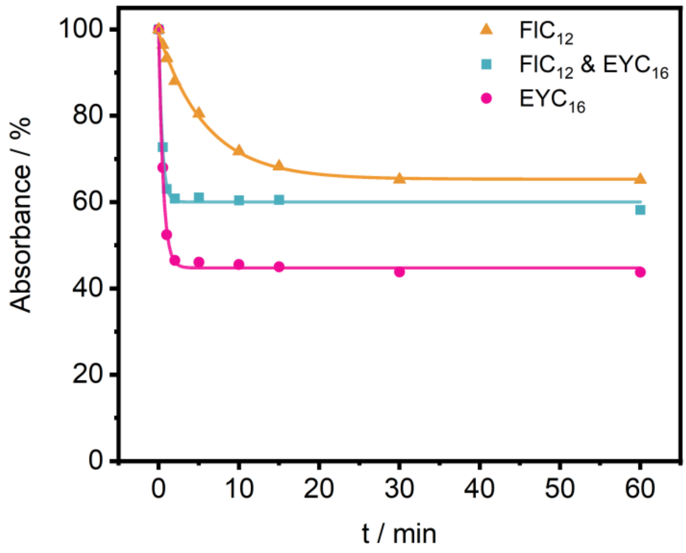

Figure S 52: Temporal evolution of the NADH absorbance at 340 nm and mono exponential decay fitting, entrance N.

S16. Measurements without fluorophores (autooxidation).

For this experiment liposome were prepared as described in 5.2 without any Fluorophore.

The autooxidation of NADH in oxygen atmosphere with irradiation is negligible.

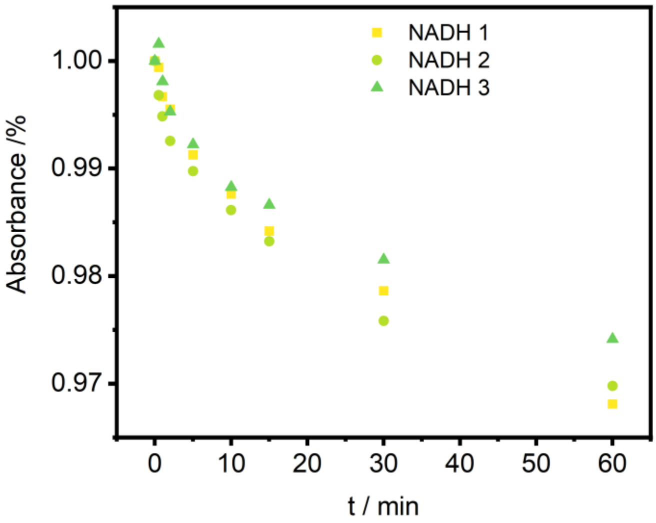

Figure S 53. Temporal change in the UV-vis absorption spectra of 56 mM NADH inside the Liposome (Three samples) without baseline correction.

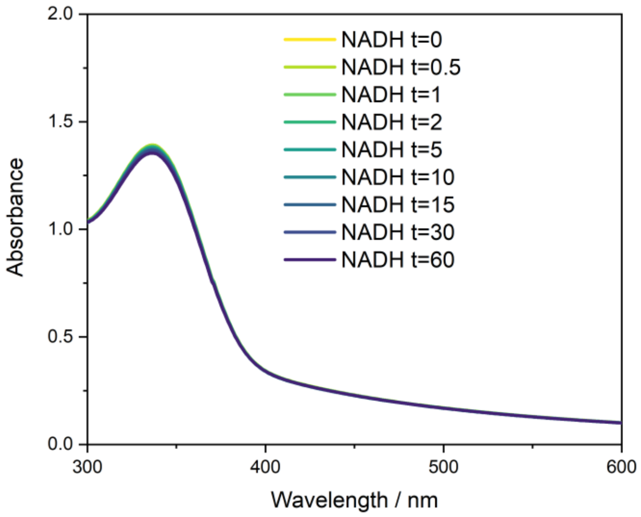

Figure S 54: Example absorbance spectra of liposomes with 56 mM NADH inside without fluorophores.

S17. Photon competition

Using the measured power from the LED and the emission spectrum, we calculated the number of photons at the emission maxima and recalculated the number of photons per wavelength. This process was performed for both LED sources for samples A-C and K-N separately.

Multiplying the transmission with the number of photons at each wavelength, followed by integration, and provided the qualitative number of photons absorbed by each dye. To construct a hypothetical non-interacting liposome, the qualitative number of photons from the overlap integral of both dyes was divided by two and subtracted from the number of photons absorbed by each of the dyes. This value was then divided by the number of photons in an exclusively one dye containing liposome, yielding a factor representing the number of photons available in a mixed system. In samples A-C FlC_{12} has access to 64 % of Photons while EYC_{16} has access to 83 % of the incoming photons compared to one dye only experiments. In samples K-N these factors are 55 % for FlC_{12} and 91 % for EYC_{16}. Those factors were applied to the k to calculate the final hypothetical liposomes.

Using equation Eq. S 14 with h = Planck constant in Js, c = speed of light in ms-1 and λ = wavelength in m we determined the energy of photons at 465.5 nm and 527.5 nm (E_{Photon}) which are the maximum wavelength of our LEDs. As the measurement error of our power meter is ± 2 nm, we need to divide by 5 nm to get to the energy at one specific wavelength. With the incoming Energy per second (POWLED in W) in hand we can now calculate how much photons per second (N_{Photon}) reach our sample at either 465.5 nm or 527.5 nm. (Eq. S 15). Now we can calculate the incoming photons for each wavelength $N_{Photon}(\lambda)$ when we divide the normalized emission of our LED by dividing through N_{Photon} (Eq. S 16). If we now add $N_{Photon}(\lambda)$ at each wavelength from both our LEDs we end up with N_{Photon} total(λ) for each wavelength (Eq. S 17).

$$E_{Photon} = \frac{h \cdot c}{\lambda} \qquad \textbf{Eq. S 14}$$

$$N_{Photon} = \frac{\frac{POW_{LED}}{E_{Photon}}}{5} \qquad \textbf{Eq. S 15}$$

$$N_{Photon}(\lambda) = \frac{Emission_{LED,normalized}(\lambda)}{N_{Photon}} \qquad \textbf{Eq. S 16}$$

$$N_{Photon,total}(\lambda) = N_{Photon,465.5nm}(\lambda) + N_{Photon,527.5nm}(\lambda)$$ **Eq. S 17**

Now we need to have the absorption of our dyes in the membrane. For that we start with the measurement at t=60 min. We then use a mono-exponential fit (Equation 3) from using the data from 800 to 601 nm and 399 to 351 nm for EYC_{16} and 800 to 600 nm and 399 to 326 nm for FlC_{12} construct a baseline which should resemble the Tyndall scattering of the liposomes. We then take the just constructed baseline from the t=60 min measurement and subtract it from the t=0 min measurement. We end up with a Tyndall scattering free absorption of our dyes. As the NADH absorption gives us a good indicator how much liposomes we have in our sample we normalize the absorption spectra to our NADH absorption. As the used concentration are not in the region of linear optics, we calculated the transmission in % ($T(\lambda)_{sample}$) for each wavelength and each dye with Eq. S 18, where Abs = the Tyndall scattering, and liposome amount corrected absorption of our dyes. When we then multiply $N_{Photon,total}(\lambda)$ with our ($T(\lambda)_{sample}$) we end up with the amount of photons the dyes $N_{Photon,absorbed}(\lambda)$ can absorb at each wavelength (Eq. S 19).

$$T(\lambda)_{dye} = 100 - 10^{-(|-2|)}$$ **Eq. S 18**

$$N_{Photon,absorbed}(\lambda) = N_{Photon,total} \cdot T(\lambda)_{dye}$$ **Eq. S 19**

Now we can calculate how much of the total photons each dye in mixture gets: If we form the integral of the $N_{Photon,absorbed}(\lambda)$ for each dye (I_{FlC12} and IEYC16), we have the total amount of Photons each dye absorbs without the other dye present. If we now form a new function which consists of the absorption of FlC_{12} from 800 to 494 nm and the absorption of EYC_{16} from 493 to 250 nm, where 493 nm represents the turning point where the EYC_{16} absorption is greater than the FlC_{12} absorption, and integrate this function, we get the number of photons both dyes could absorb (I_{Mix}). If we assume now that each dye has the same probability to absorb those photons, we can calculate how much photons each dye gets in the mixture (Figure S55, Figure S 56). For that we subtract half of I_{Mix} from the integral of I_{FlC12} or I_{EYC16} and divide by I_{FlC12} or I_{EYC16} to get P_{uFlC12} = 0.646 and P_{uEYC16} = 0.826 (Eq. S 20 and Eq. S 21) for symmetric liposomes. For asymmetric liposomes these values will be P_{uFlC12} = 0.549 P_{uEYC16} = 0.910.

$$P_{uEYC16} = \frac{(I_{EYC16}) - \left(\frac{I_{Mix}}{2}\right)}{(I_{EYC16})}$$ **Eq. S 20**

$$P_{uFlC12} = \frac{(I_{FlC12}) - \left(\frac{I_{Mix}}{2}\right)}{(I_{FlC12})}$$ **Eq. S 21**

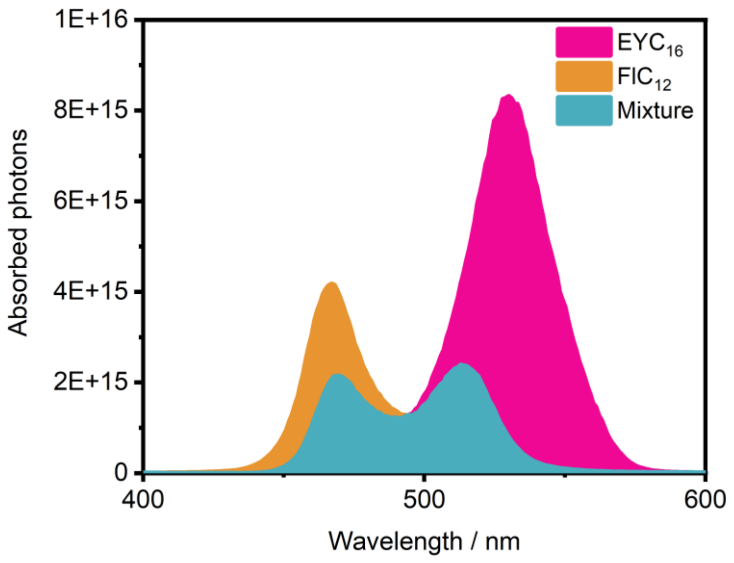

Figure S55: Absorbed photons ($N_{Photon,absorbed}$(λ)) of EYC_{16}, FlC_{12} and the mixture symmetric liposomes.

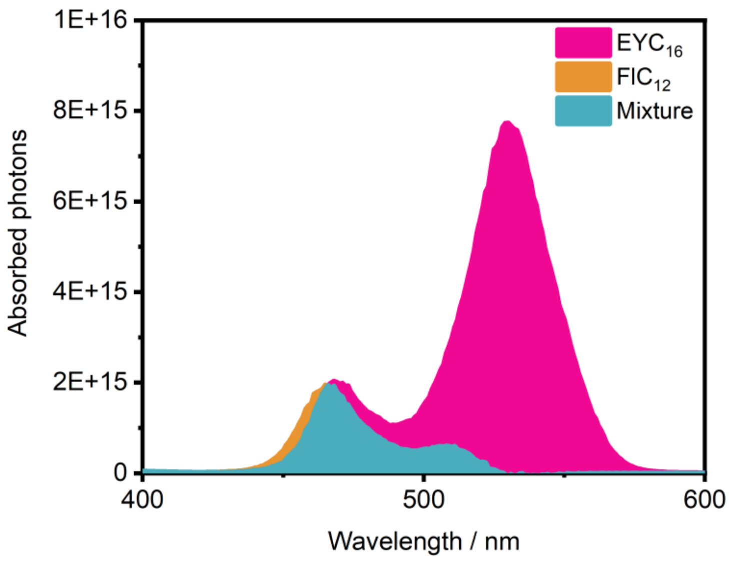

Figure S 56: Absorbed photons ($N_{Photon,absorbed}$(λ)) of EYC_{16}, FlC_{12} and the mixture asymmetric liposomes.

With Equations Eq. S 22, Eq. S 23 and Eq. S 24 we can now calculate the impact on k for hypothetical reference when two dyes with similar absorption coexist in the same sample. When photon competition is not considered P is 1.

$$f(t) = 100 \cdot e^{-(P_{uFlC12}k_{Fl}+P_{uEYC16}k_{EY})t} + 0 \quad \textbf{Eq. S 22}$$

$$f(t) = 100 \cdot e^{-(P_{uFlC12}k_{Fl}-P_{uFlC12}k_{\Delta Fl}+P_{uEYC16}k_{EYC16}-P_{uEYC16}k_{\Delta EYC16})t} + 0 \quad \textbf{Eq. S 23}$$

$$f(t) = 100 \cdot e^{-(P_{uFlC12}k_{FlC12}+P_{uFlC12}k_{\Delta FlC12}+P_{uEYC16}k_{EYC16}+P_{uEYC16}k_{\Delta EYC16})t} + 0 \quad \textbf{Eq. S 24}$$

For evaluation of the effective usage of the light sources at the cuvette position, emitted power and the emission spectra was used to calculate the number of emitted photons per wavelength. Those values were than multiplied with the percentage of absorbance at the respective wavelength of the dye. The resulting used photon per wavelength data were summed up to the final usable power.

The power of the LEDs was measured at the emitted maximum wavelength as descripted in chapter 1.1, with a variance of ±2 nm range. The power measured therefore represents the average power of a 5 nm window, centred around 465 nm or 527 nm. This was used to calculate the number of photons of this area.

$$n_{Photonesat465nm} = \frac{P_{465} * \nu_{465}}{hc} * 0.1 * 0.2 = 1.83^{16}s^{-1} \quad \textbf{Eq. S 25}$$

P as the measured power of the respective LED in Watt. *h* as the Planck constant in J/s. *c* as the speed of light in m/s and *v* as the wavelength in m. Factor 0.1 represents the absorbance of the OD1 filter and factor 0.2 respects the fact that the power was measured over a range of 5 nm.

Now every measured intensity, Emi_{nm}, of the LED emission spectra was set in relation to the intensity at 465 nm, Emi_{465}, and the calculated photon count.

$$n_{Photonesánm} = \frac{Emi_{nm}}{Emi_{465}} * n_{Photonesat465nm}$$ **Eq. S 26**

The emission spectra were normalized to 1 at its highest signal, *Emi*$_{normalized}$. The emission at each wavelength was then multiplied with the energy of one photon at the respective wavelength. The sum of this power was taken in ratio to the power of the beam at the cuvette, which was set to 100 mW as descripted in chapter 1.1 .That leads to the photon count per wavelength.

$$P_{Photonatnm} = \frac{hc}{\nu}$$ **Eq. S 27**

$$P_{spectrum} = \sum P_{Photonatnm} * Emi_{normalized}$$ **Eq. S 28**

The absorbance, at the respective wavelength *Abs*$_{at\ nm}$ *in %,* of the respective dye/dyes, was multiplied with the photon count at this wavelength, $n_{Photones\ at\ nm}$.

$$n_{Photoneseffectivánm} = |Abs_{atnm}| * n_{Photonesatnm}$$ **Eq. S 29**

As a last step, the effective photon count was multiplied with their respective energy and summed up to the final power usable for each respective dye and light source.

$$P_{spectrum} = \sum \frac{hc}{\nu_{atnm}} * n_{Photoneseffectivatnm}$$ **Eq. S 30**

Table S 6: Listed are the effective usable power in dependency of the light source and dye in mW.

[mW]	$P_{spectrum}$ FlC$_{12}$	$P_{spectrum}$ EYC$_{16}$	$P_{spectrum}$ FlC$_{12}$+ EYC$_{16}$
465 nm LED	0.023	0.021	0.023
527 nm LED	0.005	0.014	0.014
465+527 nm LED	0.028	0.035	0.037
Solar Light Simulator	0.075	0.085	0.086

S18. Protection of eosin Y from NADH due to membrane

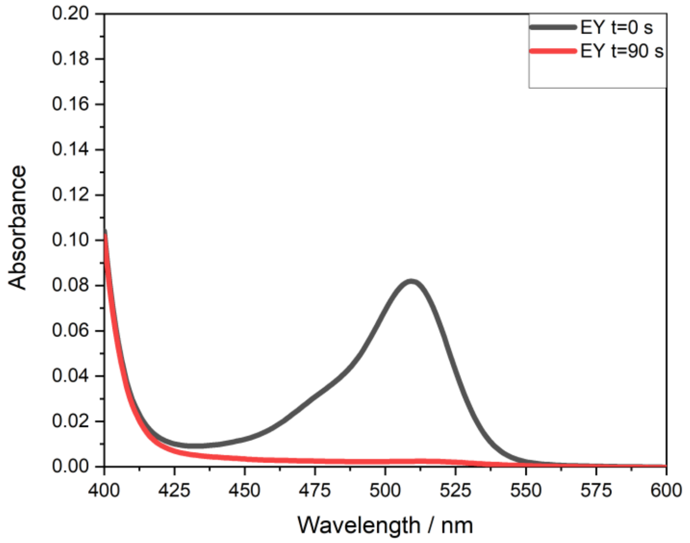

Figure S57: EosinY (without C_{16}) in presence of 1mM NADH and oxygen in a 100 mM pH 8.99 phosphate buffer and illumination with both LEDs as described above.

S19. Hydride transfer from NADH to eosin Y - NMR experiments.

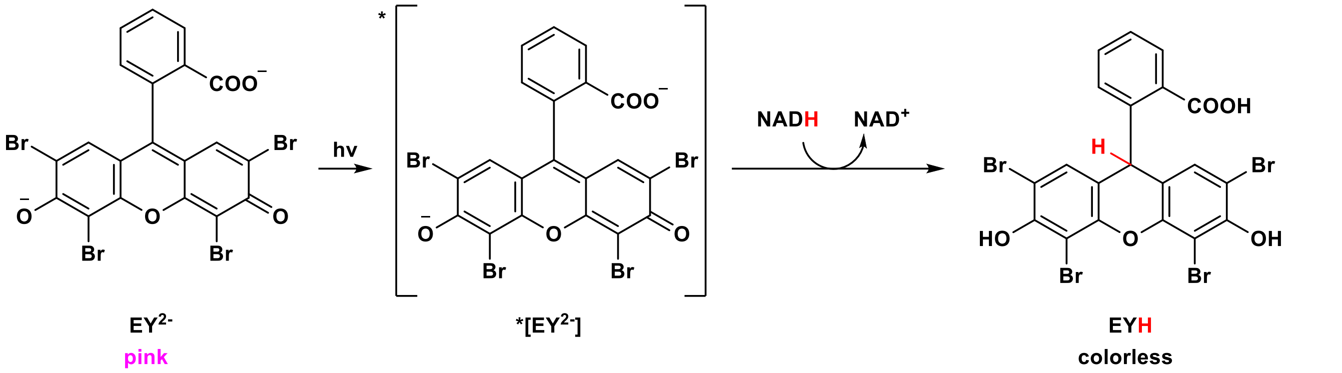

Figure S 58: Proposed reaction for a hydride transfer from NADH to EYC_{16}.

For this experiment the procedure of of Kim et al.[44] was adapted, a 1mM EosinY solution was prepared in D_2O for one NMR tube and 1 mM of EosinY and 56 mM of NADH for the other NMR Tube. After 2 h both NMR tubes were irradiated with the usual setup (465 nm + 527 nm with OD Filter of 1) for 90 s. Then, one drop of 35% HCl in H_2O was added and centrifuged in a brown Eppendorf vial at 17000 g for 3 min. The residue was washed three times with 4°C 1 M HCl and then dried at the rotavap for 30 min. The residue was taken up in DMSO-d_6 and a ^{1}H-NMR was taken. The NMR shows the peaks of the reduced Eosin Y (EYH) as well as protonated but not reduced EosinY.

^{1}H NMR (400 MHz, DMSO-d_6, TMS) δ [ppm] = 10.18 (s, 2H), 7.84 (d, J = 1.5 Hz, 1H), 7.46 (d, J = 1.5 Hz, 1H), 7.34 (d, J = 1.3 Hz, 1H), 7.19 (s, 2H), 7.01 (d, J = 7.7 Hz, 1H), 6.28 (s, 1H).

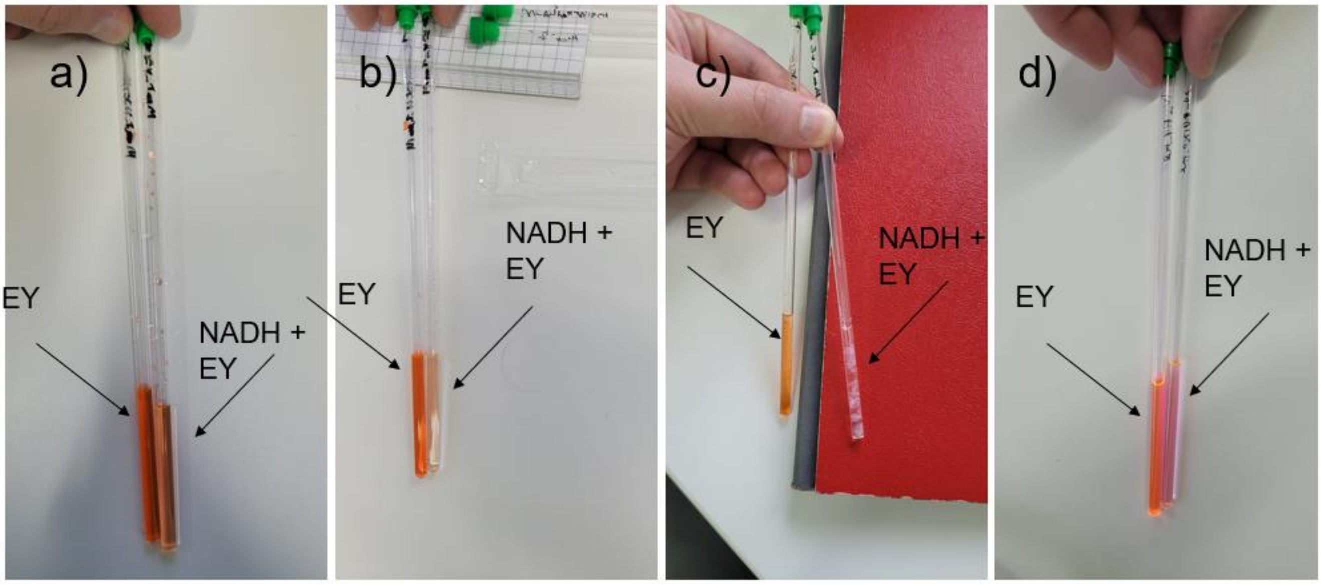

Figure S 59: NMR tubes filled with 1mM EosinY and 1mM EosinY and 56 mM NADH in D_2O(a)-c)) and DMSO-d_6 (d)). a) Shortly after preparing the solution. b) After waiting for 2 h and irradiate for 90 s with 465 nm and 527 nm. C) After adding one drop of 35 % HCl in H_2O. d) After washing three times with cold 1 M HCl and redissolving in DMSO-d_6.

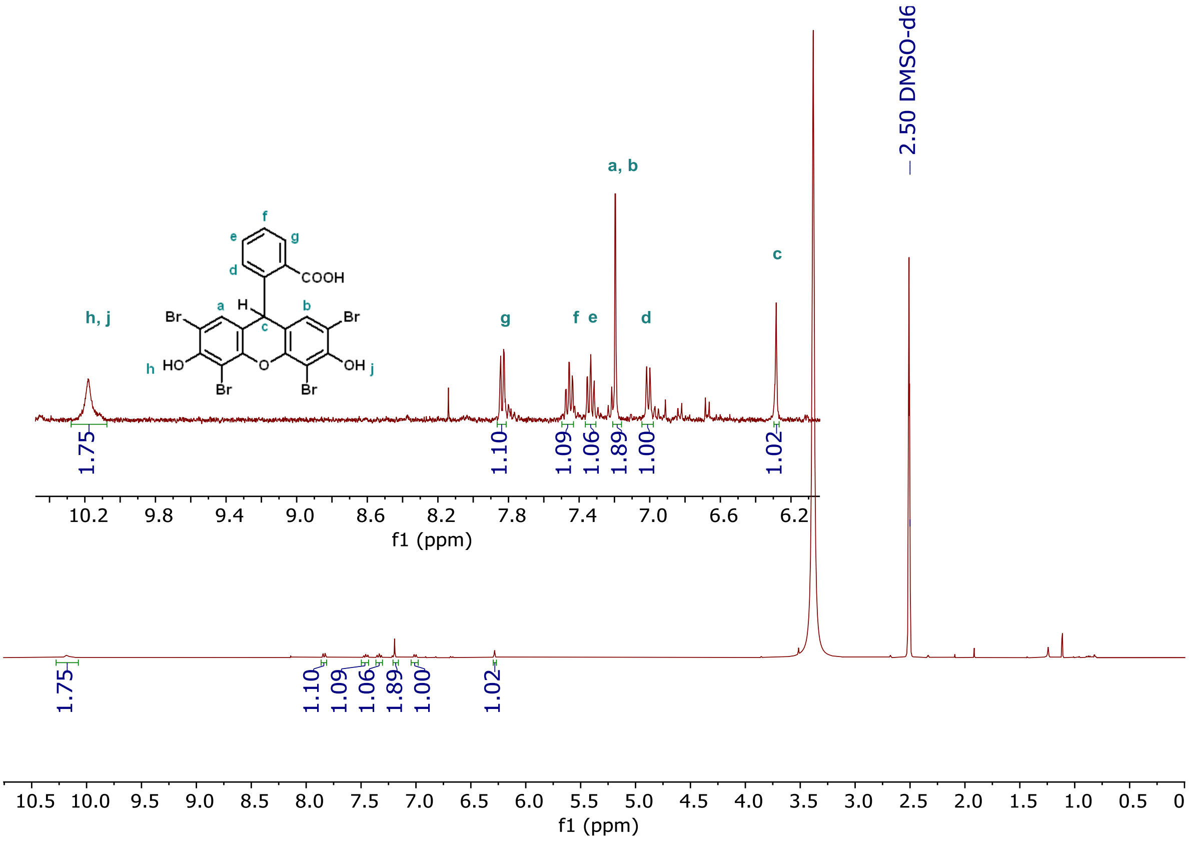

Figure S 60: ^{1}H-NMR of d) in DMSO-d_6. There is also not reduced, but protonated EosinY present, which is in agreement with Kerrebroeck et al..[45] The Signals between 6.6 and 7.0 ppm are also present in the NMR of Kim et al..[44]

S20. EYC_{16} lifetimes in liposomes

Liposomes are prepared by following:

A mixture of 1 ml of a 5 mM DPPC stock solution in chloroform, 1 ml of a 0.05 mM 14:0 PEG-2000 PE stock solution in chloroform and 100 µl of a 0.5 mM EYC_{16} in methanol were combined in a round bottle flask and dried first by using the rotary evaporator followed by 0.05 mbar oil pump vacuum for at least eight hours. To the residue either 0.5 ml of 56 mM NADH in phosphate buffer or 0.5 ml phosphate buffer was added. Three freeze-thaw cycles were performed with the mixture by using liquid nitrogen and a 55°C water bath. Afterwards, the liposomes were extruded 11 times with a membrane filter with 0.2 µm pore size. One sample with and one sample without NADH were degassed and then put overnight in a dark glovebox filled with argon to ensure all oxygen is removed. The other two samples were stored dark overnight in air atmosphere.

Table S 7 Obtained Data from fitting of the kinetic traces of 1% EYC_{16} in DPPC Liposomes in air or argon and with or without NADH. The lifetimes τ_1/τ_2 obtained from the biexponential Fit were calculated with this formula - $\boldsymbol{\tau = \tau_1 a_1 + \tau_2 a_2}$ - for the average weighted lifetime where a_1 and a_2 are the relative amplitudes.

**Sample**	**τ_1 (a_1)/ ns**	**τ_2 (a_2) /ns**	**τ /ns**
EYC_{16} Air	0.40 (36.9)	1.75 (63.2)	1.25
EYC_{16} Argon	0.41 (40.3)	1.68 (59.7)	1.17
EYC_{16} Air NADH	0.42 (30.0)	1.98 (70.0)	1.51
EYC_{16} Argon NADH	0.42 (29.5)	1.92 (70.5)	1.48

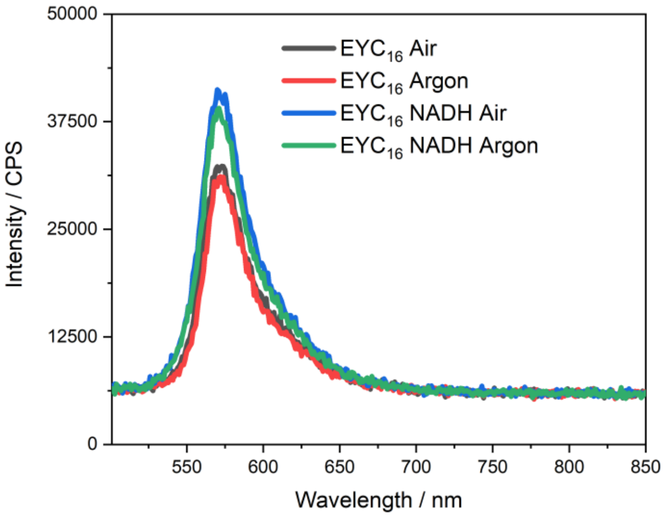

Figure S 61: Emission of 1% EYC_{16} DPPC Liposome samples in air or argon and with or without 56 mM NADH.

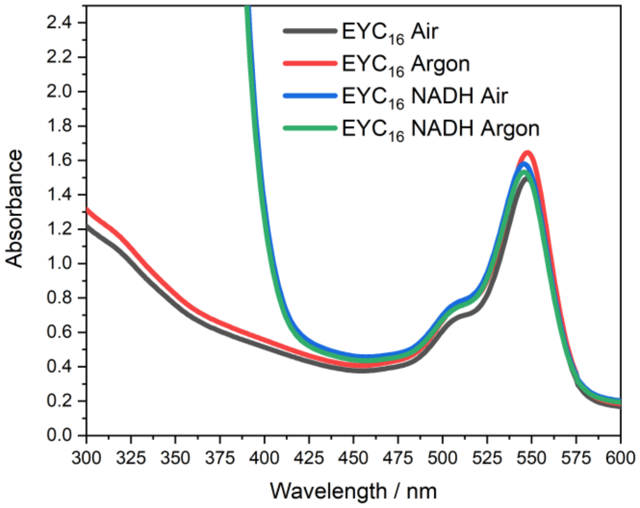

Figure S 62: Absorption of 1% EYC_{16} DPPC Liposome samples in air or argon and with or without 56 mM NADH.

S21. Self Quenching of EosinY

Liposomes with 1 % FlC_{12} in a 10 mM phosphate buffer at pH 7.7

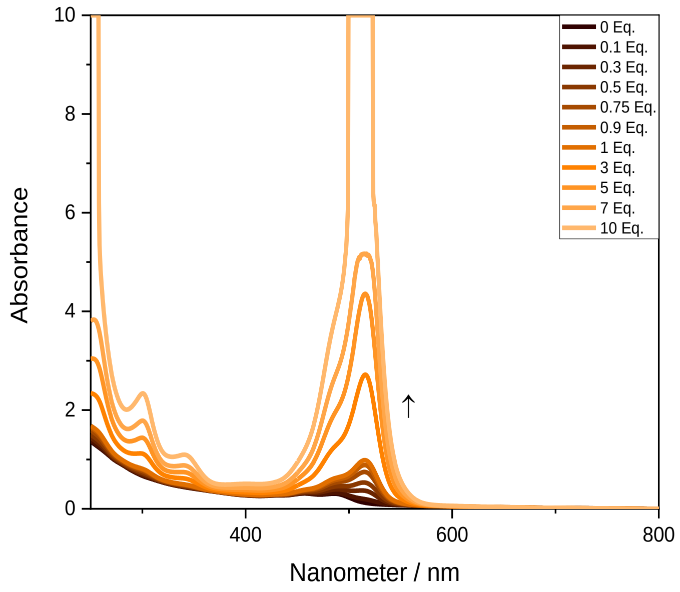

Figure S63: Absorbance of adding of EosinY equivalents to a 1 % containing FlC_{12} liposome.

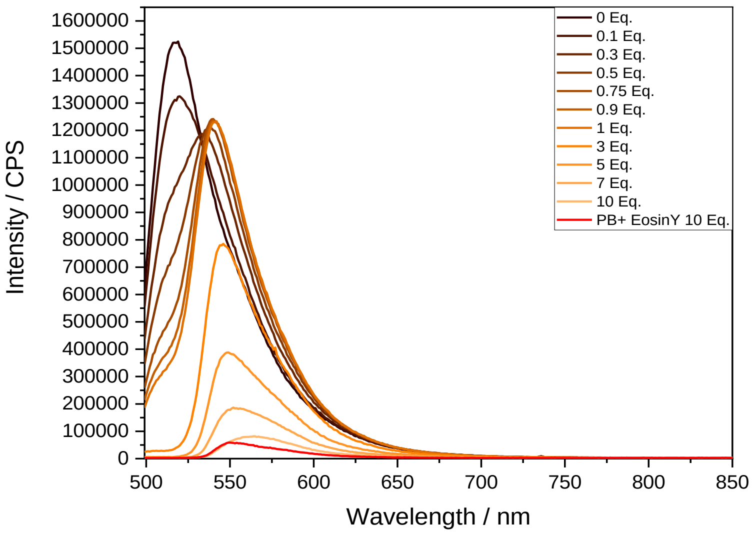

Figure S64: Emission of adding of EosinY equivalents to a 1 % containing FlC_{12} liposome. λ_{exc} = 497 nm

S22. 1% eosin Y in the bulk 56 mM NADH inside

Liposomes are prepared by following:

A mixture of 1 ml of a 5 mM DPPC stock solution in chloroform, 1 ml of a 0.05 mM 14:0 PEG-2000 PE stock solution in chloroform were combined in a round bottom flask and dried first by using the rotary evaporator followed by 0.05 mbar oil pump vacuum for at least eight hours. To the residue 0.5 ml of 56 mM NADH in phosphate buffer was added. Three freeze-thaw cycles were performed with the mixture by using liquid nitrogen and a 55°C water bath. Afterwards, the liposomes were extruded 11 times with a membrane filter with 0.2 µm pore size. A G25 Sephadex column (6 cm length, 2 cm diameter) with phosphate buffer as mobile phase was used to exchange the surrounding solvent of the liposomes. Then 100 µl of a 0.5 mM EosinY in phosphate buffer was added and diluted to 3 ml.

Sample	Fit k /min^{-1}
1/2	0.054 ± 0.002
2/2	0.050 ± 0.002

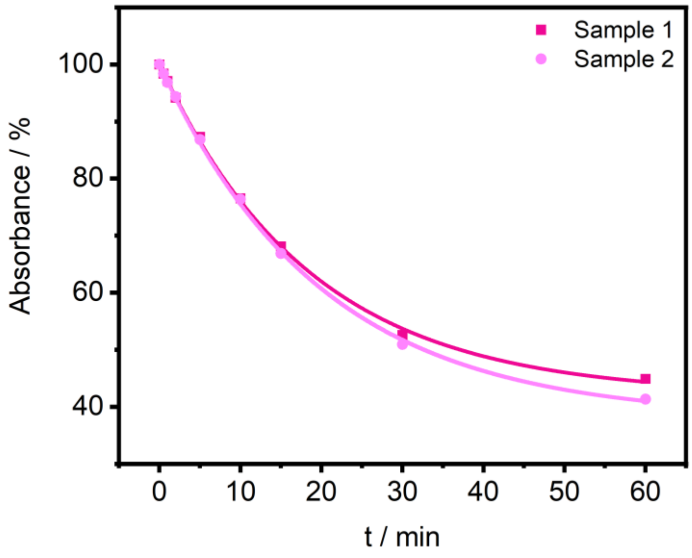

Figure S 65: Sample 1 and 2; Temporal evolution of the NADH absorbance at 340 nm and mono exponential decay fitting

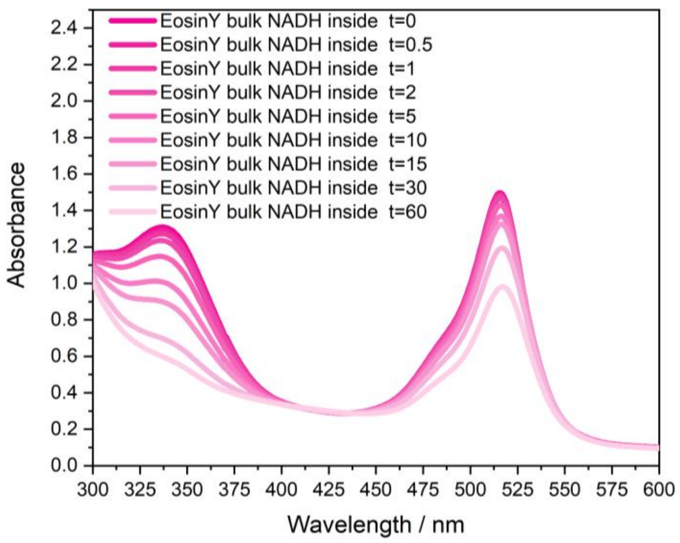

Figure S 66: Temporal change in the UV-vis absorption spectra of EosinY in the bulk and NADH inside the Liposome (sample 1 of 2) without baseline correction.

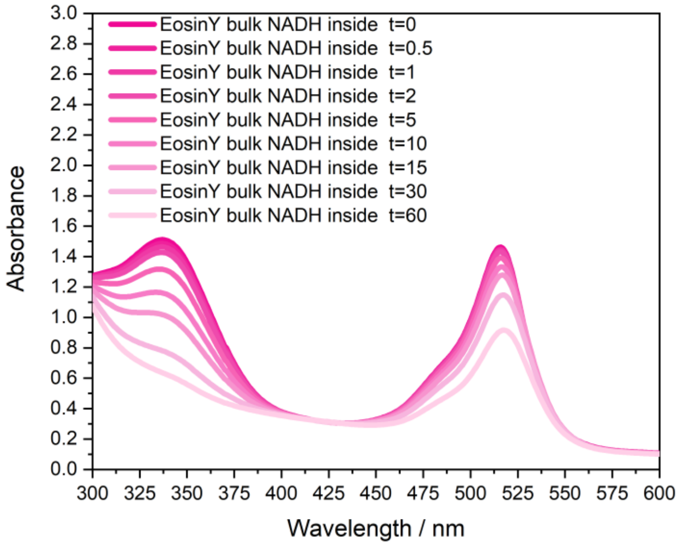

Figure S 67: Temporal change in the UV-vis absorption spectra of EosinY in the bulk and NADH inside the Liposome (sample 2 of 2) without baseline correction.

S23. 0.1 mM NADH in bulk, 1% EYC_{16} in membrane

For this experiment liposome were prepared as described in 5.2 without any NADH and 1 % EYC_{16}. After SEC the solution was diluted to 2.7 ml and 300 µl of a 1mM NADH solution (in the used buffer) was added for the final concentration of 0.1 mM of NADH in the bulk.

Table S 8: K values of three samples of DPPC liposomes with 1% EYC_{16} and 0.1 mM NADH in bulk.

Sample	Fit k /min^{-1}
Sample 1	0.0196 ± 0.0003
Sample 2	0.0194 ± 0.0007
Sample 3	0.0221 ± 0.0009

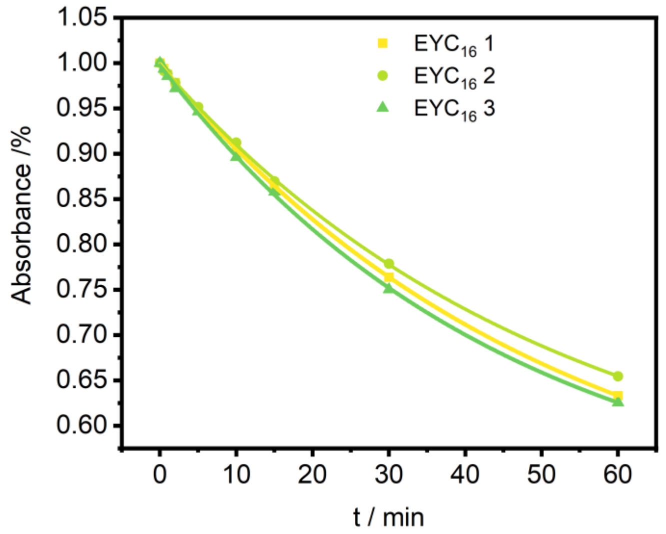

Figure S 68: Temporal change in the UV-vis absorption spectra of 0.1mM NADH in the bulk and 1% EYC_{16} in the membrane (Three samples) without baseline correction

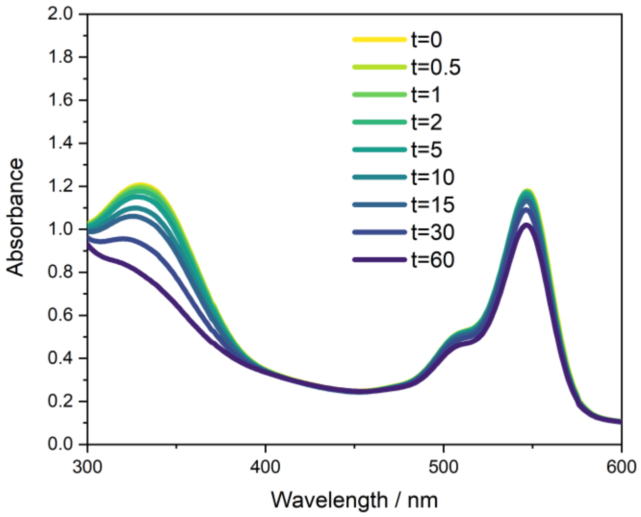

Figure S 69: example absorbance spectra of liposomes with 0.1 mM NADH in the bulk and 1%EYC$_{16}$ in the membrane.

S24. Sodium azide tests

A liposome was prepared as descripted in Chapter 5.2 (symmetric liposome) with **X**;**Y** chosen as 0.25 µl. In Addition to the NADH solved phosphorus buffer sodium azide (56mM) was added.

Position	t /min^{-1}
FlC_{12} EYC_{16}	1.36 ± 0.04
FlC_{12} EYC_{16} Azide	0.103 ± 0.009

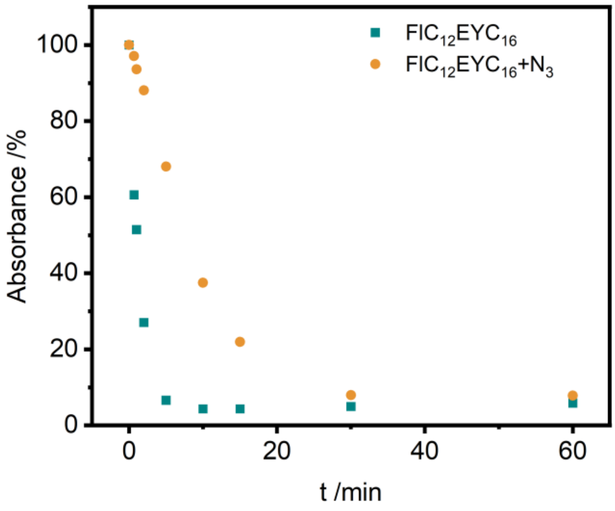

Figure S70: Time trace of NADH Absorbance with or without sodium azide added.

S25. References

[1] R. E. P. Nau **2024**, DOI https://github.com/Pannwitz-group/Simic-box-2023.

[2] S. S. S. Tan, P. C. Hauser, K. Wang, K. Fluri, K. Seiler, B. Rusterholz, G. Suter, M. Krüttli, U. E. Spichiger, W. Simon, "Reversible optical sensing membrane for the determination of chloride in serum" *Analytica Chimica Acta* **1991**, *255*, 35–44.

[3] D. Garcìa-Fresnadillo, Y. Georgiadou, G. Orellana, A. M. Braun, E. Oliveros, "Singlet-Oxygen (1 Δ g) Production by Ruthenium(II) complexes containing polyazaheterocyclic ligands in methanol and in water" *Helvetica Chimica Acta* **1996**, *79*, 1222–1238.

[4] R. E. P. Nau, J. Bösking, A. Pannwitz, "Compartmentalization Accelerates Photosensitized NADH to NAD+ Conversion" *ChemPhotoChem* **2022**, *6*, e202200158.

[5] G. A. Anido, S. B. Rosalki, E. J. V. Kampen, M. Rubin, Eds. in *Quality Control in Clinical Chemistry*, De Gruyter, **1976**, pp. 235–240.

[6] D. Marsh, *Handbook of Lipid Bilayers*, CRC Press, **2013**.
[7] S. K. Filippov, R. Khusnutdinov, A. Murmiliuk, W. Inam, L. Ya. Zakharova, H. Zhang, V. V. Khutoryanskiy, "Dynamic light scattering and transmission electron microscopy in drug delivery: a roadmap for correct characterization of nanoparticles and interpretation of results" *Mater. Horiz.* **2023**, *10*, 5354–5370.
[8] S. Peretz Damari, D. Shamrakov, M. Varenik, E. Koren, E. Nativ-Roth, Y. Barenholz, O. Regev, "Practical aspects in size and morphology characterization of drug-loaded nano-liposomes" *International Journal of Pharmaceutics* **2018**, *547*, 648–655.
[9] E. Kim, O. Graceffa, R. Broweleit, A. Ladha, A. Boies, S. P. Mudakannavar, R. J. Rawle, "Lipid loss and compositional change during preparation of simple two-component liposomes" *Biophysical Reports* **2024**, *4*, 100174.
[10] E. Krieger, J. E. Nielsen, C. A. E. M. Spronk, G. Vriend, "Fast empirical pKa prediction by Ewald summation" *Journal of Molecular Graphics and Modelling* **2006**, *25*, 481–486.
[11] S. Dutta, B. Watson, S. Mattoo, J.-C. Rochet, "Calcein Release Assay to Measure Membrane Permeabilization by Recombinant Alpha-Synuclein" *BIO-PROTOCOL* **2020**, *10*, DOI 10.21769/BioProtoc.3690.
[12] N. Sinambela, M. Nau, G. Haug, M. Linseis, P. Koblischek, R. F. Winter, A. Pannwitz, "Light-driven electron transfer in a lipid bilayer with mixed valence molecular wires" *Sustainable Energy & Fuels* **2025**, *9*, 2302–2315.
[13] N. Sinambela, R. Jacobi, D. Sorsche, L. González, A. Pannwitz, "Photoinduced Electron Transfer Across Phospholipid Bilayers in Anaerobic and Aerobic Atmospheres" *Angewandte Chemie International Edition* **2025**, *64*, DOI 10.1002/anie.202423393.
[14] S. Ranjit, L. Malacrida, D. M. Jameson, E. Gratton, "Fit-free analysis of fluorescence lifetime imaging data using the phasor approach" *Nat Protoc* **2018**, *13*, 1979–2004.
[15] R. M. Clegg, "Fluorescence resonance energy transfer" *Current Opinion in Biotechnology* **1995**, *6*, 103–110.
[16] S. Jo, T. Kim, V. G. Iyer, W. Im, "CHARMM-GUI: A web-based graphical user interface for CHARMM" *J Comput Chem* **2008**, *29*, 1859–1865.
[17] E. L. Wu, X. Cheng, S. Jo, H. Rui, K. C. Song, E. M. Dávila-Contreras, Y. Qi, J. Lee, V. Monje-Galvan, R. M. Venable, J. B. Klauda, W. Im, "CHARMM-GUI *Membrane Builder* toward realistic biological membrane simulations" *J. Comput. Chem.* **2014**, *35*, 1997–2004.
[18] D. A. Case, H. M. Aktulga, K. Belfon, et al., *Amber 2023*, University of California, **2023**.
[19] J. Wang, R. M. Wolf, J. W. Caldwell, P. A. Kollman, D. A. Case, "Development and testing of a general amber force field" *Journal of Computational Chemistry* **2004**, *25*, 1157–1174.
[20] J. Wang, W. Wang, P. A. Kollman, D. A. Case, "Automatic atom type and bond type perception in molecular mechanical calculations" *Journal of Molecular Graphics and Modelling* **2006**, *25*, 247–260.
[21] A. D. Becke, "Density-functional thermochemistry. III. The role of exact exchange" *The Journal of Chemical Physics* **1993**, *98*, 5648–5652.
[22] C. Lee, W. Yang, R. G. Parr, "Development of the Colle-Salvetti correlation-energy formula into a functional of the electron density" *Physical Review B* **1988**, *37*, 785–789.
[23] J. P. Perdew, "Density-functional approximation for the correlation energy of the inhomogeneous electron gas" *Physical Review B* **1986**, *33*, 8822–8824.
[24] S. Grimme, S. Ehrlich, L. Goerigk, "Effect of the damping function in dispersion corrected density functional theory" *Journal of Computational Chemistry* **2011**, *32*, 1456–1465.

[25] F. Weigend, R. Ahlrichs, “Balanced basis sets of split valence, triple zeta valence and quadruple zeta valence quality for H to Rn: Design and assessment of accuracy” *Physical Chemistry Chemical Physics* **2005**, *7*, 3297.
[26] F. Weigend, “Accurate Coulomb-fitting basis sets for H to Rn” *Physical Chemistry Chemical Physics* **2006**, *8*, 1057.
[27] M. J. Frisch, G. W. Trucks, H. B. Schlegel, et al., *Gaussian 16, Revision C.01*, Wallingford, **2016**.
[28] C. J. Dickson, R. C. Walker, I. R. Gould, “Lipid21: Complex Lipid Membrane Simulations with AMBER” *Journal of Chemical Theory and Computation* **2022**, *18*, 1726–1736.
[29] S. Izadi, R. Anandakrishnan, A. V. Onufriev, “Building Water Models: A Different Approach” *The Journal of Physical Chemistry Letters* **2014**, *5*, 3863–3871.
[30] J.-P. Ryckaert, G. Ciccotti, H. J. C. Berendsen, “Numerical integration of the cartesian equations of motion of a system with constraints: molecular dynamics of n-alkanes” *Journal of Computational Physics* **1977**, *23*, 327–341.
[31] G. M. Torrie, J. P. Valleau, “Nonphysical sampling distributions in Monte Carlo free-energy estimation: Umbrella sampling” *Journal of Computational Physics* **1977**, *23*, 187–199.
[32] S. Kumar, J. M. Rosenberg, D. Bouzida, R. H. Swendsen, P. A. Kollman, “THE weighted histogram analysis method for free-energy calculations on biomolecules. I. The method” *J Comput Chem* **1992**, *13*, 1011–1021.
[33] A. Grossfield, “WHAM: the weighted histogram analysis method Version 1.1.3” **2025**, 126.
[34] Th. Förster, “Energiewanderung und Fluoreszenz” *Die Naturwissenschaften* **1946**, *33*, 166–175.
[35] Th. Förster, “Zwischenmolekulare Energiewanderung und Fluoreszenz” *Annalen der Physik* **1948**, *437*, 55–75.
[36] T. Förster, “Experimentelle und theoretische Untersuchung des zwischenmolekularen Übergangs von Elektronenanregungsenergie” *Zeitschrift für Naturforschung A* **1949**, *4*, 321–327.
[37] D. F. Kienle, J. V. De Souza, E. B. Watkins, T. L. Kuhl, “Thickness and refractive index of DPPC and DPPE monolayers by multiple-beam interferometry” *Anal Bioanal Chem* **2014**, *406*, 4725–4733.
[38] T. Yanai, D. P. Tew, N. C. Handy, “A new hybrid exchange–correlation functional using the Coulomb-attenuating method (CAM-B3LYP)” *Chemical Physics Letters* **2004**, *393*, 51–57.
[39] T. H. Dunning, “Gaussian basis sets for use in correlated molecular calculations. I. The atoms boron through neon and hydrogen” *The Journal of Chemical Physics* **1989**, *90*, 1007–1023.
[40] R. A. Kendall, T. H. Dunning, R. J. Harrison, “Electron affinities of the first-row atoms revisited. Systematic basis sets and wave functions” *The Journal of Chemical Physics* **1992**, *96*, 6796–6806.
[41] A. Aghigh, S. Bancelin, M. Rivard, M. Pinsard, H. Ibrahim, F. Légaré, “Second harmonic generation microscopy: a powerful tool for bio-imaging” *Biophys Rev* **2023**, *15*, 43–70.
[42] F. Pavone, P. Campagnola, *Second Harmonic Generation Imaging*, CRC Press, **2016**.
[43] A. Krężel, W. Bal, “A formula for correlating p K a values determined in D 2 O and H 2 O” *Journal of Inorganic Biochemistry* **2004**, *98*, 161–166.
[44] S. Kim, A. Martínez Dibildox, A. Aguirre-Soto, H. D. Sikes, “Exponential Amplification Using Photoredox Autocatalysis” *Journal of the American Chemical Society* **2021**, *143*, 11544–11553.
[45] R. Van Kerrebroeck, P. Naert, T. S. A. Heugebaert, M. D’hooghe, C. V. Stevens, “Electrophilic Bromination in Flow: A Safe and Sustainable Alternative to the Use of Molecular Bromine in Batch” *Molecules* **2019**, *24*, 2116.
